# Molecular Correlates of Host Specialization in *Staphylococcus aureus*

**DOI:** 10.1371/journal.pone.0001120

**Published:** 2007-10-31

**Authors:** Lisa Herron-Olson, J. Ross Fitzgerald, James M. Musser, Vivek Kapur

**Affiliations:** 1 Department of Microbiology, Veterinary Pathobiology and Biomedical Genomics Center, University of Minnesota, Saint Paul, Minnesota, United States of America; 2 Laboratory for Bacterial Evolution and Pathogenesis, University of Edinburgh, Edinburgh, Scotland, United Kingdom; 3 Center for Molecular and Translational Human Infectious Disease Research, Methodist Hospital Research Institute, and Department of Pathology, Methodist Hospital, Houston, Texas, United States of America; Centre for DNA Fingerprinting and Diagnostics, India

## Abstract

**Background:**

The majority of *Staphylococcus aureus* isolates that are recovered from either serious infections in humans or from mastitis in cattle represent genetically distinct sets of clonal groups. Moreover, population genetic analyses have provided strong evidence of host specialization among *S. aureus* clonal groups associated with human and ruminant infection. However, the molecular basis of host specialization in *S. aureus* is not understood.

**Methodology/Principal Findings:**

We sequenced the genome of strain ET3-1, a representative isolate of a common bovine mastitis-causing *S. aureus* clone. Strain ET3-1 encodes several genomic elements that have not been previously identified in *S. aureus*, including homologs of virulence factors from other Gram-positive pathogens. Relative to the other sequenced *S. aureus* associated with human infection, allelic variation in ET3-1 was high among virulence and surface-associated genes involved in host colonization, toxin production, iron metabolism, antibiotic resistance, and gene regulation. Interestingly, a number of well-characterized *S. aureus* virulence factors, including protein A and clumping factor A, exist as pseudogenes in ET3-1. Whole-genome DNA microarray hybridization revealed considerable similarity in the gene content of highly successful *S. aureus* clones associated with bovine mastitis, but not among those clones that are only infrequently recovered from bovine hosts.

**Conclusions/Significance:**

Whole genome sequencing and comparative genomic analyses revealed a set of molecular genetic features that distinguish clones of highly successful bovine-associated *S. aureus* optimized for mastitis pathogenesis in cattle from those that infect human hosts or are only infrequently recovered from bovine sources. Further, the results suggest that modern bovine specialist clones diverged from a common ancestor resembling human-associated *S. aureus* clones through a combination of foreign DNA acquisition and gene decay.

## Introduction

*Staphylococcus aureus* (SA) causes a variety of serious diseases in multiple host species, including humans and cattle. Widespread antimicrobial resistance among human isolates of SA is cause for considerable concern, and has resulted in substantial increases in the cost of treatment associated with SA infection. While the implementation of improved hygiene practices, teat dipping, vaccines and antimicrobials on dairy farms have limited the spread of antimicrobial resistance among bovine-associated SA, mastitis continues to cause billions of dollars in economic loss to the U.S. dairy industry on an annual basis [1]–[3].

The molecular mechanisms by which SA causes bovine mastitis remain poorly understood. Importantly, studies of the molecular epidemiology of SA strongly suggest that a genetic subset of strains is particularly well adapted for causing infection in cattle [4]–[9]. Population genetic studies of thousands of SA isolates from multiple hosts and geographic locales reveal a clonal population structure, with strong evidence of host-specialization among clones associated with human and bovine infection [3], [10]–[14]. Among SA isolates, clones with distinctive genetic backgrounds are responsible for the majority of infections within a host type, although the distinct bovine clone groups are often interspersed among the human clone groups in a manner suggesting that human-associated SA clones were the evolutionary precursors to modern bovine specialist SA clones [11], [13], [15], [16]. Furthermore, longitudinal studies show that a limited number of genotypes account for persistent mastitis in cattle herds, underscoring the notion that certain SA genotypes are particularly successful at causing bovine mastitis [11], [13], [17]. However, despite its clinical importance from a human and animal health perspective, its well-described clonal population structure, and the availability of complete genome sequences from several isolates for use in comparative analyses [18]–[21], the molecular basis of host-specialization in SA remains unknown.

Thus, to begin to understand the molecular basis of virulence of SA clones associated with bovine mastitis and to identify the genetic features contributing to host specialization, we sequenced the complete genome of strain ET3-1, an isolate that represents the most common SA clone isolated from bovine mastitis worldwide [12]. Comparative whole genome analyses using DNA microarrays of the ET3-1 genome with that of other bovine-associated SA isolates and SA recovered from humans hosts shows that bovine specialist clones have accumulated substantial genetic divergence in genes previously associated with SA pathogenesis in the form of both genome decay and genome expansion. Further, our studies reveal a set of genomic features that distinguish clones of highly successful bovine-associated *S. aureus* optimized for pathogenesis in cattle from those that infect human hosts or those that are only infrequently recovered from bovine sources. Overall, these results provide a molecular population genetic framework for understanding the mechanism of host specialization in this important multi-species pathogen.

## Results

### Genome organization and mobile genetic elements

**Figure 1:**
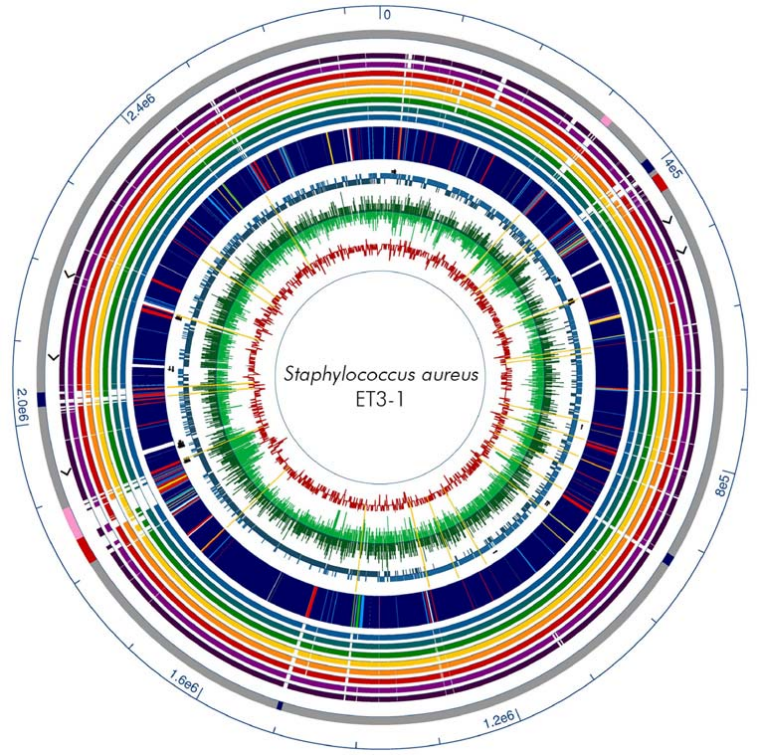
Comparative circular map of ET3-1 and other SA genomes showing (from outside) 1) Scale in basepairs, 2) Mobile elements (red = conserved island, blue = pathogenicity island, pink = putative phage), 3) Ribosomal RNA operons (arrowheads), 4) Homology to other SA genomes, from outside and with color code, dark purple = MRSA252, light purple = MSA553, red = NCTC8325, orange = COL, yellow = Mu50, green = N315, teal = MW2, light blue = MSSA476, 5) ORF homology of ET3-1; navy blue = strong homolog to other SA, light blue = intermediate homolog to other SA, green = weak homolog to other SA, red = non-SA homolog, yellow = no GenBank matches (e-value>10^−5^), 6) Location of tRNAs (small arrows), 7) ORF direction, light teal = forward strand, dark teal = reverse strand, 8) Microarray data from bovine isolates; light green inner portion = similar ET3 bovine isolates and dark green (outer) = non-ET3 bovine isolates. Yellow lines indicate insertion elements and transposases. 9) GC content = red graph line.

**Figure 2:**
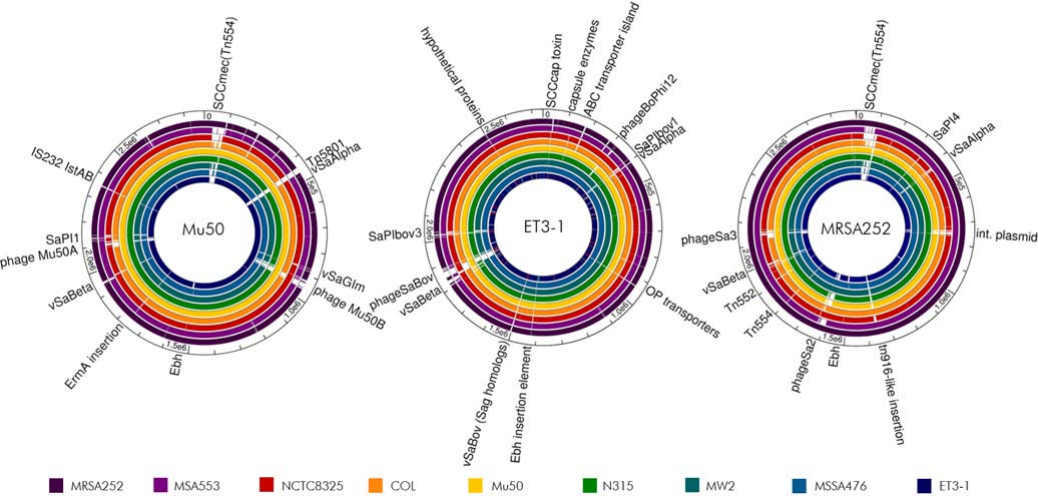
“Plug and play” genome organization in SA. Fully sequenced SA genomes were locally aligned using nucleotide BLAST; gaps in the colored histogram represent missing sequence (e-value>10^−5^). The alignments illustrate the conservation of the core genome and the importance of mobile genetic elements in SA genome variation. Similar genetic elements integrate in different locations; for example, phageSaBov in strain ET3-1 and bacteriophage Mu50β in strain Mu50 have homologous content but vary in their sites of integration. A linear path of mobile element acquisition is not evident among the sequenced strains, underscoring the role of lateral transfer in the evolution of SA.

The 2,742,531 base pair genome of ET3-1 (GenBank accession no. AJ938182) is the smallest of the nine sequenced SA genomes included in the analysis (Figure 1, Table 1). A total of 2,589 open reading frames was identified and annotated (Table S1). Thirty-three genes encode products with homology solely to proteins from organisms other than SA or SA-associated phages and an additional 80 predicted proteins have no significant protein homologs in public databases. As observed in other SA genomes, the majority of the ET3-1-unique genes that are unique to GenBank or newly described in SA are encoded by mobile genetic elements (MGEs). At least two of the MGEs have not been described previously, and several MGEs resemble islands that are integrated at different locations in the sequenced human SA isolates than in ET3-1 (Figure 2) [22]. Excluding the MGEs, the remainder of the genome closely resembles that of the fully sequenced SA isolates from humans with respect to gene content and organization. These results are consistent with prior studies that show conserved gene order in the genome backbone and high density of genetic variation within MGEs of Gram-positive bacterial pathogens [20], [23], [24]. This has led to the hypothesis of a “plug and play” mechanism whereby specific combinations of virulence factors encoded within MGEs are exchanged among strains, resulting in clones that are particularly well-adapted for causing certain diseases or infecting specific hosts ([23], Herron-Olson, Genomes 2004 Meeting, Hinxton, Cambridge, United Kingdom).

**Table 1:** SA strains evaluated in this study

Strain	ST	Host^A^	Origin	Size (Mb)	No. of Pseudo-genes^C^	MLEE type^D^	Ref.
**ET3-1**	151	Bovine	Ireland	2.74	70	3	[25]
**Mu50**	5	Human	Japan	2.87	0		[18]
**N315**	5	Human	Japan	2.81	0		[18]
**COL**	250	Human	USA	2.81	36		[21]
**NCTC8325**	8	Human	USA	2.82	2		[27]
**MSA553**	30	Human	USA	2.86	N/A		Herron-Olson et al., in prep.
**MW2**	1	Human	USA	2.82	0		[19]
**MSSA476**	1	Human	UK	2.80	40		[20]
**MRSA252**	36	Human	UK	2.90	88		[20]
**ET3-2**	new^B^	Bovine	USA			3	[11]
**ET3-3**	151	Bovine	Ireland			3	[12]
**PSA4**	97	Bovine	USA				[11]
**PSA6**	new^B^	Bovine	USA			39	[11]
**PSA10**	new^B^	Bovine	USA			1	[11]
**PSA13**	new^B^	Bovine	USA			1	[11]
**PSA17**	new^B^	Bovine	USA			5	[11]
**PSA20**	new^B^	Bovine	USA			7	[11]
**PSA72**	new^B^	Bovine	USA			36	[11]
**PSA1001**	97	Bovine	USA			23	[11]

^A.^Host: B = bovine, H = human

^B.^New MLST profiles are being submitted to the database

^C.^Based on annotation in NCBI database

^D.^Based on reference [11]

**Figure 3:**
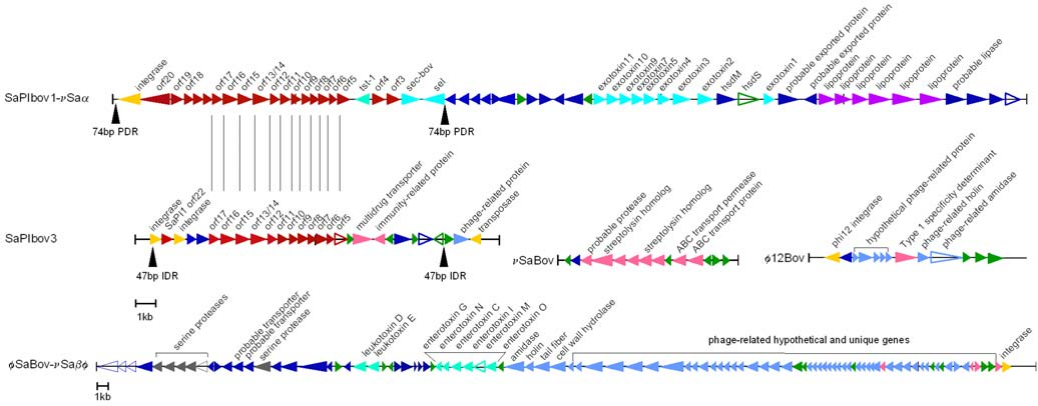
Gene content of major mobile genetic elements in ET3-1. SaPIbov1- νSaα, is shown aligned with the partially duplicated version of the pathogenicity island SaPIbov3 where vertical black lines represent homologous genes. Three additional unique ET3-1 MGEs, including νSaBov carrying several streptolysin homologs, φ12Bov and the large element φSaBov-νSaβ, are illustrated (note individual scales). Arrowhead colors: red = previously characterized SaPI gene of unknown function, turquoise = toxin, blue = SA homolog, green = no homologs, pink = homolog from non-SA microbe or phage, yellow = mobile element enzyme, light blue = phage protein, gray = protease, purple = lipoprotein, hollow triangle = putative pseudogene.

Strain ET3-1 was the source isolate for the discovery and characterization of SaPIbov1, a pathogenicity island associated with the bovine SA isolates that encodes the genes for TSST-1 and a bovine-associated variant of staphylococcal enterotoxin C [25]. We discovered that SaPIbov1 is integrated adjacent to the conserved genomic islet νSaα (Figure 1). Previous studies of SaPIbov suggested that a portion of the island was duplicated [25]. Our investigation revealed that the duplicated portion encompasses 12 genes (SAB1894-SAB1906) encoded within a second island in strain ET3-1 that is referred to as SaPIbov3 (Figure 3). The duplicated genes share an average of 93.3% amino acid identity. To our knowledge, this is the first report of a partially duplicated pathogenicity island within a SA genome. In total, SaPIbov3 consists of 17,945 basepairs encoding 28 genes, including 4 unique genes and 6 genes with homologs in other Gram-positive organisms. Transposase and integrase gene homologs (SAB1883 and SAB1910) border SaPIbov3, located at an integration hotspot which encodes φSa3 or SaPI1 in other SA strains (Figure 2, [19], [20]). No exact direct flanking repeats were identified, however, two 47-bp regions with 82% sequence identity lie between the third and fourth genes of SaPIbov3 and within the 3′ end of the terminal integrase.

Several other MGEs in the genome of strain ET3-1 encode genes that have not been previously identified in SA. The small genomic islet νSaBov encodes 14 genes novel to SA, including two homologs of streptolysins, the potent leukotoxins of the Sag locus originally identified in *Streptococcus pyogenes* GAS (Figure 3, SAB1372-SAB1383, [26]). The streptolysin homologs (SAB1376 and SAB1373) have 31% and 27% amino acid identity, respectively, to the corresponding genes encoding SagB and SagD in GAS strain M1 [26]. The 6,351-bp island is integrated at a position in the genome equivalent to that of φSa2 in MW2 and MRSA252 [19], [20], although no clear evidence of horizontal transfer was identified. Phages contribute to the unique gene content of ET3-1; φSaBov encodes 8 unique genes and is located adjacent to the genomic islet νSaβ in ET3-1. This phage resembles φ11/φETA and φMu50β carried by the MRSA isolate Mu50, although their integration sites differ (Figure 2). In the site where φMu50β is integrated in strain Mu50, strain ET3-1 encodes a transposase and several oligopeptide transporter genes that lie within an island previously identified as variable among diverse SA isolates [18], [19], [24], [27]. Another phage, φ12Bov, resembles the well-characterized SA φ12 but like φSaBov, encodes several unique genes and genes with homologs in other Gram-positive organisms (Figure 3).

### Gene content differences between ET3-1 and previously sequenced SA isolates associated with human infections

The majority of the more than 2000 non-MGE-associated genes shared among the SA genomes show a high degree of conservation in gene order and sequence. The ratio of the rate of nonsynonymous substitution to synonymous substitution (dn∶ds) among homologous gene pairs between ET3-1 and other sequenced SA genomes was calculated in order to identify divergent genes (Table 2). A dn∶ds ratio less than 1 is typical for most genes, and suggests that DNA mutations resulting in an amino acid replacement are selected against, presumably due to the deleterious effects of the change on protein function [28]. The majority of homologous gene pairs between ET3-1 and sequenced SA isolates associated with humans showed a low dn∶ds ratio (average = 0.29±0.74) and a trend toward the conservation of protein sequence. In contrast, many genes involved in host colonization, toxin production, gene regulation, iron metabolism and antibiotic resistance had an almost 10-fold greater average dn∶ds ratio in the ET3-1 genome relative to human associated sequenced SA isolates (Table 2).

**Table 2:** Amino acid substitution rates for selected ET3-1 proteins

Strain	Avg. Rate Ratio*	Origin	Role
**clfA**	2.01±0.63	clumping factor A	adhesion
**sdrC**	2.35±0.79	Ser-Asp rich binding protein	adhesion
**fnbA**	2.86±1.40	fibronectin binding protein A	adhesion
**SAB0513**	1.26±1.13	Ser-Asp rich binding protein	adhesion
**SAB0169**	2.03±0.47	staphylocoagulase precursor	adhesion
**SAB0745**	4.09±1.42	secreted Von Willebrand factorbinding protein	adhesion
**spa**	1.60±0.38	IgG-binding protein	virulence
**set11**	2.00±0.64	staphylococcal enterotoxin 11	virulence
**set9**	1.12±0.77	staphylococcal enterotoxin 9	virulence
**SAB2172**	3.18±2.70	secretory antigen precursor	virulence
**SAB0547**	5.45±N/A	probable membrane protein	unknown
**SAB2190**	2.05±0.08	probable membrane protein	unknown
**SAB2220**	3.10±0.32	probable membrane protein	unknown
**SAB2582**	2.35±0.90	probable membrane protein	unknown
Total, over all genes	0.29±0.74		

Avg. Rate Ratio = average ratio of rate of nonsynonymous substitution to rate of synonymous substitution from ET3-1 gene versus homolog in MRSA252, N315 and MW2

Interaction with host tissue plays a critical role in establishing mastitis infection [9]. Several genes in strain ET3-1 that encode predicted surface proteins involved in colonization and host protein binding show higher-than-average rates of nonsynonymous substitution and gene decay relative to their homologs in the sequenced human SA isolates. The ET3-1 genes with the highest dn∶ds ratios include fibronectin-binding protein (*fnbA*), staphylocoagulase precursor, secreted Von Willebrand factor-binding protein and several additional genes encoding host protein-binding motifs (Table 2).

**Figure 4:**
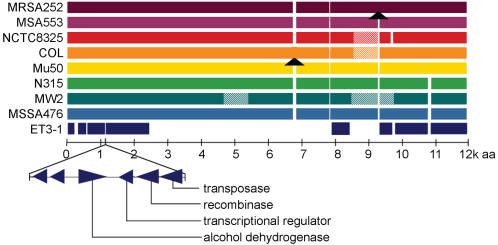
Variation in the fibronectin-binding protein Ebh. Colored bars represent the amino acid alignment of the Ebh proteins from the sequenced SA isolates as labeled, including the sequenced bovine isolate ET3-1. Striped segments show areas of sequence variation; arrowheads indicate the insertion of 10 or more amino acids. The remnants of *ebh* in ET3-1 comprise 5 separate transcripts and are punctuated by a small mobile element encoding 6 genes with homologs on plasmid pN315 (from SA isolate N315) and in *S. epidermidis*.

We discovered that genes encoding several well-characterized virulence factors such as the antibody-binding protein *spa* and clumping factor A (*clfA*) contain premature stop codons, and thus are pseudogenes in strain ET3-1. Genes encoding at least six other predicted surface-expressed structural and adhesion proteins, including *sdrC*, *ebh,* and the peptidoglycan component *fmhA* also have premature stop codons, and seven ET3-1 pseudogenes with conserved non-truncated homologs among the sequenced human isolates encode hypothetical proteins with export or cell surface-localization signals. The *spa*, *ebh*, *fmhA*, and *sdrC* polymorphisms were verified by PCR and conventional capillary DNA sequencing in several bovine mastitis-associated SA strains. All of the premature truncation polymorphisms except for that of *sstC* were present in strain ET3-2, another successful bovine clone; however, premature truncations were generally not conserved in the less successful bovine mastitis-associated isolates (Table 3). In some genes, the decay is extensive; *sdrC* encodes two premature stop codons, resulting in three putative transcripts separated by sizeable intergenic regions [29]. The gene for the very large surface protein *ebh* is interrupted in multiple locations, resulting in five predicted transcripts separated by noncoding intergenic regions [SAB1289-SAB1293 and SAB1300; Figure 4; [30]]. ET3-1 is the only SA genome sequenced thus far in which the majority of *ebh* is actually missing from the genome, with the remaining fragments constituting only about 30% of the full-length gene. The absence of the large internal portion of *ebh* was confirmed by PCR analysis in several other bovine mastitis-associated SA isolates. The truncations may have been caused by the insertion of a mobile genetic element, since sequencing revealed a transposase and 5 genes with homologs in *S. epidermidis* and on SA plasmid pN315 inserted into the *ebh* region. The inserted genes (SAB1294-1299) include homologs of 2 hypothetical proteins, an alcohol dehydrogenase, a transcription factor, a recombinase and the transposase for IS431. Taken together, these features indicate that gene decay in the successful bovine isolates has been ongoing for some time.

**Table 3:** Pseudogene verification among diverse bovine-associated SA isolates

		ET3 isolates			Non-ET3 isolates		
Gene	Product	ET3-1	ET3-2	ET3-3	PSA10	PSA20	PSA1001
**spa**	antibody-binding protein A	P	P	P	P	C	P
**sstC**	iron transport system protein	P	D	D	D	D	D
**sdrC1**	host protein binding protein	P	P	D	C	C	C
**sdrC2**	host protein binding protein	P	P	D	C	C	C
**fmhA**	peptidoglycan component	P	P	N/A	C	C	N/A

P = pseudogene conserved as in ET3-1

D = divergent sequence, possibly pseudogene but different than ET3-1

C = conserved sequence, not pseudogene, matches human SA isolates

Toxins play an important role in staphylococcal pathogenesis, and the ET3-1 genome encodes at least 30 toxin genes (Table S1), including the pyrogenic toxin superantigen TSST-1 and the bovine variant of staphylococcal enterotoxin C [25]. Other toxin homologs such as the staphylococcal enterotoxins *set9* and *set11* show high levels of allelic variation relative to other sequenced SA homologs (Table 2). Previous studies suggest that superantigens and leukotoxins are important during mastitis pathogenesis due to their immunomodulatory effects [31], [32]. At least three sets of two-component leukotoxins are encoded in ET3-1, including *lukM*/*lukF'-PV*, which is associated with horizontal transmission by temperate bacteriophages of bovine origin (SAB0782 and SAB0783, [33], [34]). Recent investigations of LukM/LukF'-PV indicate that the assembled leukotoxin is several times more potent against bovine leukocytes than any other staphylococcal leukotoxins, including the Panton-Valentine leukotoxin that is highly active against human leukocytes [6], [35]. The aforementioned putative streptolysin homologs encoded in ET3-1 genetic islet νSaBov are also newly described among SA genomes. Two additional toxin homologs, SAB0026 and SAB2421, are unique among sequenced SA isolates. SAB2421 shows some homology (30% amino acid identity) to pyrogenic exotoxin G (SpeG) of *Streptococcus pyogenes*, while the gene encoded by SAB0026 shares 97% amino acid identity to an enterotoxin identified in the capsule type 1-associated cassette chromosome element SCCcap1 that has not been identified in a fully sequenced SA isolate previously. In ET3-1, the enterotoxin is integrated at *orfX*, a location associated with the integration of staphylococcal cassette chromosome elements, and the remainder of the SCCcap1 cassette is missing [36].

Antibiotic resistance among bovine SA is substantially less common than among human SA. Several recent studies show a decreasing incidence of antibiotic resistance among pathogens associated with bovine mastitis during the past 10 years, despite the use of antimicrobial therapy on dairy farms, and contrary to the substantial increase in antimicrobial resistance among human-associated SA [2], [37]–[39]. ET3-1 is susceptible to most antibiotics, excluding spectinomycin (64 µg/ml). A large majority (>90%) of the ∼800 proteins present in sequenced human-associated SA clones but absent from ET3-1 encode antimicrobial resistance determinants or hypothetical proteins associated with resistance-carrying MGEs. The ET3-1 genome encodes the lowest number of antibiotic resistance determinants of any of the sequenced SA genomes.

Iron is a key limiting reagent during *in vivo* bacterial infection, and multiple systems have been identified in SA for binding and scavenging iron from mammalian host iron-binding proteins (IBPs) such as lactoferrin, transferrin and hemoglobin [40]–[42]. While recent studies suggest that heme is a preferred iron source for SA during human infection, a predilection for particular iron sources has not been extensively studied in bovine SA [43], [44]. Our analysis of the ET3-1 genome suggests that the genes involved in iron acquisition and metabolism vary considerably relative to their homologs encoded by SA recovered from humans. For example, despite high sequence conservation among most metabolic enzymes encoded within the ET3-1 genome, several genes encoding proteins involved in cation transport and metabolism (ferric hydroxamate receptor, multicopper oxidase protein, cadmium resistance proteins CadD and CadX) are missing. Furthermore, a premature stop codon in the siderophore transporter ATPase *sstC* (SAB0687) and a number of polymorphisms in other iron-regulated genes, including cell surface transferrin-binding protein *isdB* (SAB0994) and the iron-regulated surface protein encoded by SAB1590, suggest that iron metabolism in the bovine clone is considerably different from that previously described in SA.

Interconnected SA regulatory systems control virulence factor expression during infection, however, very little is known regarding the function and interaction of SA regulatory genes during bovine mastitis [45], [46]. Importantly, a single alteration in a regulatory system component could alter expression of hundreds of genes. The quorum-sensing *agr* system consists of four genes, *agrABCD* and the effector molecule, RNAIII [47]–[49]. Four major *agr* groups have been described based on sequence divergence within the locus [50], [51]. In ET3-1, *agrD* and *agrB* closely match that of allele group II, however, the 5′ end of the *agrC* locus differs from all 4 major *agr* groups. A recent study of several hundred isolates of human and bovine origin also suggests that the *agr* locus differs between the two host origin groups, setting up a plausible scenario of cross-inhibition by molecules produced by different strain types that may further explain the genetic divergence of bovine mastitis-associated SA clones [46], [52]. The ET3-1 genome also encodes at least 4 transcription factor homologs not previously identified in a SA chromosome. SAB1297, embedded in the fragmented *ebh* gene region, encodes a MarR family transcription factor homolog with high sequence identity to a gene on SA plasmid pN315. SAB1256 shares 54% amino acid identity with transcriptional regulator PaiB from several *Bacillus spp*., while SAB1757 shares 40% amino acid identity with the LexA repressor from *Bacillus cereus* and lies within φSaBov. A fourth transcription factor homolog, SAB2083, most closely resembles a *Listeria* and *Bacillus* regulatory factor (43% aa identity) and is not associated with a MGE.

### Comparative genomic analysis with other human and bovine-associated SA

**Figure 5:**
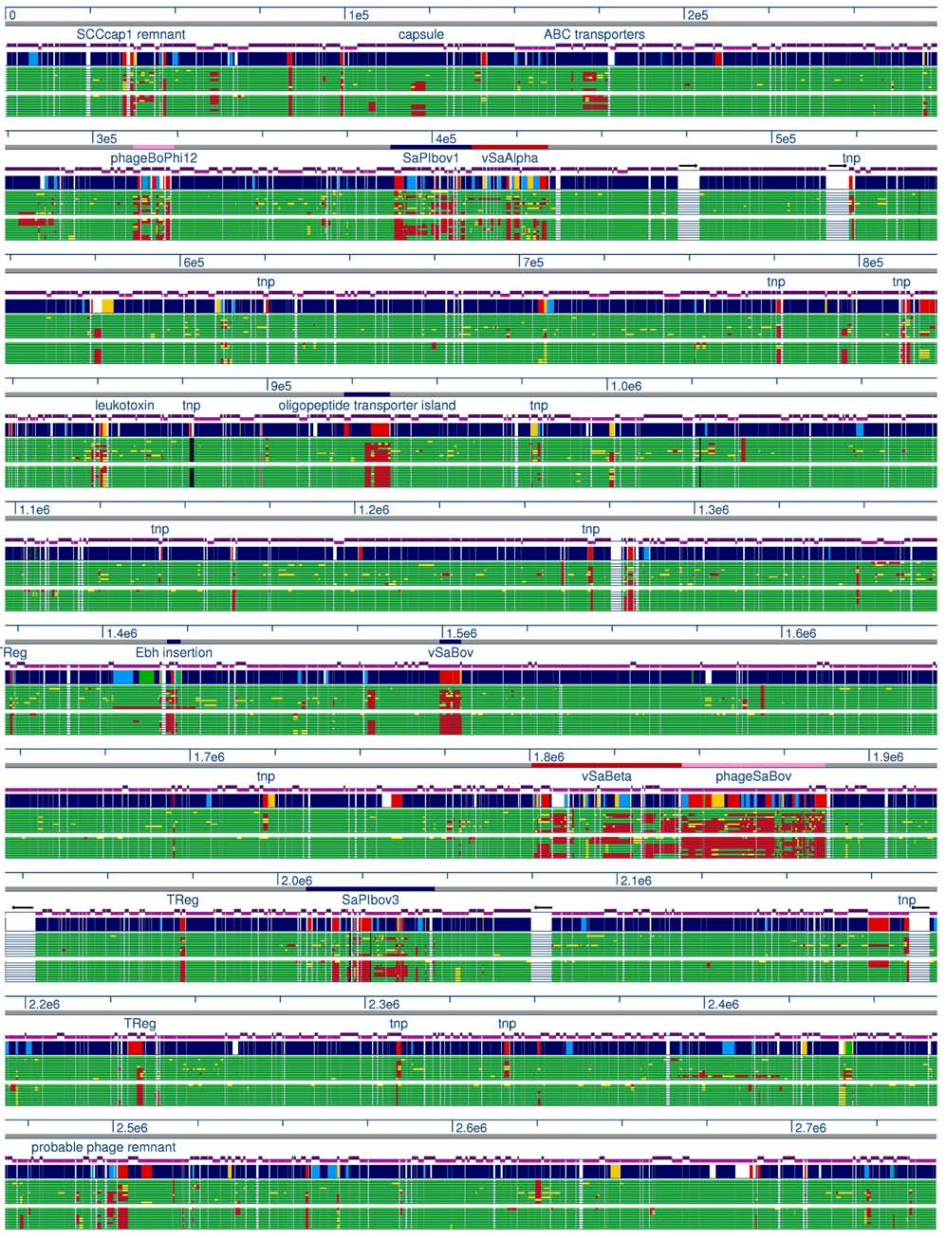
Linear comparative genomic analysis of SA associated with bovine and human infection illustrates hotspots of variation. The ET3-1 genome (blue/multi) was aligned with the microarray probe data from the 10 bovine SA isolates (top section of green histograms) and the Smith-Waterman alignment [75] of the microarray probe with the 8 completely sequenced human SA isolates (bottom section of green histograms). The scale, in nucleotides, is shown in blue. Mobile genetic elements are annotated above each histogram block and ribosomal RNA operons are represented as large white gaps in the green histograms. The purple histogram above the ET3-1 genome representation shows the orientation of the ORFs. For the green microarray and Smith-Waterman alignment histograms, green = present, yellow = divergent, red = absent. For the blue/multi histogram showing ORF homology of ET3-1; navy blue = strong homolog to other SA, light blue = intermediate homolog to other SA, green = weak homolog to other SA, red = non-SA homolog, yellow = no GenBank matches (blastp e-value>10^−5^).

The molecular population genetic methods that elucidated the clonal population structure of SA use highly conserved housekeeping genes and are not optimal for differentiating strains with similar genomic backbones that encode substantially different sets of MGEs, particularly when the MGEs are recently acquired. To investigate the genome content of additional SA isolates associated with bovine mastitis, we used a comparative whole-genomic DNA hybridization microarray composed of oligonucleotide probes for each ORF in the ET3-1 genome and 8 other fully sequenced SA genomes to survey 10 additional SA isolates from cattle (Figure 5). The cattle isolates were selected based on their genomic backgrounds as determined by multilocus enzyme electrophoresis (MLEE) and MLST (Table 1). Isolates ET3-2 and ET3-3 belong to the same clone group as ET3-1, the most common clone group associated with bovine mastitis worldwide and accounting for 29% of the total isolates recovered from cattle in a large population genetic study of SA [11]. Isolates PSA6, PSA10, PSA13, PSA17, PSA20 and PSA72 were chosen to represent 4 additional common bovine clones that are less prevalent than the ET3 clone type but still account for more than 78% of the remaining non-ET3 cattle isolates. PSA4 and PSA1001 were selected because their molecular typing profiles indicate that these isolates represent the relatively rare bovine clones (Table 1, [11]). Multilocus sequence typing of these bovine isolates confirmed the MLEE results showing that some bovine SA clone types, including the successful clone represented by PSA20, are genetically more similar to the sequenced human SA isolates than to other SA isolates commonly recovered from cattle (Figure 3).

**Figure 6:**
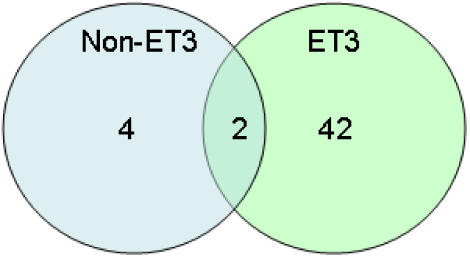
Venn diagram summarizing the unique gene content among bovine mastitis-associated *S. aureus*. In total, 47 microarray probes corresponded to gene sequences that are unique to ET3-1 among the sequenced *S. aureus* genomes. Nearly 94% of these genes were conserved in all of the ET3 isolates, while only 2 of these ‘unique’ genes were conserved among all 11 isolates of bovine origin.

Genomic DNA microarray hybridization data using the bovine SA isolates indicated that 44 of the 47 probes corresponding to unique ET3-1 gene sequences that were not previously identified in SA are also present in the closely related isolates ET3-2 and ET3-3 (Figure 6). Only two of the genes that are absent from the other sequenced isolates, only two of the genes are shared among all of the bovine mastitis-associated SA isolates based on microarray analysis. In total, four of the 3840 probes on the microarray were present in all of the non-ET3-1 isolates and were absent from all of the ET3-1 isolates. Hence, relatively few genes appear to be consistently present in bovine-associated SA and absent in human-associated SA, whereas a larger set of genes that are unique to bovine-associated SA appear to be conserved among the highly successful ET3 isolates.

**Figure 7:**
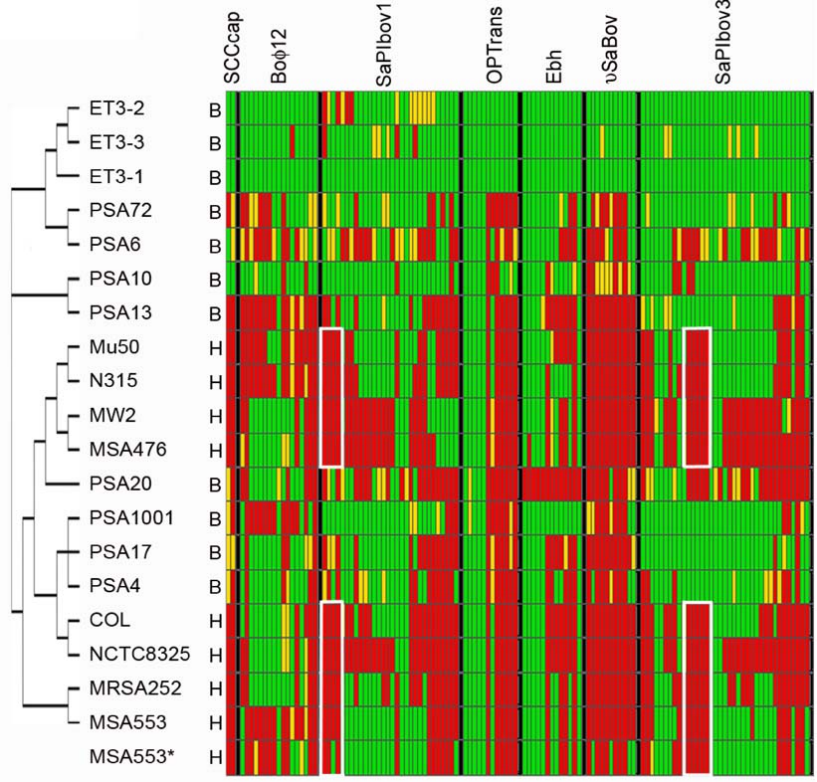
Comparative genomic DNA hybridization and *in silico* comparison of gene content within mobile elements of bovine and human SA isolates. In the MLST-based cladogram generated by PAUP [74], [76] at the left, “B” denotes a strain originally isolated from a bovine; “H” denotes isolation from a human. All data associated with “B” strains and MSA553^A^ are microarray hybridization data; all remaining “H” strains show the results of *in silico* Smith-Waterman alignment of the 70mer microarray probe to the genome sequence. Red = absent, yellow = indeterminable/variable, green = present. OPtrans = oligopeptide transporter island and Ebh = region of probes representing the *ebh* gene or gene remnants+probes for 6 gene insertion in ET3-1. The white boxes within the SaPIbov1 and SaPIbov3 islands show gene clusters that were consistently absent from strains associated with human infection and largely conserved in strains recovered from bovine sources, including those that more closely resemble the isolates from humans.

The microarray data (Figure 7) illustrate that the most diverse genetic regions, including SaPIbov, SaPIbov3, νSaBov, φSaBov, the insertion in the *ebh* gene and the SCCcap-associated toxin, are highly conserved in closely related and highly successful bovine SA clones. The strong correlation between the MGE content and molecular typing using housekeeping genes suggests that the MGEs in bovine SA may not be horizontally transferred at high frequency, in which case we would expect the MGE content of clones to be distributed independently of the typing profile. The correlation between MGE gene content and molecular typing also suggests that a specific combination of MGEs may enhance SA pathogenesis in cattle, since the MGE content among the most highly successful bovine SA clones is very similar. Interestingly, microarray analysis reveals that a set of 5 contiguous genes within SaPIbov3 (SAB1888-1892), 4 of which are duplicated in SaPIbov1, are consistently present in a majority of the isolates from cattle but are absent from previously sequenced human SA isolates. This includes the bovine SA isolates that are genetically more similar to human SA isolates by molecular typing (Figure 7). Overall, the microarray data show that much of the MGE content found among bovine SA is not shared with the sequenced human-associated SA genomes that were surveyed. The majority of these genes encode proteins of unknown function, so a precise role in bovine SA pathogenesis is undetermined.

## Discussion

The environment of the bovine teat canal and udder, including the presence of milk during lactation, differs markedly from common sites of infection in human hosts, and this may account for the high degree of genetic divergence identified among genes encoding surface-expressed proteins in strain ET3-1 relative to the human-associated SA isolates. Microarray studies of SA isolates collected from humans also identified lineage-specific surface protein features [53]. Furthermore, although unique or divergent MGEs have been identified in each SA genome sequenced thus far, gene homologs among the novel MGEs and ET3-1-unique genes identified in this study suggest that these elements may have been acquired from other mastitis-causing pathogens in the bovine environment. Additional studies of SA clones commonly associated with human and ruminant infection are recommended to investigate the role of MGEs in contributing to the tropism of some clones for infecting a certain host and causing a particular disease.

Our data suggest that the MGE content is similar among highly successful bovine SA clones that are closely related by MLST. These findings stand in contrast with prior studies indicating that human-associated SA isolates such as N315 and Mu50 are closely related by MLST, but differ considerably in MGE content, and they are consistent with the idea of relatively recent dissemination of a successful clonal bovine lineage. Alternatively, bovine SA may not undergo change as rapidly as human-associated SA isolates; the substantially lower number of genes associated with antibiotic resistance in the genome of strain ET3-1 provides additional evidence for a slower rate of genetic exchange. Overall, the conservation of gene content among the MGEs of highly successful bovine SA isolates illustrated by the microarray study is consistent with evidence from prior studies and suggests a plug-and-play mechanism whereby a particular combination of MGEs enhances pathogenicity in a specific infection model [20]. Additional studies of SA genomes that feature divergent backbone content and similar MGE combinations would be required to further explore this mechanism.

A second very unexpected finding was the high level of gene decay observed among dozens of ET3-1 genes, including several well-characterized virulence factors that are conserved among the sequenced SA isolates associated with human infection. Gene decay has been observed among other SA genomes, but the quantity and the genes affected differ among strains. Strain MRSA252 is the most divergent of the sequenced human-associated SA isolates by MLST and encodes the largest number of annotated pseudogenes (n = 88). Strain ET3-1 encodes 70 annotated pseudogenes, whereas the other sequenced SA genomes encode substantially fewer pseudogenes (Table 1). Gene decay frequently results from random mutation, but may also be caused by insertion sequences or slip-strand mispairing resulting from short sequence repeats in the upstream or 5′ region of the gene [54], [55]. A search for simple sequence repeats (<9 bases) within the ET3-1 genome found no correlation with pseudogenes, although many of the ET3-1 pseudogenes did contain longer repeat motifs of 20 to 60 bases that are likely related to their surface localization and host-protein binding function. Although insertion sequences are common in SA, they account for less than four percent of the pseudogenes in ET3-1; hence, the pseudogenes in the ET3-1 genome likely arose by random mutation.

Gene decay has been described as a mechanism of adaptation among obligate intracellular and enteric Gram-negative pathogens, including *Helicobacter pylori*, *Yersinia pestis*, *Mycobacterium leprae* and others [55]–[59]. In *Y. pestis*, genetic divergence appears to be driven by gene decay via insertional and point mutation, in combination with selective genome expansion by lateral gene transfer among plasmids. In contrast, ET3-1 contains no plasmids and the acquisition of MGEs has not yet led to substantial genome expansion on the scale of that observed among *Yersinia*; in comparison with other sequenced SA genomes, ET3-1 is substantially smaller (Table 1). Alternatively, gene decay has been associated with the transition to an intracellular lifestyle, whereby biosynthetic components are lost in favor of parasitizing nutrients from the host cell. Substantial evidence suggests that SA can exist as an intracellular pathogen, and that intracellular survival may be particularly important during bovine mastitis [60]–[63]. Our data support a scenario in which ET3-1 and similar SA clones are beginning the transition to an intracellular lifestyle, during which the role of adhesion and host protein-binding components is reduced and these components are being lost. A transition to a largely intracellular lifestyle would also mimic a reduced capacity for lateral gene transfer, because contact with other microbes with which genetic material could be exchanged would be reduced. An intracellular lifestyle may also account for the paucity of antibiotic resistance determinants, which would be unnecessary for bacteria living within host cells, and could also explain the observed allelic polymorphisms among genes involved in iron metabolism in ET3-1. Iron availability within cells is substantially different than in the extracellular milieu, where iron is scarce and often tightly bound to host iron-binding proteins (IBPs).

Another explanation for the divergence in iron metabolism components observed in the ET3-1 genome relates to the structure of mammalian IBPs, which differ substantially among species. These differences provide a selection pressure for pathogens in which adaptation to enhance iron scavenging from host-specific IBPs contributes to virulence [64]. For example, host-specific expression of transferrin-binding proteins in the protozoan pathogen *Trypanosoma brucei* in human, canine and bovine hosts has been described, suggesting that the relationship between host IBPs and pathogen scavenging systems is specific and important during infection [65]. Furthermore, host IBPs are specific to certain tissues and anatomical compartments, such as the bovine udder, where most of the iron is bound to lactoferrin or ferric citrate and the balance of the two iron-containing molecules changes during different stages of lactation. By contrast, during most human SA infections, the pathogen is primarily exposed to IBPs such as hemoglobin and transferrin, and human-associated SA isolates appear to prefer heme as an iron source [43]. The iron metabolism of common bovine SA clones therefore warrants further study of its role in exuding host-specificity and enhancing pathogenesis in bovine mastitis.

Overall, we identified more extensive variation between bovine and human-associated *S. aureus* genomes than that found amongst human-associated *S. aureus*, revealing a set of genomically encoded components that distinguish populations of highly successful human and bovine-associated *S. aureus* clones that are optimized for infection of different hosts. Furthermore, the results support prior population genetic studies suggesting that modern bovine specialist clones diverged from a common ancestor resembling human-associated *S. aureus* clones, and have since exchanged genetic material independently of human-associated *S. aureus*. These findings provide a strong foundation for investigations of the molecular basis of pathogenesis of SA-associated bovine mastitis, as well as a rational foundation for continuing the investigation of host specificity in this important human and animal pathogen.

## Materials and Methods

### Isolate selection

The bovine SA clone selected for sequencing was isolated from a cow presenting with clinical mastitis in 1993 [12]. The clone is of the ET3 profile (and for simplicity is therefore referred to as ET3-1 in the body of the manuscript), the most common electrophoretic type associated with bovine mastitis, which is exclusive to cattle and distributed worldwide [11], [12]. The clone also belongs to the ST151 MLST type (Table 1). A novel 15,981-bp pathogenicity island, SaPIbov1, was identified in this isolate [25].

### Library preparation

A shotgun sequencing approach was utilized to initiate whole genome sequencing. Isolated whole genomic DNA from SA strain ET3-1 was randomly sheared to generate fragments of 1.7–2.4 kb using a HydroShear apparatus (GeneMachines, San Carlos, CA); fragment size was verified by agarose gel electrophoresis. The ends of 200 ul (approximately 25 µg) of the randomly sheared fragments were repaired using T4 polynucleotide kinase and Klenow enzyme (New England Biolabs, Beverly, MA) at 37°C. Enzymes were deactivated at 75°C for 10 minutes and the end-repaired fragments were run on a 0.8% ethidium bromide agarose gel. DNA of 1.8 to 2.2 kb was excised from the gel and purified using the QiaEXII Gel Extraction Kit according to the manufacturer's protocol (Qiagen, Valencia, CA). ET3-1 DNA fragments (approximately 850 ng) were ligated into *Sma*I-restricted pUC18 (Stratagene, LaJolla, CA) using T4 ligase (New England Biolabs). 4 µl of the ligation product were transformed into XL10-Gold Epicurian Coli ultracompetent cells according to the published protocols (Stratagene). Blue colonies were picked from LB-ampicillin/X-gal/IPTG plates after 24 hours at 37°C and incubated in 96-well culture blocks, each well containing 1.25 ml LB-ampicillin broth (50 µg/ml). Cells were pelleted after 21 hours by centrifugation at 1500×*g* for 5 minutes. Plasmids were purified using the 96 Turbo Miniprep preparation, according to the protocols of the manufacturer (Qiagen).

### Shotgun sequencing and assembly

Plasmids (approximately 100 ng each) were transferred to new 96-well plates, dried overnight and resuspended with 6 picomoles of primer (T7), then sequenced on ABI 3700 automated DNA capillary sequencers (ABI, Foster City, CA). The initial shotgun sequencing approach yielded a total of 23,611 sequences (6.5-fold coverage) which were assembled into contigs using PhredPhrap [66], [67]. The original shotgun sequence assembly, using a minmatch value of 12 and force level of 8 resulted in a total of 127 contigs greater than 2 kb. Within the initial shotgun assembly, 65.6% of the cumulative sequence was represented in contigs greater than 10 kb. In order to expedite the genome assembly, a novel software tool called MGView was developed to graphically overlay the assembled contigs onto completed SA genomes that were available publicly [68]. Contig joins suggested by MGView were used to design primers spanning assembly gaps for amplification by either regular PCR (up to 3 kb) or XL-PCR (up to 15 kb) followed by sequencing. For regular PCR, each 25 µl reaction contained 1 unit of Taq polymerase (Applied Biosystems) with appropriate manufacturer-supplied buffer at 1X, 1.5 mM MgCl, 0.1 µM primer (each), 200 µM dNTP (each) and 200 ng genomic ET3-1 DNA. Thermal cycling conditions were as follows: Denature at 95°C×30 seconds, anneal at 54°C×30 seconds and extend at 72°C×1 minute (repeat for 25 cycles), final extension at 72°C×8 minutes. For XL-PCR, TaqStart PCR polymerase-binding antibody (ClonTech, Mountain View, CA) was utilized according to the manufacturer's recommendations. Each 35 µl reaction also contained 5 units of r*Tth* XL DNA polymerase (GeneAmp XL PCR, Applied Biosystems) with supplied buffer at 1X containing glycerol and DMSO, 1.5 mM MgOAc_2_, 200 µM dNTP (each), 0.2 µM primer (each), and approximately 200 ng genomic ET3-1 DNA. PCR thermal cycling was conducted as follows: A) Pre-denature at 95°C×10 min.; B) Cycling: denature at 95°C×10 min, anneal/extend at 60°C×9 min+15 second increments added each round; repeat for 20 cycles; C) Final extension: 72°C×10 min. All PCR products were verified by gel electrophoresis, purified using Microcon 100 filter plates (Millipore, Billerica, MA) and sequenced according to conditions optimized for template length, template type, and template and primer GC content, which were determined from extensive testing in the Advanced Genetic Analysis Center at the University of Minnesota. Using these optimizations, single sequence reads of 750–1000 bp from single regular and XL-length double-stranded PCR-derived DNA templates were consistently obtained, without the need for subcloning or large-insert libraries. For most XL-PCR products, additional sequencing primers were generated in order to cover the entire length of the product, and these primers were determined based on homology with other SA genome sequences. Where this approach was unsuccessful due to sequence variation between ET3-1 and other SA genomes, new sequencing primers were generated along the length of the template following the acquisition of the new sequencing reads.

As the PCR products were added, the assembly was continuously updated, proofread and edited using the software CONSED [69]. False joints in the assembly were taken apart (e.g., using the “tear contig” function), then reattached in the correct orientation (“join contig” function). Once false assembly joints were identified, PCR products corresponding to the correct joint were generated and sequenced in order to verify the accuracy of the assembly. The addition of these sequences to the assembly acted as a ‘patch’ to reinforce the corrected assembly. Where the software repeatedly misassembled joints even after the addition of PCR patches due to ubiquitous repeat sequences, longer artificial reads covering the sequence flanking the repeat were used to guide the assembly. The final gaps that could not be closed using the MGView-guided directed PCR procedure (n = 14) corresponded to sequence assembly regions that were unique to strain ET3-1 relative to other available SA genome sequences, and were closed by random PCR. Closures and weakly covered regions were verified by PCR and sequence analysis, resulting in a final error rate of below 1 error per 10,000 bases [as estimated by CONSED [69]]. In total, more than 1500 additional sequence reads from the PCR-based closure and proofreading strategy were added to the shotgun assembly.

### Annotation and computational comparative genomics

Open reading frames (ORFs) were identified using ARTEMIS [70] followed by manual verification of start sites based on GC skew and location of Shine-Dalgarno sequences [71]. Annotation of products was performed manually using evidence gathered from BLASTP searches against a microbial sequence database devoid of SA genome sequences in order to prevent bias [72]. The final pass of sequence annotation included the completed SA genomes for comparison [18]–[20]. The sequence divergence between homologous gene pairs in ET3-1 and the fully sequenced human SA isolates was estimated by calculating the nonsynonymous and synonymous substitution rate ratio (dn∶ds) for each gene pair shared between two sequenced genomes [28]. A dn∶ds less than 1 suggests that DNA mutations resulting in an amino acid change are not conserved in subsequent generations, presumably due to the deleterious effects of the mutation on protein function, whereas a dn∶ds greater than 1 suggests that mutations resulting in amino acid changes do not negatively impact the organism and that the gene product may be under pressure to change. For this study, dn∶ds was calculated for each gene pair shared between ET3-1 and the sequenced human SA isolates MRSA252, N315 and MW2 [18]–[20]. Rates of synonymous and nonsynonymous substitution were calculated by first identifying orthologs between bovine ET3-1 and other sequenced isolates by BLASTP, then refining the sequence alignments using CLUSTALW and calculating the rates by Nei and Gojobori's algorithm [28], [72], [73].

### Microarray analysis

Genome sequences from bovine strain ET3-1 and all available SA human isolates (Table 1) were used to construct a nonredundant 3840 probe 70mer DNA oligonucleotide array. Probes were synthesized by Illumina (San Diego, CA); dried oligos were resuspended in 3XSSC and spotted in triplicate onto Corning Gaps II aminosaline-coated microarray slides (Corning, Big Flats NY) using a BioRobotics Microgrid II Array Spotter (Genomic Solutions, Ann Arbor MI). Slides were rehydrated, UV cross-linked and stored under dessication until use in hybridization studies.

Bovine SA isolates for comparative genomic hybridization were selected based on previously identified multilocus enzyme elecrophoresis (MLEE) and multilocus sequence typing (MLST) profiles determined according to established protocols (Table 1, [74]). The SA isolates were taken from the original clone collection used for MLEE-based population genetic analysis of bovine cattle and were primarily of U.S. origin; specific information on the nature of the clinical manifestation (e.g., acute or subclnical mastitis) was unavailable [11]. As controls, ET3-1 and human TSS-associated strain MSA553, for which the complete genome sequence has also been recently determined (unpublished data), were used to establish statistical thresholds for gene presence and absence. SA isolates were grown at 37°C in 100 ml tryptic soy broth to mid-log phase (OD_600_ = 0.7−0.8). DNA was isolated by standard CTAB/organic extraction with the addition of lysostaphin (1 µg/ml) during cell lysis. Purified DNA was mechanically sheared into 350–700 bp fragments using a Hydroshear apparatus fitted with a custom short shearing assembly (GeneMachines). Sheared DNA (10 µg) was labeled overnight using 5 U Klenow enzyme and amino-allyl coupled dUTPs. Labeled DNA was washed and incubated with Cy3 and Cy5 fluorescent probes for 2 hours at room temperature (Amersham, Piscataway NJ). Meanwhile, array slides were incubated for 1 hr in preheated 42°C prehybridization buffer (25 ml formamide, 12.5 ml 20X SSC, 12 ml H_2_0, 300 µl 10% SDS and 500 µg BSA filtered with 2 µm syringe filter), rinsed thoroughly with 2 L H_2_0 and dried immediately by centrifugation. Labeled probes (12 µl each) were washed of excess dye, combined and added to the reaction mix: 9.8 µl formamide, 6.8 µl 20X SSC, 3.4 µl salmon sperm and 1 µl 10% SDS. Reactions were incubated at 99.9°C for 2 minutes in a thermal cycler, allowed to cool and applied to the prepared microarray. Arrays were incubated in a 42°C waterbath overnight, washed in successive dilutions of SSC wash buffer, dried and scanned using an Axon 4000B Scanner (Molecular Devices, Union City CA).

For each dual-colored probe hybridization experiment, ET3-1 DNA was used as the control sample. Each pair (ET3-1 vs. experimental strain) was repeated using a dye-swap format to obviate dye bias. With each probe on the array spotted in triplicate and each experiment repeated as a dye swap, 6 replicates per probe were generated. Human SA strain MSA553 was also hybridized against ET3-1 DNA to establish statistical thresholds for divergent and absent genes. Global and Lowess normalization procedures were not appropriate for analysis of these arrays because these methods both rely on the assumption that nearly all genes should have a balanced hybridization ratio. For each strain in these experiments, nearly one-quarter of the array probes were expected *not* to hybridize due to genomic variation. Thus, in order to establish an array normalization probe set, 1000 highly conserved genes were selected using *in silico* analysis of the nine completely sequenced SA genomes used to generate the array probes. A set of 750 conserved genes with consistently measurable and balanced intensity ratios in the control arrays were selected for standard global normalization using totaled mean channel intensity, and the resulting ratio was used to normalize the remaining array spots that has passed through filters (signal saturation and total intensity minus background, which were calculated by Axon GenePixPro software (Molecular Devices)). Genes were classified based on the ratio of experimental strain DNA signal intensity to ET3-1 DNA signal intensity (ratio≤0.35 = absent; 0.35<ratio<0.50 = variable; ratio≥0.50 = present). These thresholds were determined by comparing hybridization ratios with probe homology for the fully sequenced strains and by examining the ratios for three replicate hybridizations of MSA553 versus ET3-1 DNA.

For analysis of the human sequenced SA genomes, the homology of the microarray probes to the sequenced human isolates was determined using Smith-Waterman alignment and assigning (74). A scoring system accounting for gaps and single mismatches within the 70mer probe was developed in order to classify genes analogously to the microarray hybridization as absent, variable, or present. The microarray hybridization and Smith-Waterman alignments for strains ET3-1 and MSA553 were used to verify the accuracy of the *in silico* scoring system. Data from the DNA microarray hybridizations and the *in silico* hybridizations were compiled to generate Figure 6 using Spotfire (Somerville MA) for data visualization.

## Supporting Information

Table S1 ET3-1 Feature Table
(1.85 MB DOC)
