# Supplementary material for: Molecular Correlates of Host Specialization in Staphylococcus aureus
Source: PLoS One. 2007 Oct 31;2(10):e1120. doi: 10.1371/journal.pone.0001120 (PMC2040198; doi:10.1371/journal.pone.0001120)
Supplement: Table S1 — ET3-1 Feature Table (1.85 MB DOC) [file pone.0001120.s001.doc]

#### TABLE S1

#### ET3-1 Feature Table

| Amino acid biosynthesis | | |
| --- | --- | --- |
| Aromatic amino acid family | | |
| SAB0760 | aroD | 3-dehydroquinate dehydratase |
| SAB1220 | tyrA | prephenate dehydrogenase |
| SAB1222 | trpE | anthranilate synthase subunit |
| SAB1223 | trpG | anthranilate synthase component II |
| SAB1224 | trpD | anthranilate phosphoribosyltransferase |
| SAB1225 | trpC | indole-3-glycerol phosphate synthase |
| SAB1226 | trpF | N-5'-phosphoribosyl- anthranilate isomerase |
| SAB1227 | trpB | tryptophan synthase beta chain |
| SAB1228 | trpA | tryptophan synthase alpha chain |
| SAB1255 | lysA | diaminopimelate decarboxylase |
| SAB1328 | aroA | 3-phosphoshikimate 1-carboxyvinyltransferase |
| SAB1329 | aroB | 3-dehydroquinate synthase |
| SAB1330 | aroC | chorismate synthase |
| SAB1468 | aroE | shikimate 5-dehydrogenase |
| SAB1502 | dtd | D-tyrosyl-tRNA(Tyr) deacylase |
| SAB1597 | aroA | chorismate mutase |
| Aspartate family | |  |
| SAB0012 |  | homoserine-o-acetyltransferase |
| SAB0306 | metE1 | 5-methyltetrahydropteroyltriglutamate homocysteine methyltransferase |
| SAB0307 | metE2 | 5-methyltetrahydrofolate-homocysteine methyltransferase |
| SAB0308 |  | cystathionine beta-lyase |
| SAB0309 |  | cystathionine gamma-synthase |
| SAB0410 |  | cystathionine gamma-synthase |
| SAB1185 |  | aspartate kinase |
| SAB1186 |  | homoserine dehydrogenase |
| SAB1187 | thrC | threonine synthase |
| SAB1188 | thrB | homoserine kinase |
| SAB1248 | lysC | aspartate kinase II |
| SAB1249 | asd | aspartate semialdehyde dehydrogenase |
| SAB1250 | dapA | dihydrodipicolinate synthase |
| SAB1251 | dapB | dihydrodipicolinate reductase |
| SAB1252 | dapD | 2,3,4,5-tetrahydropyridine-2-carboxylate N-succinyltransferase |
| SAB1877 |  | succinyldiaminopimelate desuccinylase |
| Glutamate family | |  |
| SAB0122 | argB | acetylglutamate kinase |
| SAB0123 | argJ | arginine biosynthesis bifunctional protein |
| SAB0124 |  | N-acetyl-gamma-glutamyl-phosphate reductase (pseudogene) |
| SAB0420 |  | transcriptional activator of glutamate synthase operon |
| SAB0421 | gltA | glutamate synthase large subunit |
| SAB0422 | gltB | glutamate synthase small subunit |
| SAB0825 |  | NAD-specific glutamate dehydrogenase |
| SAB0828 | argH | argininosuccinate lyase |
| SAB0829 | argG | argininosuccinate synthase |
| SAB1033 |  | ornithine carbamoyltransferase |
| SAB1364 |  | pyrrolidone-5-carboxylate reductase |
| SAB1581 |  | soluble hydrogenase subunit |
| SAB1872 |  | aspartate transaminase |
| SAB2556 |  | ATP phosphoribosyltransferase regulatory subunit |
| Histidine family | |  |
| SAB0673 |  | histidinol-phosphate aminotransferase |
| SAB1946 | ilvA | threonine dehydratase |
| SAB2548 | hisIE | histidine biosynthesis bifunctional protein |
| SAB2549 | hisF | imidazole glycerol phosphate synthase subunit |
| SAB2550 | hisA | phosphoribosylformimino-5-aminoimidazole carboxamide ribotide isomerase |
| SAB2551 | hisH | imidazole glycerol phosphate synthase subunit |
| SAB2552 | hisB | imidazoleglycerol-phosphate dehydratase |
| SAB2554 | hisD | histidinol dehydrogenase |
| SAB2555 | hisG | ATP phosphoribosyltransferase |
| Pyruvate family | |  |
| SAB0505 | ilvE | branched-chain amino acid aminotransferase |
| SAB0798 | dltA | D-alanine-D-alanyl carrier protein ligase |
| SAB1938 |  | dihydroxyacid dehydratase |
| SAB1939 | ilvB | acetolactate synthase large subunit |
| SAB1940 | ilvH | acetolactate synthase small subunit |
| SAB1941 | ilvC | ketol-acid reductoisomerase |
| SAB1942 | leuA | 2-isopropylmalate synthase |
| SAB1943 | leuB | 3-isopropylmalate dehydrogenase |
| SAB1944 | leuC | 3-isopropylmalate dehydratase large subunit |
| SAB1945 | leuD | 3-isopropylmalate dehydratase small subunit |
| SAB1997 | glyA | serine hydroxymethyltransferase |
| Serine family | |  |
| SAB0055 |  | cysteine synthase A protein |
| SAB0409 |  | cysteine synthase |
| SAB0462 |  | cysteine synthase |
| SAB0479 | cysE | serine acetyltransferase |
| SAB0501 | kbl | 2-amino-3-ketobutyrate coenzyme A ligase |
| SAB0504 |  | L-threonine 3-dehydrogenase |
| SAB0796 |  | glycerate dehydrogenase |
| SAB1171 | glnR | glutamine synthetase transcription repressor |
| SAB1178 |  | low specificity threonine aldolase |
| SAB1582 | serA | D-3-phosphoglycerate dehydrogenase |
| SAB1880 | hemA | 5-aminolevulinic acid synthase |
| Biosynthesis of cofactors, prosthetic groups, and carriers | | |
| Biotin |  |  |
| SAB1320 | birA | bifunctional biotin operon-related protein and biotin-acetyl-CoA-carboxylase |
| SAB2304 | bioW | 6-carboxyhexanoate-CoA ligase |
| SAB2306 | bioB | biotin synthase |
| SAB2307 | bioA | adenosylmethionine-8-amino-7-oxononanoate aminotransferase |
| SAB2308 | bioD | dethiobiotin synthetase |
| Folic acid |  |  |
| SAB0463 | folP | dihydropteroate synthase |
| SAB0464 | folB | dihydroneopterin aldolase |
| SAB0465 | folK | 2-amino-4-hydroxy-6-hydroxymethyldihydropteridine pyrophosphokinase |
| SAB0662 |  | anthranilate/para-aminobenzoate synthase component II |
| SAB0663 |  | anthranilate/para-aminobenzoate synthase component I |
| SAB0664 |  | anthranilate/para-aminobenzoate synthase component I |
| SAB0930 | folD | bifunctional methylenetetrahydrofolate dehydrogenase and methenyltetrahydrofolate |
| SAB1281 | dfrB | trimethoprim-sensitive dihydrofolate reductase |
| SAB1523 | folC | folylpolyglutamate synthase |
| Glutathione and analogs | | |
| SAB1168 |  | glutathione peroxidase |
| SAB2495 |  | glutathione peroxidase |
| Heme, porphyrin, and cobalamin | | |
| SAB0636 |  | cobalamin synthesis-related protein |
| SAB0980 | ctaA | heme O-oxygenase |
| SAB0981 | ctaB | heme A IX farnesyltransferase |
| SAB1455 |  | oxygen-independent coproporphyrinogen oxidase III |
| SAB1527 | hemL | glutamate-1-semialdehyde 2,1-aminomutase |
| SAB1528 | hemB | delta-aminolevulinic acid dehydratase |
| SAB1529 | hemD | uroporphyrinogen III synthase |
| SAB1530 | hemC | porphobilinogen deaminase |
| SAB1531 |  | membrane uroporphyrinogen III methylase |
| SAB1532 | hemA | glutamyl-tRNA reductase |
| SAB1763 | hemY | protoporphyrinogen oxidase |
| SAB1764 | hemH | ferrochelatase |
| SAB1765 | hemE | uroporphyrinogen decarboxylase |
| SAB1797 |  | glutamate-1-semialdehyde aminotransferase |
| SAB1823 |  | cobyric acid synthase |
| SAB2278 |  | uroporphyrin-III C-methyltransferase |
| SAB2456 |  | cobalamin synthesis protein |
| Menaquinone and ubiquinone | | |
| SAB0573 |  | NADH dehydrogenase |
| SAB0908 |  | 1,4-dihydroxy-2-naphthoate octaprenyltransferase |
| SAB0909 |  | menaquinone-specific isochorismate synthase |
| SAB0910 |  | 2-oxoglutarate decarboxylase |
| SAB0912 | menB | naphthoate synthase |
| SAB1333 |  | menaquinone biosynthesis methyltransferase |
| SAB1394 | ispA | geranyltranstransferase |
| SAB1650 | menC | O-succinylbenzoic acid synthetase |
| SAB1651 | menE | O-succinylbenzoate-CoA ligase |
| Molybdopterin | |  |
| SAB2140 | moaA | molybdenum cofactor biosynthesis protein A |
| SAB2141 | mobA | molybdopterin-guanine dinucleotide biosynthesis |
| SAB2142 | moaD | molybdopterin converting factor small subunit |
| SAB2143 | moaE | molybdopterin converting factor small subunit |
| SAB2144 | mobB | molybdopterin-guanine dinucleotide biosynthesis protein |
| SAB2145 | moeA | molybdopterin biosynthesis protein |
| SAB2146 | moaC | molybdenum cofactor biosynthesis protein |
| SAB2147 | moaB | molybdenum cofactor biosynthesis protein |
| SAB2148 | moeB | molybdopterin biosynthesis protein |
| **Nicotinate and nicotinamide metabolism** | | |
| SAB0873 |  | inorganic polyphosphate/ATP-NAD kinase |
| SAB1466 |  | nicotinate-nucleotide adenylyltransferase |
| SAB1850 |  | nicotinate phosphoribosyltransferase |
| SAB2437 |  | phytoene dehydrogenase |
| Other |  |  |
| SAB0148 |  | gamma-glutamyltransferase |
| SAB0190 |  | 2-C-methyl-D-erythritol 4-phosphate cytidylyltransferase |
| SAB0194 |  | 2-C-methyl-D-erythritol 4-phosphate cytidylyltransferase |
| SAB0444 |  | 4-diphosphocytidyl-2-C-methyl-D-erythritol kinase |
| SAB0540 | mvaK1 | mevalonate kinase |
| SAB0541 | mvaD | mevalonate diphosphate decarboxylase |
| SAB0542 | mvaK2 | phosphomevalonate kinase |
| SAB0660 |  | 6-pyruvoyl tetrahydrobiopterin synthase |
| SAB0774 |  | ATP-binding ABC transporter protein |
| SAB0775 |  | conserved hypothetical protein |
| SAB0776 | csdB | cysteine desulfurase |
| SAB0791 |  | lipoic acid synthetase |
| SAB1122 | uppS | undecaprenyl pyrophosphate synthetase |
| SAB1498 |  | dinucleotide-utilizing enzyme |
| SAB2225 | fni | isopentenyl-diphosphate delta-isomerase |
| SAB2419 | mvaA | hydroxymethylglutaryl-CoA reductase |
| SAB2420 | mvaS | 3-hydroxy-3-methylglutaryl CoA synthase |
| SAB2472 | panB | 3-methyl-2-oxobutanoate hydroxymethyltransferase |
| Pantothenate and coenzyme A | | |
| SAB0149 |  | acyl carrier protein phosphodiesterase |
| SAB0659 |  | coenzyme PQQ synthesis homolog |
| SAB0989 | coaD | phosphopantetheine adenylyltransferase |
| SAB1075 |  | pantothenate metabolism flavoprotein |
| SAB1547 | coaE | dephospho-CoA kinase |
| SAB1849 |  | NAD synthetase |
| SAB1855 |  | bifunctional pyrazinamidase/nicotina-midase |
| SAB2325 |  | 2-dehydropantoate 2-reductase |
| SAB2470 | panD | aspartate 1-decarboxylase precursor |
| SAB2471 | panC | pantoate-beta-alanine ligase |
| Pyridoxine | | |
| SAB0469 |  | pyridoxine biosynthesis protein |
| SAB0470 |  | pyridoxine biosynthesis protein |
| SAB1628 | ribD | riboflavin specific deaminase |
| Riboflavin, FMN, and FAD | | |
| SAB1134 | ribC | bifunctional protein including riboflavin kinase and FAD synthase |
| SAB1625 | ribH | 6,7-dimethyl-8-ribityllumazine synthase |
| SAB1626 | ribA | riboflavin biosynthesis protein GTP cyclohydrolase II |
| SAB1627 | ribE | riboflavin synthase alpha chain |
| Thiamine |  |  |
| SAB0530 |  | phosphomethylpyrimidine kinase |
| SAB0917 |  | aromatic amino acid transferase |
| SAB1574 |  | thiamine biosynthesis protein |
| SAB1975 | thiE | thiamine-phosphate pyrophosphorylase |
| SAB1976 | thiM | hydroxyethylthiazole kinase |
| SAB1977 | truncated thiD | phosphomethylpyrimidine kinase |
| Cell envelope | |  |
| Biosynthesis and degradation of surface polysaccharides and lipopolysaccharides | | |
| SAB0067 |  | UDP-glucose 4-epimerase |
| SAB0068 | cap5M | capsular polysaccharide biosynthesis glycosyltransferase |
| SAB0069 | cap8H | capsular glycosyltransferase |
| SAB0070 | cps2H | capsular biosynthesis protein |
| SAB0071 |  | lipopolysaccharide flipase protein |
| SAB0090 | capA | capsular polysaccharide synthesis enzyme CapA |
| SAB0091 | capB | capsular polysaccharide synthesis enzyme CapB |
| SAB0092 | capC | capsular polysaccharide synthesis enzyme CapC |
| SAB0093 | capD | capsular polysaccharide synthesis enzyme CapD |
| SAB0094 | capE | capsular polysaccharide synthesis enzyme CapE |

| SAB0095  SAB0095 | capF | capsular polysaccharide  synthesis enzyme CapF |
| --- | --- | --- |
| SAB0096 | capG | capsular polysaccharide synthesis enzyme CapG |
| SAB0097 | cap8H | capsular polysaccharide synthesis enzyme CapH |
| SAB0098 | cap8I | capsular polysaccharide synthesis enzyme CapI |
| SAB0099 | cap8J | capsular polysaccharide synthesis enzyme CapJ |
| SAB0100 | cap8K | capsular polysaccharide synthesis enzyme CapK |
| SAB0101 | capL | capsular polysaccharide synthesis enzyme CapL |
| SAB0102 | capM | capsular polysaccharide synthesis enzyme CapM |
| SAB0103 | capN | capsular polysaccharide synthesis enzyme CapN |
| SAB0104 | capO | capsular polysaccharide synthesis enzyme CapO |
| SAB0105 | capP | capsular polysaccharide synthesis enzyme CapP |
| SAB0197 |  | glycosyl transferase |
| SAB0448 | gcaD | UDP-N-acetylglucosamine pyrophosphorylase |
| SAB0453 |  | membrane spanning protein |
| SAB0514 |  | poly(glycerol-phosphate) alpha-glucosyltransferase |
| SAB0515 |  | poly(glycerol-phosphate) alpha-glucosyltransferase |
| SAB0653 |  | bactoprenol glucosyl transferase or stress response regulator |
| SAB0799 | dltB | D-alanine lipoteichoic acid and wall teichoic acid esterification protein |
| SAB0800 | dltC | D-alanyl carrier protein |
| SAB0801 | dltD | D-alanine lipoteichoic acid and wall teichoic acid esterification secreted protein |
| SAB0901 |  | glycosyl transferase |
| SAB0919 | atl | autolysin |
| SAB1322 |  | glycosyltransferase |
| SAB1343 | ebpS | cell surface elastin binding protein |
| SAB1501 |  | N-acetylmuramoyl-L-alanine amidase |
| SAB1614 |  | polysaccharide transport protein |
| SAB1633 |  | exported protein |
| SAB1663 | hysA1 | hyaluronate lyase precursor |
| SAB1806 |  | monofunctional glycosyltransferase |
| SAB1995 | mnaA | UDP-GlcNAc 2-epimerase |
| SAB2134 | fmhB | methicillin resistance factor - peptidoglycan pentaglycine interpeptide biosynthetic protein |
| SAB2183 |  | autolysin E |
| SAB2374 | gtaB | UTP-glucose-1-phosphate uridyltransferase |
| SAB2523 |  | glycosyl transferase |
| SAB2537 |  | capsular polysaccharide biosynthesis protein |
| SAB2538 |  | capsular polysaccharide biosynthesis protein |
| SAB2539 |  | capsular polysaccharide biosynthesis protein |
| SAB2541 | icaA | intercellular adhesion protein A glucosaminyltransferase |
| SAB2542 | icaD | intercellular adhesion protein D |
| SAB2543 | icaB | intercellular adhesion protein B |
| SAB2544 | icaC | intercellular adhesion protein C |
| Cell Envelope  | **Biosynthesis of murein sacculus and peptidoglycan** | | --- | | | |
| SAB0192 |  | teichoic acid biosynthesis protein B |
| SAB0193 |  | teichoic acid biosynthesis protein F |
| SAB0196 |  | teichoic acid biosynthesis protein B |
| SAB0201 | lrgA | holin-like protein A |
| SAB0202 | lrgB | holin-like protein B |
| SAB0215 | lytM | peptidoglycan hydrolase |
| SAB0585 |  | membrane protein |
| SAB0586 | tagA | teichoic acid biosynthesis protein |
| SAB0587 | tagH | teichoic acid translocation ATP-binding protein |
| SAB0588 | tagG | teichoic acid translocation permease protein |
| SAB0589 | tagB | teichoic acid biosynthesis protein |
| SAB0590 |  | teichoic acid biosynthesis enzyme |
| SAB0591 | tagD | glycerol-3-phosphate cytidylyltransferase |
| SAB0592 | pbp4 | penicillin binding protein 4 |
| SAB0690 |  | UDP-N-acetylenolpyruvoylglucosamine reductase |
| SAB1014 | murI | glutamate racemase |
| SAB1045 | pbpA | penicillin-binding protein 1 |
| SAB1046 | mraY | phospho-N-acetylmuramoyl-pentapeptide-transferase |
| SAB1047 | murD | UDP-N-acetylmuramoylalanine-D-glutamate ligase |
| SAB1254 | alr | alanine racemase |
| SAB1273 | murG | UDP-N-acetylglucosamin--N-acetylmuramyl-(pentapeptide) pyrophosphoryl-undecaprenol N-acetylglucosamine transferase |
| SAB1314 | pbp2 | penicillin-binding protein 2 |
| SAB1424 | pbpF | penicillin-binding protein |
| SAB1494 |  | oxygenase |
| SAB1518 |  | truncated rod shape-determining protein |
| SAB1588 |  | penicillin-binding protein 1A |
| SAB1600 | murC | UDP-N-acetylmuramate-L-alanine ligase |
| SAB1824 |  | UDP-N-acetylmuramyl tripeptide synthase |
| SAB1955 | alr | alanine racemase 1 |
| SAB1966 |  | UDP-N-acetylmuramoylalanyl-D-glutamyl-2,6-diaminopimelate--D-alanyl-D-alanine ligase |
| SAB1967 |  | D-alanine-D-alanine ligase |
| SAB1984 | murA1 | UDP-N-acetylglucosamine 1-carboxyvinyltransferase 1 |
| SAB2291 | truncated fmhA | peptidoglycan synthesis protein (pseudogene) |
| Iron related metabolism | | |
| SAB0054 | sirA | iron-regulated lipoprotein |
| SAB0993 | isdA | iron-regulated cell wall-anchored protein |
| SAB0994 | isdB | cell surface transferrin-binding protein |
| SAB0995 | isdC | iron-regulated cell surface protein |
| SAB0996 | isdD | iron-regulated protein |
| SAB1590 |  | iron-regulated surface protein |
| SAB1591 |  | surface-anchored iron-regulated surface protein |
| Other |  |  |
| SAB0039 |  | lipoprotein (pseudogene) |
| SAB0073 |  | surface protein |
| SAB0084 |  | transport protein |
| SAB0113 |  | lipoprotein |
| SAB0147 |  | RGD-containing lipoprotein |
| SAB0159 |  | membrane protein |
| SAB0178 |  | membrane protein |
| SAB0210 |  | ribose transporter |
| SAB0217 |  | membrane protein |
| SAB0218 |  | membrane protein |
| SAB0219 |  | exported protein |
| SAB0224 |  | membrane protein |
| SAB0225 |  | membrane protein (pseudogene) |
| SAB0227 |  | membrane protein |
| SAB0241 |  | membrane protein |
| SAB0244 |  | exported protein |
| SAB0249 |  | membrane protein |
| SAB0256 |  | membrane protein |
| SAB0278 |  | membrane protein |
| SAB0290 |  | membrane protein |
| SAB0293 |  | lipoprotein |
| SAB0294 |  | membrane protein (pseudogene) |
| SAB0295 |  | membrane protein |
| SAB0300 |  | membrane protein |
| SAB0302 |  | membrane protein |
| SAB0303 |  | membrane protein |
| SAB0387 |  | exported protein |
| SAB0388 |  | exported protein |
| SAB0389 |  | lipoprotein |
| SAB0390 |  | lipoprotein |
| SAB0400 |  | membrane protein |
| SAB0405 |  | membrane protein |
| SAB0413 |  | membrane lipoprotein |
| SAB0415 |  | membrane protein |
| SAB0418 |  | membrane protein |
| SAB0419 |  | membrane protein |
| SAB0477 |  | membrane protein |
| SAB0524 |  | membrane protein |
| SAB0533 |  | membrane protein |
| SAB0535 |  | membrane protein |
| SAB0536 |  | membrane protein |
| SAB0546 |  | membrane protein |
| SAB0547 |  | membrane protein |
| SAB0566 |  | exported protein |
| SAB0567 |  | membrane protein |
| SAB0570 |  | membrane protein |
| SAB0595 |  | membrane protein |
| SAB0603 |  | membrane protein |
| SAB0615 |  | membrane protein |
| SAB0623 |  | membrane protein |
| SAB0624 |  | membrane protein |
| SAB0626 |  | lipoprotein |
| SAB0638 |  | membrane protein |
| SAB0641 |  | membrane protein |
| SAB0642 |  | membrane protein |
| SAB0645 |  | exported protein |
| SAB0656 |  | membrane protein |
| SAB0657 |  | lipoprotein |
| SAB0658 |  | membrane protein |
| SAB0665 |  | membrane protein |
| SAB0679 |  | membrane protein |
| SAB0692 |  | lipoprotein |
| SAB0696 |  | membrane protein |
| SAB0697 |  | membrane protein |
| SAB0698 |  | membrane protein |
| SAB0700 |  | membrane protein |
| SAB0726 |  | exported protein |
| SAB0733 |  | membrane protein |
| SAB0741 |  | lipoprotein |
| SAB0742 |  | membrane protein |
| SAB0752 |  | exported protein |
| SAB0753 |  | membrane protein |
| SAB0754 |  | membrane protein |
| SAB0773 |  | membrane protein |
| SAB0785 |  | membrane protein |
| SAB0789 |  | membrane protein |
| SAB0797 |  | membrane protein |
| SAB0832 |  | membrane protein |
| SAB0838 |  | membrane protein |
| SAB0842 |  | membrane-embedded acyltransferase (pseudogene) |
| SAB0851 |  | membrane protein |
| SAB0878 |  | membrane protein |
| SAB0882 |  | membrane protein |
| SAB0894 |  | membrane protein |
| SAB0895 |  | exported protein (pseudogene) |
| SAB0896 |  | membrane protein |
| SAB0897 |  | exported protein |
| SAB0899 |  | membrane protein |
| SAB0906 |  | membrane protein |
| SAB0922 |  | exported protein |
| SAB0928 |  | exported protein |
| SAB0944 |  | membrane protein |
| SAB0945 |  | membrane protein |
| SAB0958 |  | lipoprotein |
| SAB0969 |  | membrane protein |
| SAB0970 |  | membrane protein |
| SAB0982 |  | membrane protein |
| SAB0983 |  | membrane protein |
| SAB0985 |  | exported protein |
| SAB0999 | srtB | NPQTN specific sortase B |
| SAB1006 |  | membrane protein |
| SAB1018 |  | exported protein |
| SAB1020 |  | membrane protein |
| SAB1023 |  | membrane protein |
| SAB1035 |  | membrane protein |
| SAB1036 |  | membrane protein |
| SAB1054 |  | membrane protein |
| SAB1078 |  | membrane protein |
| SAB1106 |  | membrane protein (pseudogene) |
| SAB1143 |  | membrane protein |
| SAB1156 |  | membrane protein |
| SAB1181 |  | membrane protein |
| SAB1201 |  | exported protein |
| SAB1202 |  | membrane protein |
| SAB1210 |  | membrane protein |
| SAB1215 |  | membrane protein |
| SAB1216 | mprF | membrane protein |
| SAB1239 |  | membrane protein |
| SAB1261 |  | membrane protein |
| SAB1272 |  | membrane protein |
| SAB1287 |  | membrane protein |
| SAB1288 |  | cell wall enzyme |
| SAB1307 |  | membrane protein |
| SAB1324 |  | membrane protein |
| SAB1325 |  | membrane protein |
| SAB1347 |  | membrane protein |
| SAB1348 |  | lipoprotein |
| SAB1349 |  | lipoprotein |
| SAB1386 |  | membrane protein |
| SAB1403 |  | lipoprotein |
| SAB1404 |  | exported protein |
| SAB1406 |  | membrane protein |
| SAB1412 |  | exported protein |
| SAB1413 |  | exported protein |
| SAB1421 |  | membrane protein |
| SAB1445 |  | exported protein |
| SAB1446 |  | membrane protein |
| SAB1472 |  | membrane protein |
| SAB1475 |  | membrane protein |
| SAB1507 |  | exported protein |
| SAB1517 |  | membrane protein |
| SAB1520 |  | membrane protein |
| SAB1526 |  | membrane protein |
| SAB1550 |  | membrane protein |
| SAB1572 |  | thiol peroxidase |
| SAB1573 |  | membrane protein |
| SAB1606 |  | membrane protein |
| SAB1615 |  | exported protein |
| SAB1637 |  | membrane protein |
| SAB1638 |  | membrane protein (pseudogene) |
| SAB1640 |  | membrane protein |
| SAB1641 |  | intergral membrane protein |
| SAB1642 |  | membrane protein |
| SAB1644 |  | exported protein |
| SAB1647 |  | conserved hypothetical protein |
| SAB1648 |  | conserved hypothetical protein |
| SAB1649 |  | conserved hypothetical protein |
| SAB1652 |  | lipoprotein (pseudogene) |
| SAB1654 |  | membrane protein |
| SAB1655 |  | lipoprotein |
| SAB1664 |  | exported protein |
| SAB1690 |  | membrane protein |
| SAB1691 |  | exported protein |
| SAB1770 |  | exported protein |
| SAB1773 |  | membrane protein |
| SAB1779 |  | membrane protein |
| SAB1785 |  | membrane protein |
| SAB1788 |  | membrane protein |
| SAB1793 |  | membrane protein |
| SAB1798 |  | membrane protein |
| SAB1802 |  | membrane protein |
| SAB1803 |  | membrane protein |
| SAB1804 |  | membrane protein |
| SAB1814 |  | exported protein |
| SAB1815 |  | membrane protein |
| SAB1818 |  | membrane protein |
| SAB1821 |  | membrane protein |
| SAB1828 |  | membrane protein |
| SAB1838 |  | lipoprotein |
| SAB1848 |  | membrane protein |
| SAB1860 |  | exported protein |
| SAB1862 |  | exported protein |
| SAB1863 |  | membrane protein |
| SAB1864 |  | membrane protein |
| SAB1866 |  | membrane protein |
| SAB1868 |  | membrane protein |
| SAB1871 |  | membrane protein |
| SAB1873 |  | cell surface protein |
| SAB1915 |  | membrane protein |
| SAB1929 |  | membrane protein |
| SAB1932 |  | membrane protein |
| SAB1957 |  | membrane protein |
| SAB1958 |  | membrane protein |
| SAB1968 |  | membrane protein |
| SAB1974 |  | membrane-embedded lipoprotein precursor |
| SAB1980 |  | exported protein |
| SAB1985 |  | membrane protein |
| SAB2019 |  | membrane protein |
| SAB2026 |  | membrane protein |
| SAB2042 |  | exported protein |
| SAB2043 |  | membrane protein |
| SAB2053 |  | membrane protein |
| SAB2055 |  | membrane protein |
| SAB2064 |  | membrane protein |
| SAB2085 |  | exported protein |
| SAB2124 |  | membrane protein |
| SAB2130 |  | membrane protein |
| SAB2132 |  | membrane protein |
| SAB2135 |  | membrane protein |
| SAB2175 |  | membrane protein |
| SAB2190 |  | membrane protein |
| SAB2194 |  | membrane protein |
| SAB2195 |  | membrane protein |
| SAB2196 |  | membrane protein |
| SAB2204 |  | membrane protein |
| SAB2212 |  | membrane protein |
| SAB2216 |  | membrane protein |
| SAB2218 |  | membrane protein |
| SAB2220 |  | membrane protein |
| SAB2230 |  | membrane protein |
| SAB2237 |  | membrane protein |
| SAB2243 |  | membrane protein |
| SAB2248 |  | putative lipoprotein |
| SAB2253 |  | membrane protein |
| SAB2254 |  | membrane protein |
| SAB2262 |  | exported protein |
| SAB2264 |  | exported protein |
| SAB2284 |  | membrane protein |
| SAB2286 |  | zinc-binding lipoprotein |
| SAB2289 |  | lipoprotein |
| SAB2290 |  | lipoprotein |
| SAB2303 |  | membrane protein |
| SAB2313 |  | membrane protein |
| SAB2315 |  | membrane protein |
| SAB2322 |  | membrane permease |
| SAB2342 |  | lipoprotein |
| SAB2344 |  | membrane protein |
| SAB2358 |  | membrane protein |
| SAB2372 |  | exported protein |
| SAB2373 |  | membrane protein |
| SAB2383 |  | membrane protein |
| SAB2389 |  | membrane-spanning protein |
| SAB2391 |  | membrane protein |
| SAB2402 | srtA | sortase |
| SAB2409 |  | exported protein |
| SAB2414 |  | membrane protein |
| SAB2415 |  | membrane protein (pseudogene) |
| SAB2441 |  | membrane-embedded acyltransferase |
| SAB2457 |  | membrane protein |
| SAB2465 |  | membrane protein |
| SAB2478 |  | membrane protein |
| SAB2481 |  | membrane protein |
| SAB2497 |  | membrane protein |
| SAB2501 |  | membrane protein |
| SAB2514 |  | exported protein |
| SAB2518 |  | conserved membrane protein |
| SAB2519 |  | exported protein |
| SAB2521 |  | surface anchored protein |
| SAB2528 |  | membrane protein |
| SAB2531 |  | lipoprotein |
| SAB2557 |  | lipoprotein |
| SAB2561 |  | membrane protein |
| SAB2572 |  | membrane protein |
| SAB2577 |  | membrane protein |
| SAB2580 |  | membrane protein |
| SAB2582 |  | membrane protein |
| SAB2583 |  | membrane protein |
| SAB2584 |  | membrane protein |
| SAB1460 |  | membrane protein (pseudogene) |
| SAB0011 |  | membrane protein |
| SAB0121 |  | integral membrane protein |
| SAB2069 |  | conserved exported hypothetical protein |
| Cellular processes | | |
| Adaptations to atypical conditions | | |
| SAB0749 | cspC | cold-shock protein C |
| SAB0872 |  | GTP pyrophosphokinase |
| SAB1089 |  | alkaline-shock protein |
| SAB1258 | cspA | cold shock protein |
| SAB1443 |  | phosphate starvation-induced protein |
| SAB1503 | relA | GTP pyrophosphokinase |
| SAB1569 |  | stress response protein |
| SAB1580 |  | osmotic stress-related protein |
| SAB1599 |  | general stress response protein |
| SAB1949 | sigB | RNA polymerase sigma factor B |
| SAB2023 |  | general stress protein 20U |
| SAB2063 | asp23 | alkaline shock protein |
| SAB2486 | cudB | choline dehydrogenase |
| SAB2487 | cudA | glycine betaine aldehyde dehydrogenase |
| SAB2581 | cspB | cold shock protein |
| Cell adhesion | |  |
| SAB0512 | truncated sdrC | Ser-Asp rich fibrinogen-binding/bone sialoprotein-binding protein (pseudogene) |
| SAB0513 |  | Ser-Asp rich fibrinogen-binding/bone sialoprotein-binding protein |
| SAB0744 | clfA | truncated clumping factor |
| SAB0745 |  | secreted von Willebrand factor-binding protein precursor |
| SAB0746 |  | extracellular matrix and plasma binding protein precursor |
| SAB0747 |  | truncated secreted von Willebrand factor-binding protein homolog |
| SAB1021 | fib | fibrinogen-binding protein |
| SAB1022 |  | fibrinogen-binding protein precursor |
| SAB1072 |  | fibronectin/fibrinogen-binding protein |
| SAB1289 |  | truncated cell surface fibronectin-binding protein |
| SAB1290 |  | truncated cell surface fibronectin-binding protein |
| SAB1291 |  | truncated cell surface fibronectin-binding protein |
| SAB1292 |  | truncated cell surface fibronectin-binding protein |
| SAB1293 |  | truncated cell surface fibronectin-binding protein |
| SAB1300 |  | truncated cell surface fibronectin-binding protein |
| SAB1916 |  | membrane anchored Ser-Asp rich fibrinogen-binding protein |
| SAB2375 | fnbA | fibronectin-binding protein |
| SAB2505 | clfB | clumping factor B |
| SAB2529 |  | surface expressed Ser-Thr rich repeat protein (pseudogene) |

| Cell division | |  |
| --- | --- | --- |
| SAB0198 | scdA | cell division and morphogenesis-related protein |
| SAB0460 | ftsH | cell division protein |
| SAB0724 |  | cell division inhibitor |
| SAB0978 |  | cell division protein |
| SAB1044 |  | cell division protein |
| SAB1048 |  | cell division protein |
| SAB1049 | ftsA | cell division protein |
| SAB1050 | ftsZ | cell division protein |
| SAB1056 |  | cell division-initiation protein |
| SAB1098 |  | chromosome segregation SMC protein |
| SAB1113 | gid | glucose inhibited division protein A |
| SAB1138 |  | DNA translocase SpoIII family |
| SAB1439 | era | GTP-binding protein |
| SAB1769 |  | cell cycle regulation protein |
| SAB2586 | gidB | glucose inhibited division protein B |
| SAB2587 | gidA | glucose inhibited division protein A |
| Detoxification | |  |
| SAB0072 | sodM | superoxide dismutase [Mn/Fe] |
| SAB0330 | ahpF | alkyl hydroperoxide reductase subunit F |
| SAB0331 | ahpC | alkyl hydroperoxide reductase subunit C |
| SAB0735 |  | carboxylesterase precursor |
| SAB0887 |  | toxic anion resistance membrane protein |
| SAB1192 |  | catalase |
| SAB1425 | sodA | superoxide dismutase |
| SAB1630 | arsR | arsenical resistance operon repressor |
| SAB1631 | arsB | arsenic efflux pump protein |
| SAB1632 |  | arsenate reductase |
| SAB2588 |  | tRNA modification GTPase |
| DNA transformation | | |
| SAB0702 | comFA | late competence operon protein FA |
| SAB0703 | comFC | late competence operon protein FC |
| SAB0865 |  | genetic competence repressor |
| SAB0866 |  | competence-related protein |
| SAB1111 | smf | DNA processing protein |
| SAB1414 |  | exogenous DNA-binding protein |
| SAB1416 |  | late competence protein |
| SAB1461 |  | late competence deaminase protein |
| SAB1462 |  | competence-related membrane protein |
| Pathogenesis | |  |
| SAB0045 |  | myosin-crossreactive streptococcal antigen-like protein |
| SAB0050 | truncated spa | immunoglobulin G binding protein A precusor (pseudogene) |
| SAB0169 |  | staphylocoagulase precursor |
| SAB0222 |  | autolysin precursor (pseudogene) |
| SAB0414 |  | autolysin |
| SAB0614 |  | secretory antigen SsaA-like protein |
| SAB0654 | saeS | histidine protein kinase |
| SAB0655 | saeR | response regulator |
| SAB1669 | splF | serine proteinase |
| SAB1670 | splE | serine proteinase |
| SAB1671 | splC | serine proteinase |
| SAB1672 |  | serine proteinase |
| SAB1673 |  | serine proteinase (pseudogene) |
| SAB1766 | traP | signal transduction protein |
| SAB1845 |  | staphopain cysteine proteinase |
| SAB2082 | hysA2 | hyaluronate lyase |
| SAB2172 |  | secretory antigen staphyloxanthin precursor |
| SAB2176 |  | secretory antigen staphyloxanthin precursor |
| SAB2298 | sbi | immunoglobulin G-binding protein |
| SAB2299 |  | hypothetical protein |
| SAB2300 | hlgA | gamma-hemolysin component A precursor |
| SAB2301 | hlgC | gamma-hemolysin component C |
| SAB2302 | hlgB | gamma-hemolysin component B |
| SAB2418 |  | secretory antigen SsaA homolog |
| SAB2439 | ssaA | secretory antigen precursor |
| SAB2443 | isaA | immunodominant antigen A |
| SAB2513 | isaB | immunodominant antigen B |
| Resistance mechanisms | | |
| SAB0043 |  | antibiotic resistance-related transmembrane efflux protein |
| SAB0058 |  | membrane transport protein |
| SAB0076 |  | membrane transport protein, tetracycline resistance |
| SAB0118 |  | integral membrane transport protein |
| SAB0214 |  | penicillin-related choloylglycine hydrolase |
| SAB0442 |  | dimethyladenosine transferase |
| SAB0632 | bacA | undecaprenol kinase bacitracin resistance protein |
| SAB0644 | norA | quinolone resistance protein |
| SAB0661 |  | aluminum resistance protein |
| SAB0675 |  | multidrug resistance protein |
| SAB0884 | murE | UDP-N-acetylmuramoylalanyl-D-glutamate 2,6-diaminopimelate ligase |
| SAB0923 | fmt | autolysis and methicillin resistant-related protein |
| SAB1170 |  | aluminum resistance protein |
| SAB1172 | glnA | glutamine synthetase |
| SAB1229 | femA | factor essential for expression of methicillin resistance |
| SAB1230 | femB | methicillin resistance factor protein |
| SAB1262 |  | toxic ion resistance protein |
| SAB1616 |  | surface-anchored fmtB-like protein (pseudogene) |
| SAB1619 |  | multidrug resistance protein B |
| SAB1892 |  | multidrug resistance protein |
| SAB2008 | murA2 | UDP-N-acetylglucosamine 1-carboxyvinyltransferase 2 |
| SAB2040 | truncated fmtB | truncated methicillin resistance-related surface protein |
| SAB2041 |  | phosphoglucosamine-mutase |
| SAB2047 |  | multidrug resistance protein |
| SAB2048 |  | multidrug resistance protein |
| SAB2050 |  | drug resistance-involved transporter |
| SAB2133 |  | acriflavin resistance transport protein |
| SAB2231 |  | multidrug resistance transporter |
| SAB2232 |  | exported multidrug resistance protein |
| SAB2234 | tcaB | bicyclomycin and teicoplanin resistance protein |
| SAB2235 | tcaA | bicyclomycin and teicoplanin resistance protein |
| SAB2236 | tcaR | bicyclomycin resistance transcriptional regulator |
| SAB2295 |  | multidrug resistanc-related transport system protein |
| SAB2323 |  | drug resistance-involved membrane protein |
| SAB2326 |  | transmembrane efflux protein |
| SAB2345 |  | antibiotic resistance transport protein |
| SAB2429 |  | galactoside-O-acetyltransferase |
| SAB2564 | drp35 | antibiotic-induced protein |
| Sporulation and germination | | |
| SAB0228 |  | DNA segregation ATPase and related protein |
| SAB0447 | spoVG | stage V sporulation protein G |
| SAB1513 |  | Spo0B-associated GTP-binding protein |
| SAB1601 |  | SpoIIIE family cell division protein |
| Toxin production | | |
| SAB0026 |  | enterotoxin protein |
| SAB0360 | tsst-1 | toxic shock syndrome toxin-1 |
| SAB0363 | sec-bov | staphylococcal enterotoxin C-bovine |
| SAB0364 | sel | staphylococcal enterotoxin L |
| SAB0376 | set11 | staphylococcal exotoxin 11 |
| SAB0377 | set10 | staphylococcal exotoxin 10 |
| SAB0378 | set9 | staphylococcal exotoxin 9 |
| SAB0379 | set7 | staphylococcal exotoxin 7 |
| SAB0380 | set5 | staphylococcal exotoxin 5 |
| SAB0381 | set4 | staphylococcal exotoxin 4 |
| SAB0382 | set3 | staphylococcal exotoxin 3 |
| SAB0383 | set2 | staphylococcal exotoxin 2 |
| SAB0386 | set1 | staphylococcal exotoxin 1 |
| SAB0782 |  | leukocidin chain lukM precursor |
| SAB0783 |  | Panton-Valentine leukocidin LukF-PV chain precursor |
| SAB0786 |  | hemolysin |
| SAB1027 | hla | alpha-hemolysin precursor |
| SAB1030 |  | exotoxin |
| SAB1031 |  | exotoxin |
| SAB1032 |  | exotoxin |
| SAB1037 |  | exfoliative toxin |
| SAB1373 |  | streptolysin-associated protein SagD homolog |
| SAB1376 |  | streptolysin S-associated protein SagB homolog |
| SAB1473 |  | staphylococcal enterotoxin (pseudogene) |
| SAB1686 | lukD | leukotoxin D subunit |
| SAB1687 | lukE | leukotoxin E subunit |
| SAB1696 | seg | enterotoxin G |
| SAB1697 | sen | enterotoxin N |
| SAB1698 | sec-variant | enterotoxin type C variant |
| SAB1699 | sei | enterotoxin I |
| SAB1700 | sem-truncated | enterotoxin M (pseudogene) |
| SAB1701 | seo | enterotoxin O |
| SAB1874 |  | beta-hemolysin |
| SAB1875 |  | leukocidin F subunit |
| SAB1876 |  | leukocidin S subunit |
| Central intermediary metabolism | | |
| Amino sugars | |  |
| SAB0252 | nanA | N-acetylneuraminate lyase subunit |
| SAB0255 |  | N-acetylmannosamine-6-phosphate 2-epimerase |
| SAB0500 | amaA | N-acyl-L-amino acid amidohydrolase |
| SAB0519 |  | glucosamine-6-phosphate deaminase |
| SAB0650 | nagA | N-acetylglucosamine-6-phosphate deacetylase |
| SAB2034 | glmS | glucosamine-fructose-6-phosphate aminotransferase |
| Iron and sulfur metabolism | | |
| SAB0544 |  | mercury(II) reductase |
| SAB1000 |  | cytosolic heme degrading enzyme |
| SAB1493 |  | iron-sulfur cofactor synthesis protein |
| SAB1856 | ppaC | manganese-dependent inorganic pyrophosphatase |
| SAB2494 | cysJ | sulfite reductase flavoprotein |
| Nitrogen metabolism | | |
| SAB0777 |  | nitrogen fixation protein |
| SAB0802 |  | nitrogen fixation protein |
| SAB1265 |  | nitric oxide reductase protein |
| SAB1851 |  | nitric oxide synthase |
| SAB2160 | ureA | urease gamma subunit |
| SAB2161 | ureB | urease beta subunit |
| SAB2162 | ureC | urease alpha subunit |
| SAB2163 | ureE | urease accessory protein |
| SAB2164 | ureF | urease accessory protein |
| SAB2165 | ureG | urease accessory protein |
| SAB2166 | ureD | urease accessory protein |
| SAB2273 | narI | nitrate reductase gamma chain |
| SAB2274 | narJ | nitrate reductase delta chain |
| SAB2275 | narH | nitrate reductase beta chain |
| SAB2276 | narG | nitrate reductase alpha chain |
| SAB2279 | nasE | assimilatory nitrite reductase |
| SAB2280 | nasD | nitrite reductase |
| One-carbon metabolism | | |
| SAB1592 | fhs | formyltetrahydrofolate synthetase |
| SAB1645 | metK | S-adenosylmethionine synthetase |
| Other |  |  |
| SAB0061 |  | aldolase |
| SAB0128 |  | pyruvate decarboxylase |
| SAB0795 |  | 4-nitrophenylphosphatase |
| SAB1332 |  | heptaprenyl diphosphate synthase component II |
| SAB1570 | ackA | acetate kinase |
| SAB2178 |  | dehydrogenase |
| SAB2398 | ddh | D-specific D-2-hydroxyacid dehydrogenase |
| SAB2432 |  | D-specific D-2-hydroxyacid dehydrogenase |
| SAB2434 | crtN | squalene synthase |
| SAB2435 | crtM | squalene desaturase |
| DNA metabolism | | |
| **Degradation of DNA** | | |
| SAB0748 | nuc | staphylococcal thermonuclease precursor |
| SAB1182 | nucI | thermonuclease |
| SAB1305 |  | 5'-3' exonuclease |
| SAB1395 |  | exodeoxyribonuclease VII small subunit |
| SAB1396 |  | exodeoxyribonuclease VII large subunit |
| DNA replication, recombination, and repair | | |
| SAB0001 | dnaA | chromosomal replicator initiator protein |
| SAB0002 | dnaN | DNA polymerase III beta chain |
| SAB0004 | recF | DNA replication and repair protein |
| SAB0005 | gyrB | DNA gyrase subunit B |
| SAB0006 | gyrA | DNA gyrase subunit A |
| SAB0016 | dnaC | replicative DNA helicase |
| SAB0317 | ssb | single-strand DNA-binding protein |
| SAB0320 |  | fragment of truncated integrase |
| SAB0427 | dnaX | DNA polymerase III gamma and tau subunits |
| SAB0429 | recR | recombination protein |
| SAB0433 | holB | DNA polymerase III delta prime subunit |
| SAB0452 |  | transcription-repair coupling factor |
| SAB0476 | radA | DNA repair protein |
| SAB0531 | ung | uracil-DNA glycosylase |
| SAB0561 |  | DNA repair endonuclease |
| SAB0640 |  | DNA photolyase |
| SAB0670 |  | ATP-dependent DNA helicase |
| SAB0711 | uvrB | exinuclease ABC subunit B |
| SAB0712 | uvrA | exinuclease ABC subunit A |
| SAB0835 |  | ATP-dependent nuclease subunit B |
| SAB0836 |  | ATP-dependent nuclease subunit A |
| SAB1004 |  | ribonuclease HIII |
| SAB1007 |  | DNA-dependent DNA polymerase beta chain |
| SAB1008 |  | DNA mismatch repair protein |
| SAB1010 | uvrC | excinuclease ABC subunit C |
| SAB1076 | priA | primosomal protein |
| SAB1091 | recG | ATP-dependent DNA helicase |
| SAB1112 | topA | DNA topoisomerase I |
| SAB1126 | polC | DNA polymerase III |
| SAB1146 | cinA | competence-damage inducible protein |
| SAB1147 | recA | recombinase A protein |
| SAB1157 | mutS | DNA mismatch repair protein |
| SAB1158 | mutL | DNA mismatch repair protein (pseudogene) |
| SAB1203 |  | exonuclease |
| SAB1204 |  | exonuclease |
| SAB1211 | grlB | topoisomerase IV subunit B |
| SAB1212 | grlA | topoisomerase IV subunit A |
| SAB1219 |  | DNA damage repair protein |
| SAB1313 | recU | recombination protein U |
| SAB1316 |  | endonuclease III-like protein |
| SAB1317 |  | DNA replication initiation protein |
| SAB1319 | dinG | ATP-dependent DNA helicase |
| SAB1335 | hup | DNA-binding protein II |
| SAB1344 |  | ATP-dependent DNA helicase |
| SAB1358 | xerD | site-specific recombinase |
| SAB1392 |  | DNA repair protein |
| SAB1401 | efp | elongation factor P |
| SAB1429 |  | endonuclease IV |
| SAB1434 | dnaG | DNA primase |
| SAB1438 |  | recombination protein |
| SAB1487 |  | Holliday junction resolvase |
| SAB1505 |  | single-strand DNA-specific exonuclease |
| SAB1510 | ruvB | Holliday junction DNA helicase |
| SAB1511 | ruvA | Holliday junction DNA helicase |
| SAB1521 | radC | DNA repair protein homolog |
| SAB1525 | tag | DNA-3-methyladenine glycosidase |
| SAB1543 | dnaI | primosomal protein |
| SAB1544 | dnaB | chromosome replication initiation and membrane attachment protein |
| SAB1548 | mutM | formamidopyrimidine DNA glycosylase |
| SAB1549 | polA | DNA polymerase I |
| SAB1561 | dnaE | DNA polymerase III alpha subunit |
| SAB1741 | dnaB | replicative DNA helicase |
| SAB1801 |  | A/G-specific adenine glycosylase DNA repair protein |
| SAB1826 |  | DNA polymerase III alpha chain |
| SAB1827 |  | DNA polymerase IV |
| SAB1839 | lig | DNA ligase |
| SAB1840 | pcrA | ATP-dependent DNA helicase |
| SAB1841 |  | PcrB family replication protein |
| SAB1981 |  | single strand DNA binding protein |
| SAB2126 |  | DNA topoisomerase III (pseudogene) |
| SAB2222 |  | 3-methyladenine DNA glycosylase |
| SAB2223 |  | 3-methyladenine DNA glycosylase |
| SAB2422 | adaB | methylated-DNA-protein-cysteine S-methyltransferase |
| Other |  |  |
| SAB1512 |  | DNA-binding protein |
| SAB1563 |  | DNA-binding protein |
| SAB1635 |  | DNA-binding protein |
| SAB1842 |  | DNA-binding protein |
| **Restriction/modification** | | |
| SAB0135 |  | type I restriction enzyme restriction chain |
| SAB0384 | hsdM | type I site-specific deoxyribonuclease |
| SAB0385 | truncated hsdS | type I site-specific deoxyribonuclease subunit (pseudogene) |
| SAB1667 |  | specificity determinant (pseudogene) |
| SAB1668 | hsdM | type I restriction-modification system M subunit |
| Energy metabolism | | |
| Amino acids and amines | | |
| SAB0008 | hutH | histidine ammonia lyase |
| SAB0056 |  | ornithine cyclodeaminase protein |
| SAB0125 | argD | acetylornithine aminotransferase |
| SAB0274 |  | glycine cleavage system H protein |
| SAB0764 | gcvH | glycine cleavage system H protein |
| SAB0824 | rocD | ornithine aminotransferase |
| SAB0837 |  | fumarylacetoacetate hydrolase family protein |
| SAB1034 |  | carbamate kinase |
| SAB1303 | tdcB | threonine dehydratase |
| SAB1304 | ald | alanine dehydrogenase |
| SAB1341 |  | L-asparaginase |
| SAB1388 |  | branched-chain alpha-keto acid dehydrogenase E2 |
| SAB1389 |  | 2-oxoisovalerate dehydrogenase beta subunit |
| SAB1390 |  | 2-oxoisovalerate dehydrogenase alpha subunit |
| SAB1391 |  | dihydrolipoamide dehydrogenase |
| SAB1407 |  | glycine dehydrogenase subunit 2 |
| SAB1408 |  | glycine dehydrogenase subunit 1 |
| SAB1409 | gcvT | aminomethyltransferase |
| SAB1568 | ald | alanine dehydrogenase |
| SAB1610 | dat | D-alanine aminotransferase |
| SAB1624 |  | proline oxidase |
| SAB2044 | rocF | arginase |
| SAB2207 | hutI | imidazolonepropionase |
| SAB2208 | hutU | urocanate hydratase |
| SAB2211 | hutG | formiminoglutamase |
| SAB2404 |  | L-serine dehydratase alpha subunit |
| SAB2405 |  | L-serine dehydratase beta subunit |
| SAB2428 | putA | 1-pyrroline-5-carboxylate dehydrogenase |
| SAB2507 | arcC | carbamate kinase |
| SAB2509 | arcB | catabolic ornithine carbamoyltransferase |
| SAB2510 | arcA | arginine deiminase |
| **Anaerobic** | | |
| SAB0117 |  | NAD-dependent formate dehydrogenase |
| SAB0164 | pflB | formate acetyltransferase |
| SAB0165 | pflA | formate acetyltransferase activating enzyme |
| SAB0180 |  | L-lactate dehydrogenase 1 |
| SAB1151 |  | pyruvate ferredoxin oxidoreductase alpha chain |
| SAB1152 |  | pyruvate ferredoxin oxidoreductase beta chain |
| SAB2186 |  | formate dehydrogenase alpha subunit |
| SAB2475 | ldh | L-lactate dehydrogenase |
| ATP-proton motive force interconversion | | |
| SAB1986 | atpC | ATP synthase epsilon chain |
| SAB1987 | atpD | ATP synthase beta chain |
| SAB1988 | atpG | ATP synthase gamma chain |
| SAB1989 | atpA | ATP synthase alpha chain |
| SAB1990 | atpH | ATP synthase delta chain |
| SAB1991 | atpF | ATP synthase B chain |
| SAB1992 | atpE | ATP synthase C chain |
| SAB1993 | atpB | ATP synthase A chain |
| Biosynthesis and degradation of polysaccharides | | |
| SAB0205 | bglA | 6-phospho-beta-glucosidase |
| SAB0424 |  | alpha-glucosidase |
| SAB1368 | malA | alpha-D-1,4-glucosidase |
| SAB1925 | scrB | sucrose-6-phosphate hydrolase |
| SAB2070 | lacG | 6-phospho-beta-galactosidase |
| SAB2073 | lacD | tagatose 1,6-diphosphate aldolase |
| SAB2074 | lacC | tagatose-6-phosphate kinase |
| SAB2075 | lacB | galactose-6-phosphate isomerase subunit |
| SAB2076 | lacA | galactose-6-phosphate isomerase subunit |
| Electron transport | | |
| SAB0032 |  | sulfide-quinone reductase |
| SAB0156 |  | NADH-dependent dehydrogenase |
| SAB0157 |  | NADH-dependent dehydrogenase |
| SAB0289 |  | NADH-dependent FMN reductase |
| SAB0332 |  | nitro/flavin reductase |
| SAB0402 |  | NADH dehydrogenase subunit 5 |
| SAB0717 | trxB | thioredoxin reductase |
| SAB0762 |  | thioredoxin |
| SAB0804 |  | NADH dehydrogenase |
| SAB0807 |  | NADH dehydrogenase |
| SAB0839 |  | coenzyme A disulfide reductase |
| SAB0924 | qoxD | quinol oxidase polypeptide IV |
| SAB0925 | qoxC | quinol oxidase polypeptide III |
| SAB0926 | qoxB | quinol oxidase polypeptide I |
| SAB0927 | qoxA | quinol oxidase polypeptide II |
| SAB0952 |  | cytochrome d ubiquinol oxidase subunit I |
| SAB0953 |  | cytochrome d ubiquinol oxidase subunit II |
| SAB1009 | trxA | thioredoxin |
| SAB1011 | sdhC | succinate dehydrogenase cytochrome b-558 |
| SAB1346 |  | ferredoxin |
| SAB1604 |  | thioredoxin family protein |
| SAB1790 |  | iron-sulfur binding protein |
| SAB2152 |  | formate dehydrogenase homolog |
| SAB2252 |  | thioredoxin reductase |
| SAB2397 |  | NADH-dependent flavin reductase |
| Fermentation | |  |
| SAB0089 |  | alcohol-acetaldehyde dehydrogenase, iron-containing |
| SAB0108 | aldA | aldehyde dehdydrogenase |
| SAB0174 |  | acetyl-CoA/acetoacetyl-CoA transferase |
| SAB0191 |  | zinc-binding dehydrogenase (pseudogene) |
| SAB0195 |  | xylitol dehydrogenase |
| SAB0538 | pta | phosphotransacetylase |
| SAB0557 | adhA | alcohol dehydrogenase I |
| SAB1296 |  | alcohol dehydrogenase |
| SAB1857 |  | aldehyde dehydrogenase |
| SAB2006 |  | aldehyde dehydrogenase |
| SAB2086 |  | alpha-acetolactate decarboxylase |
| SAB2087 |  | alpha-acetolactate synthase |
| **Glycolysis/gluconeogenesis** | | |
| SAB0272 |  | trimethylamine dehydrogenase |
| SAB0648 |  | fructose 1-phosphate kinase |
| SAB0728 | gap | glyceraldehyde-3-phosphate dehydrogenase |
| SAB0729 | pgk | phosphoglycerate kinase |
| SAB0730 | tpiA | triosephosphate isomerase |
| SAB0731 | pgm | 2,3-bisphosphoglycerate-independent phosphoglycerate mutase |
| SAB0732 | eno | enolase 2-phosphoglycerate dehydratase |
| SAB0756 |  | phosphoglycerate mutase |
| SAB0830 |  | glucose-6-phosphate isomerase A |
| SAB0979 |  | pyruvate carboxylase |
| SAB1419 | glkA | glucokinase |
| SAB1546 | gap | glyceraldehyde 3-phosphate dehydrogenase 2 |
| SAB1556 | pyk | pyruvate kinase |
| SAB1557 | pfkA | 6-phosphofructokinase |
| SAB1646 | pckA | phosphoenolpyruvate carboxykinase |
| SAB2009 |  | fructose-bisphosphate aldolase |
| SAB2296 | gpmA | 2,3-bisphosphoglycerate-dependent phosphoglycerate mutase |
| SAB2390 |  | fructose-bisphosphatase |
| SAB2479 | fda | fructose-bisphosphate aldolase class I |
| Pentose phosphate pathway | | |
| SAB1086 |  | ribulose-phosphate 3-epimerase |
| SAB1200 |  | transketolase |
| SAB1366 |  | glucose-6-phosphate 1-dehydrogenase |
| SAB1384 | gnd | 6-phosphogluconate dehydrogenase |
| SAB1639 |  | transaldolase |
| SAB2213 |  | ribose 5-phosphate isomerase A |
| Pyruvate dehydrogenase | | |
| SAB0031 | gloB | hydroxyacylglutathione hydrolase |
| SAB0723 |  | malolactic enzyme |
| SAB0959 | pdhA | pyruvate dehydrogenase E1 component alpha subunit |
| SAB0960 | pdhB | pyruvate dehydrogenase E1 component beta subunit |
| SAB0961 | pdhC | dihydrolipoamide S-acetyltransferase component of pyruvate dehydrogenase complex E2 |
| SAB0962 | pdhD | dihydrolipoamide dehydrogenase component of pyruvate dehydrogenase E3 |
| Sugars |  |  |
| SAB0187 | gutB | zinc-binding sorbitol dehydrogenase |
| SAB0189 |  | zinc-binding sorbitol dehydrogenase |
| SAB0208 |  | ribokinase |
| SAB0248 |  | carbohydrate kinase |
| SAB0503 | araB | L-ribulokinase |
| SAB0520 |  | 3-hexulose-6-phosphate synthase |
| SAB0521 | hxlB | 6-phospho-3-hexuloisomerase |
| SAB1605 |  | endo-1,4-beta-glucanase |
| SAB1924 |  | fructokinase |
| SAB2027 |  | mannose-6-phosphate isomerase |
| SAB2039 | mtlD | mannitol-1-phosphate 5-dehydrogenase |
| SAB2157 |  | butyryl-CoA dehydrogenase |
| SAB2215 |  | epimerase |
| SAB2338 |  | endo-1,4-beta-glucanase |
| SAB2371 |  | phosphoglucomutase |
| SAB2378 | gntK | gluconokinase |
| SAB2517 | pmi | mannose-6-phosphate isomerase |
| **TCA cycle** | | |
| SAB1012 | sdhA | succinate dehydrogenase flavoprotein subunit |
| SAB1013 | sdhB | succinate dehydrogenase iron-sulfur protein |
| SAB1109 | sucC | succinyl-CoA synthetase beta chain |
| SAB1110 | sucD | succinyl-CoA synthetase alpha chain |
| SAB1207 | citB | aconitate hydratase |
| SAB1268 | odhB | dihydrolipoamide succinyltransferase |
| SAB1269 | odhA | 2-oxoglutarate dehydrogenase E1 component |
| SAB1553 | citC | isocitrate dehyrogenase |
| SAB1554 | citZ | citrate synthase II |
| SAB1560 |  | NAD-dependent malic enzyme |
| SAB1784 | citG | fumarate hydratase class-II |
| SAB2068 |  | quinone oxidoreductase |
| SAB2244 | mqo | malate-quinone oxidoreductase 1 |
| SAB2480 | mqo | malate:quinone oxidoreductase |
| Other |  |  |
| SAB0694 |  | glycerate kinase |
| SAB1161 | glpK | glycerol kinase |
| SAB1162 | glpD | glycerol-3-phosphate dehydrogenase |
| SAB1336 | gpsA | glycerol-3-phosphate dehydrogenase |
| SAB1593 | acsA | acetyl-CoA synthetase |
| SAB1594 | acuA | acetoin utilization protein |
| SAB1595 | acuC | acetoin utilization protein |
| SAB2065 |  | membrane-bound oxidoreductase |
| SAB2314 |  | glycerate kinase |
| SAB2343 |  | glutamate synthase-ferredoxin large subunit |
| SAB2413 |  | pyruvate oxidase |
| SAB2474 |  | alpha-acetolactate decarboxylase |
| SAB2482 |  | acetate-CoA ligase |

| Fatty acid and phospholipid metabolism | | |
| --- | --- | --- |
| Biosynthesis | |  |
| SAB0170 |  | 3-ketoacyl-CoA transferase |
| SAB0304 | th1 | acetyl-CoA acetyltransferase protein |
| SAB0526 |  | acetyl-CoA c-acetyltransferase |
| SAB0599 |  | dihydroxyacetone kinase |
| SAB0600 |  | dihydroxyacetone kinase |
| SAB0848 | fabH | 3-oxoacyl-ACP synthase III |
| SAB0849 | fabF | 3-oxoacyl-ACP synthase II |
| SAB0877 | fabI | trans-2-enoyl-ACP reductase |
| SAB0883 |  | UDP-glucose diacylglycerol glucosyltransferase |
| SAB1093 |  | fatty acid/phospholipid synthesis protein |
| SAB1094 | fabD | malonyl CoA-acyl carrier protein transacylase |
| SAB1095 | fabG | 3-oxoacyl-(acyl-carrier protein) reductase |
| SAB1096 | acpP | acyl carrier protein |
| SAB1123 | cdsA | phosphatidate cytidylyltransferase |
| SAB1145 | pgsA | phosphatidylglycerophosphate synthase |
| SAB1180 |  | cardiolipin synthase |
| SAB1399 | accC | acetyl-CoA biotin carboxylase |
| SAB1400 | accB | acetyl-CoA carboxylase biotin carboxyl carrier subunit |
| SAB1441 |  | diacylglycerol kinase |
| SAB1477 | accC | acetyl-CoA biotin carboxylase |
| SAB1478 | accB | biotin carboxyl carrier protein of acetyl-CoA carboxylase |
| SAB1558 | accA | acetyl-CoA carboxylase carboxyl transferase subunit alpha |
| SAB1559 | accD | acetyl-CoA carboxylase carboxyl transferase subunit beta |
| SAB1585 | plsC | 1-acylglycerol-3-phosphate O-acyltransferase |
| SAB1956 | acpS | holo-acyl-carrier protein synthase |
| SAB1972 |  | cardiolipin synthetase |
| SAB1983 | fabZ | 3R-hydroxymyristoyl ACP dehydratase |
| Degradation | |  |
| SAB0038 | plc | 1-phosphatidylinositol phosphodiesterase |
| SAB0171 |  | 3-hydroxyacyl-CoA dehydrogenase |
| SAB0172 | fadD | glutaryl or acyl-CoA dehydrogenase (pseudogene) |
| SAB0173 |  | acyl-CoA synthetase |
| SAB0257 | geh | glycerol ester hydrolase |
| SAB0604 |  | lipase |
| SAB0827 | glpQ | glycerophosphoryl diester phosphodiesterase |
| SAB2504 |  | tributyrin esterase |
| SAB2546 | lip | triacylglycerol lipase precursor |
| Other |  |  |
| SAB0525 |  | long chain fatty acid CoA ligase |
| SAB1092 |  | fatty acid and phospholipid biosynthesis transcriptional regulator |
| Mobile and extrachromosomal element functions | | |
| Prophage functions | | |
| SAB0258 |  | phage-related integrase |
| SAB0259 |  | conserved hypothetical protein |
| SAB0260 |  | hypothetical phage-related protein |
| SAB0261 |  | hypothetical phage-related protein |
| SAB0262 |  | hypothetical phage-related protein |
| SAB0263 |  | hypothetical phage-related protein |
| SAB0264 |  | hypothetical phage-related protein |
| SAB0265 |  | type-I specificity determinant subunit |
| SAB0266 |  | phage-related holin |
| SAB0267 |  | phage-related amidase (pseudogene) |
| SAB0780 |  | phage-associated holin |
| SAB1165 |  | phage-related host factor-I protein |
| SAB1703 |  | phage-related amidase |
| SAB1704 |  | phage-related holin |
| SAB1705 |  | phage-related tail fiber |
| SAB1706 |  | phage-related cell wall hydrolase |
| SAB1722 |  | phage-related head protein |
| SAB1726 |  | phage-associated terminase large subunit |
| SAB1753 |  | phage anti-repressor protein |
| SAB1755 |  | phage anti-repressor protein |
| SAB1775 |  | cmp-binding-factor 1 |
| Miscellaneous regulatory functions | | |
| SAB1728 |  | mobile-element-associated regulatory protein |
| SAB1729 | rinB | int gene transcriptional activator |
| SAB1745 |  | single strand DNA binding protein |

| Transposon functions | | |
| --- | --- | --- |
| SAB0342 |  | integrase |
| SAB0467 |  | transposase (pseudogene) |
| SAB0560 |  | transposase (pseudogene) |
| SAB0572 |  | phage recombinase/integrase |
| SAB0707 |  | transposase |
| SAB0738 |  | transposase |
| SAB0779 |  | phage-related integrase |
| SAB0811 |  | transposase (pseudogene) |
| SAB0850 |  | transposase |
| SAB0902 |  | transposase |
| SAB1059 |  | transposase (pseudogene) |
| SAB1114 |  | integrase/recombinase |
| SAB1166 |  | transposase |
| SAB1167 |  | transposase |
| SAB1298 |  | recombinase |
| SAB1299 |  | transposase for IS-like element |
| SAB1589 |  | transposase |
| SAB1760 |  | integrase |
| SAB1883 |  | transposase |
| SAB1910 |  | integrase |
| SAB1912 |  | integrase |
| SAB2045 |  | transposase |
| SAB2046 |  | transposase |
| SAB2179 |  | transposase |
| SAB2180 |  | transposase |
| SAB2210 |  | transposase |
| SAB2360 |  | truncated transposase |
| Protein fate | | |
| Degradation of proteins, peptides, and glycopeptides | | |
| SAB0042 |  | aminoacylase |
| SAB0475 | clpC | endopeptidase |
| SAB0695 | pepT | aminotripeptidase |
| SAB0722 | clpP | ATP-dependent Clp protease proteolytic subunit |
| SAB0808 |  | cytosol aminopeptidase |
| SAB0843 | clpB | putative ATP-dependent protease protein |
| SAB0867 | pepB | thimet oligopeptidase |
| SAB0888 |  | serine proteinase |
| SAB0913 | sspC | cysteine protease |
| SAB0914 | sspB | cysteine protease precursor |
| SAB0915 | sspA | glutamyl endopeptidase serine protease |
| SAB1124 |  | zinc metalloprotease |
| SAB1141 |  | protease (zinc) protein |
| SAB1221 |  | peptidase |
| SAB1240 |  | oligoendopeptidase F |
| SAB1253 |  | peptidase |
| SAB1275 |  | carboxy-terminal processing proteinase |
| SAB1372 |  | protease |
| SAB1385 |  | peptidase T |
| SAB1483 |  | proteinase |
| SAB1484 |  | proteinase |
| SAB1534 | clpX | ATP-dependent Clp protease ATP-binding subunit |
| SAB1567 |  | Xaa-Pro dipeptidase homolog |
| SAB1586 |  | serine protease |
| SAB1611 |  | Xaa-His dipeptidase homolog |
| SAB1679 |  | serine protease precursor |
| SAB1811 |  | aminopeptidase |
| SAB1934 |  | O-sialoglycoprotein endopeptidase |
| SAB1936 |  | glycoprotein endopeptidase |
| SAB2051 |  | hemolysin |
| SAB2206 |  | peptidase |
| SAB2423 | clpL | ATP-dependent proteinase chain |
| SAB2512 | aur | zinc metalloproteinase aureolysin |
| SAB2566 | pcp | pyrrolidone-carboxylate peptidase |
| Protein and peptide secretion and trafficking | | |
| SAB0296 |  | sec-independent protein translocase protein |
| SAB0297 |  | protein translocase subunit |
| SAB0434 |  | signal peptidase II |
| SAB0440 |  | sec-independent hydrolase |
| SAB0486 | secE | preprotein translocase subunit |
| SAB0705 | secA | preprotein translocase subunit |
| SAB0734 |  | protein-export translocase membrane protein |
| SAB0833 | spsA | type-I signal peptidase |
| SAB0834 | spsB | type-I signal peptidase |
| SAB1060 | lspA | lipoprotein signal peptidase |
| SAB1099 |  | signal recognition particle protein |
| SAB1101 | ffh | signal recognition particle protein |
| SAB1506 | secF | protein-export membrane protein |
| SAB1522 |  | leader peptidase protein |
| SAB1774 |  | peptidyl-prolyl cis-isomerase |
| SAB2102 | secA | preprotein translocase subunit |
| SAB2524 |  | preprotein translocase SecA subunit-like protein (pseudogene) |
| Protein folding and stabilization | | |
| SAB0461 |  | heat shock chaperonin protein 33 |
| SAB0502 |  | chaperone protein Hsp31 |
| SAB0821 |  | peptidyl-prolyl cis-trans isomerase |
| SAB1115 | hslV | ATP-dependent protease heat shock protein |
| SAB1116 | hslU | ATP-dependent protease ATP-binding subunit of heat shock protein |
| SAB1451 | dnaJ | chaperone protein |
| SAB1452 | dnaK | chaperone protein |
| SAB1453 | grpE | heat shock molecular chaperone protein |
| SAB1535 | tig | trigger factor prolyl isomerase |
| SAB1913 | groEL | 60 kDa chaperonin protein |
| SAB1914 | groES | 10 kDa heat shock chaperonin protein |
| Protein modification and repair | | |
| SAB0120 |  | 4'-phosphopantetheinyl transferase superfamily protein |
| SAB0277 |  | lipoate-protein ligase |
| SAB0539 |  | lipoate-protein ligase A protein |
| SAB0714 | lgt | prolipoprotein diacylglyceryl transferase |
| SAB0893 |  | Lipoate-protein ligase A |
| SAB0957 |  | peptide deformylase |
| SAB1079 | def | peptide deformylase 1 |
| SAB1217 | msrA1 | peptide methionine sulfoxide reductase 1 |
| SAB1278 |  | peptide methionine sulfoxide reductase |
| SAB1279 | msrA | peptide methionine sulfoxide reductase |
| SAB1820 |  | methionyl aminopeptidase |
| SAB2535 |  | peptide methionine sulfoxide reductase |
| Other |  |  |
| SAB0451 |  | peptidyl-tRNA hydrolase |
| SAB0737 | smpB | tmRNA and SsrA-binding protein |
| **Ribosomal proteins: synthesis and modification** | | |
| SAB0015 |  | 50S ribosomal protein L9 |
| SAB0292 |  | serine N-acetyltransferase |
| SAB0316 | rpsF | 30S ribosomal protein S6 |
| SAB0318 | rpsR | 30S ribosomal protein S18 |
| SAB0450 | prs | 50S ribosomal protein L25 general stress protein |
| SAB0485 | rpmG | 50S ribosomal protein L33 |
| SAB0488 | rplK | 50S ribosomal protein L11 |
| SAB0489 | rplA | 50S ribosomal protein L1 |
| SAB0490 | rplJ | 50S ribosomal protein L10 |
| SAB0491 | rplL | 50S ribosomal protein L7/L12 |
| SAB0495 |  | ribosomal protein |
| SAB0496 | rpsL | 30S ribosomal protein S12 |
| SAB0497 | rpsG | 30S ribosomal protein S7 |
| SAB0992 | rpmF | 50S ribosomal protein L32 |
| SAB1088 | rpmB | 50S ribosomal protein L28 |
| SAB1102 | rpsP | 30S ribosomal protein S16 |
| SAB1105 | rplS | 50S ribosomal protein L19 |
| SAB1118 | rpsB | 30S ribosomal protein S2 |
| SAB1130 |  | ribosomal protein |
| SAB1135 | rpsO | 30S ribosomal protein S15 |
| SAB1193 | rpmG | 50S ribosomal protein L33 |
| SAB1194 | rpsN | 30S ribosomal protein S14 |
| SAB1338 | rpsA | 30S ribosomal protein S1 |
| SAB1402 |  | proline dipeptidase |
| SAB1423 | rpmG | 50S ribosomal protein L33 |
| SAB1447 | rpsU | 30S ribosomal protein S21 |
| SAB1450 |  | methyltransferase |
| SAB1458 | rpsT | 30S ribosomal protein S20 |
| SAB1514 | rpmA | 50S ribosomal protein L27 |
| SAB1516 | rplU | 50S ribosomal protein L21 |
| SAB1538 | rplT | 50S ribosomal protein L20 |
| SAB1539 | rpmI | 50S ribosomal protein L35 |
| SAB1578 | rpsD | 30S ribosomal protein S4 |
| SAB1935 |  | ribosomal-protein-alanine acetyltransferase |
| SAB2004 | rpmE | 50S ribosomal protein L31 type B |
| SAB2090 | rpsI | 30S ribosomal protein S9 |
| SAB2091 | rplM | 50S ribosomal protein L13 |
| SAB2096 | rplQ | 50S ribosomal protein L17 |
| SAB2098 | rpsK | 30S ribosomal protein S11 |
| SAB2099 | rpsM | 30S ribosomal protein S13 |
| SAB2103 | rplO | 50S ribosomal protein L15 |
| SAB2104 | rpmD | 50S ribosomal protein L30 |
| SAB2105 | rpsE | 30S ribosomal protein S5 |
| SAB2106 | rplR | 50S ribosomal protein L18 |
| SAB2107 | rplF | 50S ribosomal protein L6 |
| SAB2108 | rpsH | 30S ribosomal protein S8 |
| SAB2109 | rpsN | 30S ribosomal protein S14 |
| SAB2110 | rplE | 50S ribosomal protein L5 |
| SAB2111 | rplX | 50S ribosomal protein L24 |
| SAB2112 | rplN | 50S ribosomal protein L14 |
| SAB2113 | rpsQ | 30S ribosomal protein S17 |
| SAB2114 | rpmC | 50S ribosomal protein L29 |
| SAB2115 | rplP | 50S ribosomal protein L16 |
| SAB2116 | rpsC | 30S ribosomal protein S3 |
| SAB2117 | rplV | 50S ribosomal protein L22 |
| SAB2118 | rpsS | 30S ribosomal protein S19 |
| SAB2119 | rplB | 50S ribosomal protein L2 |
| SAB2120 | rplW | 50S ribosomal protein L23 |
| SAB2121 | rplD | 50S ribosomal protein L4 |
| SAB2122 | rplC | 50S ribosomal protein L3 |
| SAB2123 | rpsJ | 30S ribosomal protein S10 |
| SAB2590 | rpmH | 50S ribosomal protein L34 |
| Translation factors | | |
| SAB0498 | fus | translation elongation factor G (EF-G) |
| SAB0499 | tuf | translation elongation factor Tu (EF-Tu) |
| SAB0706 | prfB | peptide chain release factor 2 |
| SAB0886 | prfC | peptide chain release factor 3 |
| SAB0974 |  | GTP-binding elongation factor protein |
| SAB1119 | tfs | elongation factor Ts |
| SAB1121 | frr | ribosome recycling factor |
| SAB1131 | infB | translation initiation factor IF-2 |
| SAB1540 | infC | translation initiation factor IF-3 |
| SAB2002 | prfA | peptide chain release factor 1 |
| SAB2100 | infA | translation initiation factor IF-1 |
| **tRNA aminoacylation** | | |
| SAB0009 | serS | seryl-tRNA synthetase |
| SAB0439 | metG | ethionyl-tRNA synthetase |
| SAB0466 | lysS | lysyl-tRNA synthetase |
| SAB0478 | gltX | glutamyl-tRNA synthetase |
| SAB0480 | cysS | cysteinyl-tRNA synthetase |
| SAB0559 |  | arginyl-tRNA synthetase |
| SAB0863 | trpS | tryptophanyl-tRNA synthetase |
| SAB1002 | pheS | phenylalanyl-tRNA synthetase alpha chain |
| SAB1003 | pheT | phenylalanyl-tRNA synthetase beta chain |
| SAB1057 | ileS | isoleucyl-tRNA synthetase |
| SAB1080 | fmt | methionyl-tRNA formyltransferase |
| SAB1125 | proS | prolyl-tRNA synthetase |
| SAB1318 | asnS | asparaginyl-tRNA synthetase |
| SAB1437 | glyS | glycyl-tRNA synthetase |
| SAB1489 | alaS | alanyl-tRNA synthetase |
| SAB1499 | aspS | aspartyl-tRNA synthetase |
| SAB1500 | hisS | histidyl-tRNA synthetase |
| SAB1524 | valS | valyl-tRNA synthetase |
| SAB1542 | thrS | threonyl-tRNA synthetase 1 |
| SAB1587 | tyrS | tyrosyl-tRNA synthetase |
| SAB1602 |  | phenylalanyl-tRNA synthetase beta subunit |
| SAB1618 | leuS | leucyl-tRNA synthetase |
| SAB1832 | gatB | glutamyl-tRNA amidotransferase subunit B |
| SAB1833 | gatA | glutamyl-tRNA amidotransferase subunit A |
| SAB1834 | gatC | glutamyl-tRNA amidotransferase subunit C |
| **tRNA and rRNA base modification** | | |
| SAB0482 |  | tRNA/rRNA methyltransferase |
| SAB0874 |  | RNA pseudoridylate synthase |
| SAB1001 |  | rRNA methyltransferase |
| SAB1081 | sun | RNA-binding protein |
| SAB1104 | trmD | tRNA guanine-N(1)-methyltransferase |
| SAB1133 | truB | tRNA-pseudouridine 5S synthase |
| SAB1164 | miaA | tRNA delta(2)-isopentenylpyrophosphate transferase |
| SAB1354 | rluB | ribosomal large subunit pseudouridine synthase B |
| SAB1492 | trmU | 5-methylaminomethyl-2-thiouridylate-methyltransferase tRNA |
| SAB1508 | tgt | queuine tRNA-ribosyltransferase |
| SAB1509 | queA | S-adenosylmethionine:tRNA ribosyltransferase-isomerase |
| SAB1608 |  | tRNA guanine methyltransferase |
| SAB1613 |  | 16S RNA pseudouridylate synthase |
| SAB1783 |  | RNA pseudouridylate synthase |
| SAB1789 |  | rRNA methylase homolog |
| SAB1829 |  | RNA methyltransferase |
| SAB2092 |  | tRNA pseudouridine synthase A |
| **Purines, pyrimidines, nucleosides, and nucleotides** | | |
| **2'-Deoxyribonucleotide metabolism** | | |
| SAB0681 |  | ribonucleotide reductase |
| SAB0682 | rir1 | ribonucelotide diphosphate reductase alpha chain |
| SAB0683 | rir2 | ribonucelotide diphosphate reductase beta chain |
| SAB1282 | thyA | thymidylate synthase |
| SAB2490 | nrdG | anaerobic ribonucleotide reductase small subunit |
| SAB2491 | nrdD | anaerobic ribonucleotide reductase large subunit |
| Nucleotide and nucleoside interconversions | | |
| SAB0431 | tmk | thymidylate kinase |
| SAB0507 |  | deoxypurine kinase subunit |
| SAB0508 |  | deoxypurine kinase subunit |
| SAB1073 | gmk | guanylate kinase |
| SAB1120 | smbA | uridylate kinase |
| SAB1195 |  | GMP reductase |
| SAB1331 |  | nucleoside-diphosphate kinase |
| SAB1340 | cmk | cytidylate kinase |
| SAB2003 | tdk | thymidine kinase |
| SAB2101 | adk | adenylate kinase |
| Other |  |  |
| SAB0023 |  | 5' nucleotidase |
| SAB0077 | deoC1 | deoxyribose-phosphate aldolase 1 |
| SAB0078 | deoB | phosphopentomutase |
| SAB0086 | cpdB | 2',3'-cyclic-nucleotide 2'-phosphodiesterase |
| SAB0457 |  | polyribonucleotide nucleotidyltransferase |
| SAB0890 |  | 5' nucleotidase |
| SAB2021 | deoC2 | deoxyribose-phosphate aldolase |
| Purine ribonucleotide biosynthesis | | |
| SAB0017 | purA | adenylosuccinate synthetase |
| SAB0340 | guaB | inositol-monophosphate dehydrogenase |
| SAB0341 | guaA | glutamine-hydrolyzing GMP synthase |
| SAB0449 | prs | ribose-phosphate pyrophosphokinase |
| SAB0931 | purE | phosphoribosylaminoimidazole carboxylase catalytic subunit |
| SAB0932 | purK | phosphoribosylaminoimidazole carboxylase |
| SAB0933 | purC | phosphoribosylaminoimidazole-succinocarboxamide synthase |
| SAB0934 | purS | phosphoribosylformylglycinamidine synthase |
| SAB0935 | purQ | phosphoribosylformylglycinamidine synthase I |
| SAB0936 | purL | phosphoribosylformylglycinamidine synthase I |
| SAB0937 | purF | phosphoribosylpyrophosphate amidotransferase |
| SAB0938 | purM | phosphoribosylformylglycinamidine cyclo-ligase |
| SAB0939 | purN | phosphoribosylformylglycinamidine formyltransferase |
| SAB0940 | purH | bifunctional purine biosynthesis protein with phosphoribosylaminoimidazolecarboxamide formyltransferase and IMP cyclohydrolase |
| SAB0941 | purD | phosphoribosylamine-glycine ligase |
| SAB1360 |  | ADP-ribose pyrophosphatase |
| SAB1843 | purB | adenylosuccinate lyase |
| Pyrimidine ribonucleotide biosynthesis | | |
| SAB1064 | pyrB | aspartate carbamoyltransferase catalytic chain A |
| SAB1065 | pyrC | dihydroorotase |
| SAB1066 | pyrAA | carbamoyl-phosphate synthase small chain |
| SAB1067 | pyrAB | carbamoyl-phosphate synthase small chain |
| SAB1068 | pyrF | orotidine-5-phosphate decarboxylase |
| SAB1069 | pyrE | orotate phosphoribosyltransferase |
| SAB2011 | pyrG | CTP synthase |
| SAB2464 | pyrD | dihydroorotate dehydrogenase |
| Salvage of nucleosides and nucleotides | | |
| SAB0075 | deoD1 | purine nucleoside phosphorylase |
| SAB0182 |  | inosine-uridine preferring nucleoside hydrolase family |
| SAB0338 | xpt | xanthine phosphoribosyltransferase |
| SAB0459 | hpt | hypoxanthine-guanine phosphoribosyltransferase |
| SAB1440 | cdd | cytidine deaminase |
| SAB1482 | udk | uridine kinase |
| SAB1504 | apt | adenine phosphoribosyltransferase |
| SAB1996 | upp | uracil phosphoribosyltransferase |
| SAB2020 | pyn | pyrimidine nucleoside phosphorylase |
| SAB2022 | deoD2 | purine nucleoside phosphorylase |
| SAB2155 |  | inosine-uridine preferring nucleoside hydrolase |
| **Regulatory functions** | | |
| **DNA interactions** | | |
| SAB0018 | yycF | two-component response regulator |
| SAB0033 |  | nitrogen regulation protein |
| SAB0041 |  | transcriptional regulator |
| SAB0047 |  | transcriptional regulator |
| SAB0051 | sarS | transcriptional regulator |
| SAB0074 |  | transcriptional regulator, GntR family |
| SAB0133 |  | transcriptional regulator RpiR family |
| SAB0203 |  | transcriptional regulator GntR family |
| SAB0211 |  | ribose transcriptional repressor LacI family |
| SAB0283 |  | regulatory protein MarR family |
| SAB0425 |  | trehalose operon transcriptional repressor |
| SAB0445 | purR | pur operon transcriptional repressor |
| SAB0468 |  | transcriptional regulator of GntR family |
| SAB0472 |  | transcriptional repressor of stress genes |
| SAB0608 |  | two-component response regulator |
| SAB0609 |  | two-component sensor histidine kinase |
| SAB0616 |  | transcriptional regulator |
| SAB0621 |  | transcriptional regulator LysR family |
| SAB0635 |  | transcriptional regulator MarR family |
| SAB0646 |  | transcriptional regulator protein |
| SAB0647 |  | transcriptional repressor of fructose operon |
| SAB0727 | gapR | glycolytic operon regulator |
| SAB0844 |  | regulatory protein LysR family |
| SAB0964 |  | transcriptional regulator |
| SAB1117 | codY | GTP-sensing transcriptional pleiotropic repressor |
| SAB1139 |  | GntR family transcriptional regulator |
| SAB1144 |  | DNA-binding protein |
| SAB1197 | dinR | DNA damage SOS regulatory repressor protein |
| SAB1359 | fur | iron transcriptional regulator |
| SAB1367 |  | transcriptional regulator AraC family |
| SAB1369 | malR | maltose operon transcription repressor |
| SAB1393 | argR | arginine repressor |
| SAB1426 | zur | zinc-specific metalloregulator |
| SAB1454 | hrcA | heat-inducible transcription repressor |
| SAB1596 | ccpA | catabolite control protein A |
| SAB1780 |  | DNA-binding protein |
| SAB1794 | perR | peroxide operon regulator |
| SAB1870 |  | GntR family transcriptional regulator |
| SAB1926 | scrR | sucrose operon repressor |
| SAB2029 | czrA | zinc and cobalt transport repressor protein |
| SAB2037 |  | transcription antiterminator |
| SAB2077 | lacR | lactose phosphotransferase system repressor |
| SAB2078 |  | regulatory protein |
| SAB2081 |  | MerR family transcriptional regulator |
| SAB2170 |  | transcriptional regulator |
| SAB2192 |  | transcriptional regulator |
| SAB2201 |  | transcriptional regulator |
| SAB2233 |  | regulatory protein TetR family |
| SAB2265 |  | transcriptional regulator MarR family |
| SAB2269 |  | regulatory protein |
| SAB2369 |  | DNA modification methylase |
| SAB2379 | gntR | gluconate operon transcription repressor |
| SAB2380 |  | transcriptional regulator MerR family |
| SAB2394 |  | transcriptional regulator MarR family |
| SAB2416 |  | transcriptional regulator LysR family |
| SAB2427 |  | conserved hypothetical protein TetR family |
| SAB2445 |  | regulatory protein TetR family |
| SAB2503 |  | MarR family transcriptional regulator |
| SAB2506 |  | transcriptional regulator |
| SAB2511 | argR | arginine repressor |
| SAB2540 | icaR | ica operon transcription regulator |
| SAB2573 |  | DNA-binding protein |
| Other |  |  |
| SAB0088 |  | transcriptional regulator |
| SAB0131 |  | glucokinase regulator-related protein |
| SAB0247 |  | perfringolysin O regulatory protein |
| SAB0254 |  | transcription regulator |
| SAB0299 |  | DNA-binding protein |
| SAB0545 |  | DNA-binding protein |
| SAB0569 | sarA | staphylococcal accessory regulator A |
| SAB0699 |  | lipophilic regulator protein |
| SAB0781 |  | lytic enzyme |
| SAB0891 |  | transcription factor |
| SAB1218 |  | peptide methionine sulfoxide reductase regulator |
| SAB1256 |  | transcriptional regulator |
| SAB1622 | rot | repressor of toxins transcriptional regulator |
| SAB1757 |  | transcriptional repressor |
| SAB1836 |  | transcriptional regulator |
| SAB1920 | agrB | accessory gene regulator B |
| SAB1921 | agrD | autoinducer peptide precursor |
| SAB1922 | agrC | accessory gene regulator C |
| SAB1923 | agrA | accessory gene regulator A |
| SAB1978 | tenA | transcriptional activator |
| SAB2031 |  | membrane-embedded lytic regulatory protein |
| SAB2083 |  | transcriptional regulator |
| SAB2167 | sarR | staphylococcal accessory regulator protein |
| SAB2187 |  | transcriptional regulator |
| SAB2188 |  | extragenic supressor protein |
| SAB2249 |  | transcriptional regulator |
| SAB2406 |  | surface-exposed regulatory protein |
| SAB2444 |  | membrane-embedded regulatory protein |
| Protein interactions | | |
| SAB0019 | yycG | two-component sensor histidine kinase |
| SAB1084 | pknB | protein kinase |
| SAB1813 |  | protein tyrosine phosphatase |
| SAB1951 | rsbV | anti-sigma B factor antagonist |
| SAB1952 | rsbU | indirect positive regulator of sigma B |
| RNA interactions | | |
| SAB0183 |  | transcriptional regulator, BglB family (pseudogene) |
| SAB1062 | pyrR | bifunctional protein including pyrimidine operon regulatory protein and uracil phosphoribosyltransferase |
| SAB1159 | glpP | glycerol uptake operon antiterminator regulatory protein |
| SAB1214 |  | transcription antiterminator |
| SAB2515 |  | antiterminator transcriptional regulator |

| Signal transduction | | |
| --- | --- | --- |
| Two-component systems | | |
| SAB0160 | uhpT | hexose phosphate transport protein |
| SAB0161 |  | two-component response regulator |
| SAB0162 |  | two-component sensor histidine kinase |
| SAB0199 | lytS | two-component sensor histidine kinase |
| SAB0200 | lytR | two-component sensor histidine kinase |
| SAB1270 | arlS | two-component sensor histidine kinase |
| SAB1271 | arlR | two-component response regulator |
| SAB1352 | srrB | staphylococcal respiratory response protein B |
| SAB1353 | truncated srrA | staphylococcal respiratory response protein A (pseudogene) |
| SAB1551 | phoR | alkaline phosphatase synthesis sensor protein |
| SAB1552 | phoP | alkaline phosphatase synthesis transcriptional regulatory protein |
| SAB1781 |  | two-component response regulator protein |
| SAB1782 |  | two-component response system sensor histidine kinase |
| SAB1816 |  | two-component response regulator |
| SAB1817 | vraS | two-component sensor histidine kinase |
| SAB1963 | kdpD | two component sensor protein |
| SAB1964 | kdpE | two component sensor protein |
| SAB2240 |  | two-component response regulator |
| SAB2241 |  | two-component sensor histidine kinase sensor |
| SAB2270 |  | two-component response regulator |
| SAB2271 |  | two component sensor histidine kinase |
| SAB2499 |  | two-component sensor histidine kinase |
| SAB2500 |  | two-component sensor response regulator |
| Transcription | |  |
| Degradation of RNA | | |
| SAB0446 | yabJ | translation initiation inhibitor |
| SAB0736 | rnr | ribonuclease R |
| SAB1108 |  | ribonuclease HII |
| SAB1136 | pnpA | polyribonucleotide nucleotidyltransferase |
|  | | |
| SAB0493 | rpoB | DNA-dependent RNA polymerase beta subunit |
| SAB0494 | rpoC | DNA-directed RNA polymerase beta' subunit |
| SAB1074 |  | DNA-directed RNA polymerase omega chain |
| SAB2012 | rpoE | DNA-directed RNA polymerase delta subunit |
| SAB2097 | rpoA | DNA-directed RNA polymerase alpha chain |
| Other |  |  |
| SAB1430 |  | ATP-dependent RNA helicase |
| SAB1965 |  | ATP-dependent RNA helicase |
| RNA processing | |  |
| SAB1097 |  | ribonuclease III |
| SAB1103 | rimM | 16S rRNA processing protein |
| SAB1132 | rbfA | ribosome-binding factor A |
| SAB1321 | papS | PolyA polymerase family protein |
| SAB2589 | rnpA | ribonuclease P protein component |
| Transcription factors | | |
| SAB0484 |  | RNA polymerase sigma factor |
| SAB0487 | nusG | transcription antitermination protein |
| SAB1128 | nusA | transcription termination-antitermination factor |
| SAB1397 | nusB | N utilization substance protein B |
| SAB1433 | rpoD | RNA polymerase sigma factor |
| SAB1481 | greA | transcription elongation factor |
| SAB1805 |  | regulatory protein |
| SAB1950 | rsbW | anti-sigma B factor |
| SAB2005 | rho | transcription termination factor |
| SAB2189 |  | regulatory protein DeoR family |
| Transport and binding proteins | | |
| Amino acids, peptides and amines | | |
| SAB0010 |  | amino acid transporter |
| SAB0126 | brnQ | branched-chain amino acid transport system carrier protein |
| SAB0144 |  | oligopeptide transport ATP-binding protein (pseudogene) |
| SAB0145 |  | oligopeptide ABC transporter permease protein |
| SAB0146 |  | oligopeptide ABC transporter |

| SAB0243 | brnQ | branched-chain amino acid transport system II carrier protein |
| --- | --- | --- |
| SAB0523 | proP | proline/betaine transporter |
| SAB0534 |  | membrane-embedded amino acid transporter |
| SAB0671 |  | glycine betaine/carnitine/choline ATP-binding ABC transport protein |
| SAB0672 |  | glycine betaine/carnitine/choline ATP-binding ABC transport protein |
| SAB0676 |  | di-tripeptide ABC transporter protein |
| SAB0852 | oppB | oligopeptide transport system permease protein |
| SAB0853 | oppC | oligopeptide transport system permease protein |
| SAB0854 | oppD | oligopeptide transport system ATP-binding protein |
| SAB0855 | oppF | oligopeptide transport system ATP-binding protein |
| SAB0856 |  | oligopeptide binding protein |
| SAB0857 |  | oligopeptide transport system permease protein |
| SAB0858 |  | oligopeptide transport system permease protein |
| SAB0859 |  | oligopeptide transport system ATP-binding protein |
| SAB0860 | appD | oligopeptide transport system ATP-binding protein |
| SAB0861 |  | oligopeptide ATP-binding protein |
| SAB0862 |  | oligopeptide binding protein (pseudogene) |
| SAB0879 |  | sodium/alanine symporter family protein |
| SAB0965 |  | spermidine/putrescine ATP-binding ABC transporter protein |
| SAB0966 |  | spermidine/putrescine ABC transporter permease protein (pseudogene) |
| SAB0967 |  | spermidine/putrescine ABC transporter permease protein |
| SAB0968 |  | spermidine/putrescine-binding periplasmic protein precursor |
| SAB1191 |  | amino acid permease |
| SAB1206 | opuD | glycine betaine transporter |
| SAB1213 |  | amino acid carrier protein |
| SAB1234 |  | oligopeptide transporter ATPase |
| SAB1235 |  | oligopeptide transporter ATPase |
| SAB1236 |  | oligopeptide membrane permease |
| SAB1237 |  | oligopeptide membrane permease |
| SAB1263 | brnQ | branched-chain amino acid carrier II protein |
| SAB1302 |  | amino acid permease protein |
| SAB1541 | lysP | lysine specific permease |
| SAB1555 | cycA | D-serine, D-alanine, glycine transporter |
| SAB1791 | glnQ | glutamate ATP-binding ABC transporter |
| SAB1792 |  | substrate-binding glutamine ABC transporter |
| SAB1835 | putP | high affinity proline permease |
| SAB2066 | opuD | glycine betaine transporter |
| SAB2159 |  | membrane-bound urea transporter |
| SAB2193 |  | amino acid permease |
| SAB2224 |  | sodium/glutamate symporter |
| SAB2263 | gltT | proton, sodium-glutamate symport protein |
| SAB2292 |  | ABC transporter |
| SAB2293 |  | ABC transporter permease |
| SAB2294 |  | extracellular amino acid binding ABC transporter |
| SAB2320 |  | amino acid ABC transporter |
| SAB2327 | opuCD | betaine-carnitine-choline ABC transporter |
| SAB2328 | opuCC | betaine-carnitine-choline ABC transporter |
| SAB2329 | opuCB | betaine-carnitine-choline ABC transporter |
| SAB2330 | opuCA | betaine-carnitine-choline ABC transporter |
| SAB2332 |  | amino acid transporter permease |
| SAB2346 | opp1F | oligopeptide transporter putative ATPase domain |
| SAB2347 | opp1D | oligopeptide transporter putative ATPase domain |
| SAB2348 | opp1C | oligopeptide transporter putative membrane permease domain |
| SAB2349 | opp1B | oligopeptide transporter putative membrane permease domain |
| SAB2350 | opp1A | oligopeptide transporter putative substrate binding domain |
| SAB2476 |  | cationic amino acid transporter |
| SAB2489 | cudT | choline transporter |
| SAB2508 | arcD | arginine-ornithine antiporter |
| Anions |  |  |
| SAB0242 |  | formate/nitrite transport protein |
| SAB0613 |  | low-affinity inorganic phosphate transporter |
| SAB0639 |  | membrane transport protein |
| SAB1241 |  | phosphate transport system protein |
| SAB1242 |  | phosphate import ATP-binding protein |
| SAB1243 |  | phosphate ABC transporter permease |
| SAB1244 |  | phosphate ABC transporter permease |
| SAB1245 |  | phosphate-binding lipoprotein |
| SAB2149 | modC | molybdenum transport ATP-binding protein |
| SAB2150 | modB | molybdate transport permease protein |
| SAB2151 | modA | molybdate-binding protein |
| SAB2267 | narT | nitrite extrusion protein |
| SAB2281 |  | fumarate and nitrate reduction regulatory protein |
| SAB2283 |  | nitrite transporter |
| SAB2571 |  | 2-oxoglutarate-malate translocator |
| Carbohydrates, organic alcohols, and acids | | |
| SAB0049 | lldP | L-lactate permease |
| SAB0129 | glcA | phosphotransferase system enzyme II glucose-specific factor IIA |
| SAB0132 |  | PTS system sucrose-specific IIBC component |
| SAB0151 |  | multiple sugar-binding transport ATP-binding protein |
| SAB0152 |  | maltose/maltodextrin transport system protein |
| SAB0153 |  | maltose/maltodextrin transport permease protein |
| SAB0154 |  | maltose/maltodextrin transport system protein |
| SAB0181 |  | phosphotransferase system enzyme II |
| SAB0184 |  | phosphotransferase system enzyme |
| SAB0185 |  | phosphotransferase system galactitol-specific enzyme II, B component |
| SAB0186 |  | phosphotransferase system galactitol-specific enzyme II, C component |
| SAB0204 |  | PTS system transport protein |
| SAB0209 |  | ribose permease transport protein |
| SAB0279 |  | PTS system component |
| SAB0280 |  | PTS system component |
| SAB0281 |  | PTS system regulator |
| SAB0333 |  | proton/sodium-glutamate symport protein |
| SAB0423 |  | PTS system trehalose-specific IIBC component |
| SAB0649 | fruA | fructose specific permease |
| SAB0713 | hprK | HPr(Ser) kinase phosphatase |
| SAB0949 | ptsH | histidine-containing phosphocarrier protein |
| SAB0950 | ptsI | phosphoenolpyruvate-protein phosphatase |
| SAB1277 |  | phosphotransferase system glucose-specific IIA component |
| SAB1584 |  | PTS system N-acetylglucosamine-specific IIABC component |
| SAB2036 | mtlA | PTS system mannitol-specific IIBC component |
| SAB2038 | mtlF | PTS system mannitol-specific IIA component |
| SAB2071 | lacE | PTS system lactose-specific IIBC component |
| SAB2072 | lacF | PTS system, lactose-specific IIA component |
| SAB2128 | glcU | glucose uptake protein |
| SAB2200 |  | PTS system arbutin-like IIBC component |
| SAB2245 | lldP | L-lactate permease |
| SAB2257 | scrA | PTS system sucrose-specific IIBC component |
| SAB2377 | gntP | gluconate permease |
| SAB2412 | glcB | PTS system glucose-specific IIABC component |
| SAB2492 |  | magnesium citrate secondary transporter |
| SAB2516 |  | PTS system fructose-specific II component |
| I**ron and cation-carrying compounds** | | |
| SAB0052 | sirC | siderophore transport protein |
| SAB0053 | sirB | siderophore transport protein |
| SAB0057 |  | siderophore biosynthesis protein, lucC family |
| SAB0059 |  | siderophore biosynthesis protein, lucA family |
| SAB0060 |  | siderophore biosynthesis protein, lucC family |
| SAB0109 |  | cation-efflux system membrane protein |
| SAB0119 |  | surfactin/siderophore synthetase |
| SAB0163 |  | periplasmic iron binding protein |
| SAB0251 |  | transport protein |
| SAB0562 |  | iron-binding transport protein |
| SAB0563 |  | iron(III) ABC transporter permease protein |
| SAB0574 |  | Na+/H+ antiporter protein |
| SAB0575 |  | Na+/H+ antiporter protein |
| SAB0576 |  | Na+/H+ antiporter protein |
| SAB0577 |  | Na+/H+ antiporter protein |
| SAB0578 |  | Na+/H+ antiporter protein |
| SAB0579 |  | Na+/H+ antiporter protein |
| SAB0580 |  | Na+/H+ antiporter protein |
| SAB0581 | mntC | iron-repressed transport lipoprotein |
| SAB0582 | mntB | cation ABC transporter |
| SAB0583 | mntA | cation ATP-binding ABC transporter |
| SAB0584 | mntR | iron dependent repressor |
| SAB0596 | fhuA | ferrichrome transport ATP-binding protein |
| SAB0597 | fhuB | ferrichrome transport permease |
| SAB0598 | fhuD | ferrichrome transport permease |
| SAB0651 |  | magnesium and cobalt efflux protein |
| SAB0685 | sstA | FecCD family ferrichrome transporter permease |
| SAB0686 | sstB | FecCD family ferrichrome ATP-binding ABC transporter |
| SAB0687 | sstC | ferrichrome ATP-binding ABC transporter (pseudogene) |
| SAB0688 | sstD | ferrichrome ABC transporter lipoprotein |
| SAB0809 |  | transporter protein |
| SAB0813 | mnhG | Na+/H+ antiporter subunit |
| SAB0814 | mnhF | Na+/H+ antiporter subunit |
| SAB0815 | mnhE | Na+/H+ antiporter subunit |
| SAB0816 | mnhD | Na+/H+ antiporter subunit |
| SAB0817 | mnhC | Na+/H+ antiporter subunit |
| SAB0818 | mnhC | Na+/H+ antiporter subunit |
| SAB0819 | mnhA | Na+/H+ antiporter subunit |
| SAB0875 |  | magnesium transporter |
| SAB0876 |  | Na+/H+ antiporter protein |
| SAB0889 |  | Na+ transporting ATP synthase |
| SAB0904 |  | iron transport protein |
| SAB0905 |  | iron transport protein |
| SAB0942 |  | cobalt transport protein |
| SAB0954 |  | potassium uptake protein |
| SAB0971 |  | Mn2+/Fe2+ transporter |
| SAB0997 | isdE | iron transport lipoprotein |
| SAB0998 | isdF | iron transport permease protein |
| SAB1825 | ftn | ferritin |
| SAB1853 |  | sodium-sulfate symport protein |
| SAB1882 |  | sodium transport protein |
| SAB1927 |  | ammonium transporter |
| SAB1960 | kdpC | potassium-transporting ATPase C chain |
| SAB1961 | kdpB | potassium-transporting ATPase B chain |
| SAB1962 | kdpA | potassium-transporting ATPase A chain |
| SAB2030 | czrB | cation-efflux system membrane protein |
| SAB2056 |  | ferrichrome ABC transporter |
| SAB2057 |  | ferrichrome ABC transporter |
| SAB2058 |  | ferrichrome ABC transporter lipoprotein |
| SAB2093 |  | cobalt transport protein |
| SAB2156 | fhuD2 | ferrichrome-binding protein |
| SAB2173 |  | truncated Na+/H+ antiporter |
| SAB2203 |  | Na+/H+ antiporter |
| SAB2226 |  | divalent cation transporter |
| SAB2256 |  | cationic membrane transport protein |
| SAB2297 |  | cation efflux family protein |
| SAB2321 |  | sodium hydrogen exchanger family protein |
| SAB2424 | feoB | ferrous iron transport protein B |
| SAB2430 |  | exported protein |
| SAB2431 | copA | copper-transporting ATPase |
| SAB2559 |  | cobalt transport family protein |
| SAB2574 |  | high-affinity nickel-transport protein |
| Membrane transport | | |
| SAB0044 |  | membrane transport protein |
| SAB0046 |  | membrane transport protein |
| SAB0213 |  | membrane transport protein |
| SAB0284 |  | membrane efflux protein |
| SAB1301 |  | transmembrane efflux protein |
| SAB2061 |  | membrane transport protein |
| SAB2334 |  | membrane transport protein |
| SAB2335 |  | membrane transport protein |
| SAB2426 |  | membrane transport protein |
| Nucleosides, purines and pyrimidines | | |
| SAB0250 |  | nucleoside transporter permease |
| SAB0339 | pbuX | xanthine permease |
| SAB0471 | nupC | pyrimidine nucleoside transport protein |
| SAB0594 |  | nucleoside transport protein |
| SAB1063 | pyrP | uracil permease |
| SAB2125 |  | permease family protein |
| Other |  |  |
| SAB0081 |  | phosphonate ABC transporter permease |
| SAB0082 |  | phosphonate ABC transporter permease (pseudogene) |
| SAB0286 |  | glycerol-3-phosphate transporter |
| SAB0757 |  | lysE type translocator protein |
| SAB1160 | glpF | glycerol uptake facilitator |
| SAB1205 | mscL | large-conductance mechanosensitive channel |
| SAB1799 |  | ATP-binding ABC toxin transporter |
| Unknown substrate | | |
| SAB0083 |  | transport system protein |
| SAB0112 |  | nitrate/nitrite system ATP-binding ABC transporter |
| SAB0114 |  | ABC transporter permease |
| SAB0138 |  | ABC transporter ATP-binding protein |
| SAB0139 |  | ATP-binding ABC transporter |
| SAB0143 |  | ATP-binding ABC transporter |
| SAB0176 |  | dipeptide-binding ABC transporter |
| SAB0216 |  | ABC transporter ATP binding protein |
| SAB0245 |  | ABC transporter permease |
| SAB0246 |  | ATP-binding ABC transporter |
| SAB0301 |  | ATP-binding ABC transporter |
| SAB0408 |  | sodium-dependent transporter |
| SAB0411 |  | ATP-binding ABC transporter |
| SAB0412 |  | ATP-binding ABC transporter |
| SAB0593 |  | ATP-binding cassette transporter A |
| SAB0610 |  | ATP-binding ABC transport protein |
| SAB0611 |  | ABC transporter permease |
| SAB0622 |  | sugar efflux transporter |
| SAB0633 |  | ATP-binding ABC transporter protein |
| SAB0634 |  | ATP-binding ABC transporter protein |
| SAB0669 |  | ATP-binding ABC transporter protein |
| SAB0768 |  | ATP-binding ABC transporter protein |
| SAB0769 |  | ABC transporter permease protein |
| SAB0770 |  | substrate-binding ABC transporter protein |
| SAB0898 |  | ATP-binding ABC transport protein |
| SAB0943 |  | cation ATP-binding ABC transporter protein |
| SAB1247 |  | ATP-binding ABC transporter protein |
| SAB1379 |  | ABC transporter permease |
| SAB1380 |  | ABC transport protein |
| SAB1427 | mreB | ABC transporter protein |
| SAB1428 | mreA | ABC transporter protein |
| SAB1677 |  | transport protein |
| SAB1678 |  | transport protein |
| SAB1767 |  | ABC transporter |
| SAB1768 |  | ATP-binding ABC transporter |
| SAB1867 |  | transport protein |
| SAB1869 |  | ABC transporter protein |
| SAB1931 |  | ATP-binding ABC transporter protein |
| SAB2033 |  | ATP-binding ATP transporter protein |
| SAB2094 |  | ATP-binding ABC transporter |
| SAB2095 |  | ATP-binding ABC transporter |
| SAB2138 |  | membrane-bound transport protein |
| SAB2198 |  | sodium-dependent transporter |
| SAB2219 |  | sodium ABC transporter ATP-binding protein |
| SAB2238 |  | ATP-binding ABC transporter |
| SAB2239 |  | permease protein |
| SAB2309 |  | ATP-binding ABC transporter (pseudogene) |
| SAB2310 |  | truncated ATP-binding ABC transporter |
| SAB2316 |  | transporter protein |
| SAB2336 |  | ATP-binding ABC transporter |
| SAB2356 |  | transport protein |
| SAB2385 |  | transport protein |
| SAB2388 |  | ABC transporter |
| SAB2400 |  | ATP-binding ABC transporter |
| SAB2498 |  | ATP-binding ABC transporter |
| SAB2560 |  | ATP-binding ABC transporter |
| SAB2578 | vraD | ABC transporter |
| SAB2579 | vraE | ABC transporter permease |
| Unknown function | | |
| **Enzymes of unknown specificity** | | |
| SAB0062 |  | diaminopimelate decarboxylase protein |
| SAB0066 |  | acetoin (diacetyl) reductase |
| SAB0127 |  | hydrolase |
| SAB0179 |  | flavohemoprotein |
| SAB0207 |  | methyltransferase |
| SAB0253 |  | kinase |
| SAB0273 |  | luciferase family protein |
| SAB0287 |  | glyoxylase family protein |
| SAB0288 |  | luciferase-like monooxygenase |
| SAB0314 |  | GTP-binding protein |
| SAB0426 |  | acetyltransferase family protein |
| SAB0430 |  | lysine decarboxylase |
| SAB0438 |  | tetrapyrrole methylase family protein |
| SAB0454 |  | tetrapyrrole methylase |
| SAB0474 |  | creatine kinase |
| SAB0509 |  | cytosine deamidase (pseudogene) |
| SAB0552 |  | oxidoreductase |
| SAB0564 |  | haloacid dehalogenase hydrolase |
| SAB0565 |  | hydrolase |
| SAB0568 |  | esterase or lipase |
| SAB0637 |  | oxidoreductase |
| SAB0652 |  | oxidoreductase |
| SAB0668 |  | transmembrane sulfatase |
| SAB0715 |  | acetyltransferase |
| SAB0823 |  | NADH-dependent flavin oxidoreductase |
| SAB0840 |  | hydrolase |
| SAB0911 |  | hydrolase |
| SAB0920 |  | acetyltransferase family protein |
| SAB0955 |  | metallo-beta-lactamase superfamily protein |
| SAB0973 | suhB | inositol-1-monophosphatase |
| SAB0988 |  | methyltransferase |
| SAB1015 |  | nucleoside triphosphatase |
| SAB1039 |  | haloacid dehalgenase-like hydrolase |
| SAB1043 |  | S-adenosyl-methyltransferase |
| SAB1083 |  | phosphoprotein phosphatase |
| SAB1107 |  | GTP-binding protein |
| SAB1137 |  | metallo-beta-lactamase superfamily protein |
| SAB1163 |  | lysophospholipase |
| SAB1169 |  | GTP-binding protein |
| SAB1337 |  | GTP-ase protein (pseudogene) |
| SAB1361 |  | conserved oxidoreductase |
| SAB1363 |  | oxidoreductase |
| SAB1365 |  | metallo-beta-lactamase |
| SAB1405 |  | lipoate-protein ligase A |
| SAB1417 |  | metallo-beta-lactamase superfamily protein |
| SAB1457 | lepA | GTP binding protein |
| SAB1490 |  | deoxyribonuclease |
| SAB1497 |  | ATPase |
| SAB1533 |  | GTP-binding protein |
| SAB1566 |  | metallo-beta-lactamase superfamily protein |
| SAB1575 |  | aminotransferase |
| SAB1643 |  | aldo-keto reductase family protein |
| SAB1796 |  | bacterioferritin comigratory protein |
| SAB1917 |  | nitroreductase family protein |
| SAB2017 |  | amidase |
| SAB2018 | luxS | autoinducer-2 production protein |
| SAB2080 |  | oxidoreductase |
| SAB2205 |  | oxidoreductase |
| SAB2333 |  | carboxylesterase |
| SAB2359 |  | oxidoreductase (pseudogene) |
| SAB2386 |  | alkaline phosphatase |
| SAB2393 |  | glyoxylase family protein |
| SAB2403 |  | phosphinothricin N-acetyltransferase |
| SAB2433 |  | aminotransferase |
| SAB2442 |  | acetyltransferase |
| SAB2453 |  | short chain oxidoreductase |
| SAB2502 | phoB | alkaline phosphatase III precursor |
| SAB2536 |  | acetyltransferase |
| Hypothetical proteins | | |
| Conserved | | |
| SAB0007 |  | SAR0007 |
| SAB0014 |  | SACOL0014 |
| SAB0020 | yycH | SAV0020 |
| SAB0021 | yycI | SAS0021 |
| SAB0022 | yycJ | SAS0022 |
| SAB0024 |  | SAR0024 |
| SAB0025 |  | *S. pneumoniae R6* spr0491 |
| SAB0027 |  | SAS0059 |
| SAB0028 |  | MW0054 |
| SAB0030 |  | SAS0056 |
| SAB0085 |  | SAV0144 |
| SAB0107 |  | SAR0168 |
| SAB0115 |  | SAS0150 |
| SAB0116 |  | SAR0177 |
| SAB0130 |  | SAV0190 |
| SAB0137 |  | SAV0197 |
| SAB0158 |  | SAV0219 |
| SAB0167 |  | MW0204 |
| SAB0175 |  | MW0204 |
| SAB0177 |  | SAV0238 |
| SAB0223 |  | SAR0279 |
| SAB0226 |  | SAR0282 |
| SAB0234 |  | SAS0273 |
| SAB0271 |  | SAS0298 |
| SAB0275 |  | SAS0302 |
| SAB0276 |  | SAR0323 |
| SAB0285 |  | SAR0332 |
| SAB0298 |  | SAV0348 |
| SAB0305 |  | SAV0355 |
| SAB0311 |  | SAR0357 |
| SAB0312 |  | SAR0358 |
| SAB0313 |  | SAR0359 |
| SAB0319 |  | SAS0344 |
| SAB0322 |  | SAS0348 |
| SAB0323 |  | SAV0372 |
| SAB0325 |  | SAR0392 |
| SAB0326 |  | SAR0394 |
| SAB0335 |  | SACOL0455 |
| SAB0336 |  | SAS0362 |
| SAB0337 |  | SAS0363 |
| SAB0371 |  | SAS0379 |
| SAB0372 |  | SAS0380 |
| SAB0373 |  | SACOL0466 |
| SAB0401 |  | SACOL0491 |
| SAB0403 |  | SAR0453 |
| SAB0404 |  | SAR0454 |
| SAB0407 |  | SACOL0499 |
| SAB0428 |  | SAR0478 |
| SAB0432 |  | SAR0484 |
| SAB0435 |  | SAV0486 |
| SAB0436 |  | SAR0488 |
| SAB0437 |  | SAR0489 |
| SAB0441 |  | SAR0493 |
| SAB0443 |  | SAR0495 |
| SAB0455 |  | SAS0463 |
| SAB0456 |  | SAS0464 |
| SAB0458 |  | SAR0510 |
| SAB0473 |  | SAV0523 |
| SAB0481 |  | SAS0488 |
| SAB0483 |  | SAS0490 |
| SAB0492 |  | SAR0546 |
| SAB0506 |  | SAS0513 |
| SAB0510 |  | SAR0564 |
| SAB0511 |  | SAR0565 |
| SAB0516 |  | SAS0524 |
| SAB0517 |  | SAS0525 |
| SAB0518 |  | SAR0572 |
| SAB0522 |  | SAR0576 |
| SAB0532 |  | SAR0587 |
| SAB0537 |  | SACOL0633 |
| SAB0543 |  | SAS0552 |
| SAB0553 |  | SAR0609 |
| SAB0554 |  | SAR0610 |
| SAB0555 |  | SAV0603 |
| SAB0556 |  | SAR0612 |
| SAB0558 |  | SAV0606 |
| SAB0601 |  | SAS0617 |
| SAB0606 |  | SAR0667 |
| SAB0612 |  | SAR0673 |
| SAB0618 |  | SAR0680 |
| SAB0619 |  | SACOL0728 |
| SAB0620 |  | SA0626 |
| SAB0628 |  | SA0634 |
| SAB0629 |  | SAR0733 |
| SAB0630 |  | MW0643 |
| SAB0643 |  | SA0649 |
| SAB0666 |  | MW0679 |
| SAB0667 |  | SA0673 |
| SAB0674 |  | SAR0778 |
| SAB0678 |  | SA0683 |
| SAB0689 |  | SAR0791 |
| SAB0693 |  | SAR0795 |
| SAB0701 |  | SAR0803 |
| SAB0704 |  | SAR0806 |
| SAB0708 |  | SACOL0820 |
| SAB0709 |  | SA0711 |
| SAB0716 |  | SAR0817 |
| SAB0719 |  | SA0720 |
| SAB0720 |  | SAR0821 |
| SAB0721 |  | SAR0822 |
| SAB0743 |  | MW0763 |
| SAB0759 |  | SAR0859 |
| SAB0761 |  | SAV0830 |
| SAB0763 |  | SAR0863 |
| SAB0778 |  | SA0778 |
| SAB0788 |  | SAR0884 |
| SAB0790 |  | SAR0886 |
| SAB0792 |  | SACOL0928 |
| SAB0793 |  | SAR0889 |
| SAB0794 |  | SAR0890 |
| SAB0803 |  | SAS0807 |
| SAB0805 |  | SAR0901 |
| SAB0806 |  | SAR0902 |
| SAB0810 |  | SAV0944 |
| SAB0812 |  | SAR0907 |
| SAB0822 |  | MW0837 |
| SAB0841 |  | SAR0935 |
| SAB0846 |  | SAR0944 |
| SAB0864 |  | SAR0965 |
| SAB0868 |  | SAS0871 |
| SAB0869 |  | SAR0970 |
| SAB0870 |  | SACOL1008 |
| SAB0880 |  | SACOL1019 |
| SAB0881 |  | SAR0985 |
| SAB0903 |  | SAS0972 |
| SAB0907 |  | SA0893 |
| SAB0947 |  | SAV1081 |
| SAB0956 |  | SAR1064 |
| SAB0963 |  | SA0947 |
| SAB0972 |  | SAS1041 |
| SAB0975 |  | SAR1084 |
| SAB0976 |  | SAR1085 |
| SAB0977 |  | SAR1086 |
| SAB0984 |  | SAR1093 |
| SAB0987 |  | SAR1096 |
| SAB0990 |  | SAR1099 |
| SAB0991 |  | SAR1100 |
| SAB1005 |  | SAR1114 |
| SAB1016 |  | MW1035 |
| SAB1040 |  | SA1019 |
| SAB1041 |  | SAS1111 |
| SAB1042 |  | SAR1154 |
| SAB1051 |  | SAR1163 |
| SAB1052 |  | SAR1164 |
| SAB1053 |  | SAS1123 |
| SAB1058 |  | SAR1171 |
| SAB1061 |  | SA1040 |
| SAB1071 |  | SAR1183 |
| SAB1082 |  | SAR1194 |
| SAB1085 |  | SA1064 |
| SAB1087 |  | SAR1199 |
| SAB1090 |  | SAS1160 |
| SAB1100 |  | SA1079 |
| SAB1127 |  | SAS1199 |
| SAB1129 |  | SAR1241 |
| SAB1140 |  | SAR1254 |
| SAB1148 |  | SAR1262 |
| SAB1150 |  | SAS1222 |
| SAB1153 |  | SAV1291 |
| SAB1154 |  | SAR1268 |
| SAB1155 |  | SAR1269 |
| SAB1176 |  | *S. epidermidis* RP62A SERP0879 |
| SAB1177 |  | SAR1321 |
| SAB1183 |  | SAV1325 |
| SAB1189 |  | SAR1341 |
| SAB1199 |  | SAR1351 |
| SAB1208 |  | SAS1290 |
| SAB1232 |  | SAV1377 |
| SAB1246 |  | SAV1391 |
| SAB1259 |  | SAR1415 |
| SAB1260 |  | SAR1416 |
| SAB1264 |  | SACOL1444 |
| SAB1266 |  | SAR1422 |
| SAB1267 |  | SAR1423 |
| SAB1274 |  | SAV1419 |
| SAB1276 |  | SAR1434 |
| SAB1280 |  | SAS1368 |
| SAB1283 |  | SA1261 |
| SAB1284 |  | SA1262 |
| SAB1285 |  | SAR1443 |
| SAB1286 |  | SAS1374 |
| SAB1297 |  | SAP009 |
| SAB1306 |  | SACOL1480 |
| SAB1309 |  | SAR1456 |
| SAB1310 |  | SAR1457 |
| SAB1311 |  | SAR1458 |
| SAB1312 |  | SAR1459 |
| SAB1323 |  | SA1292 |
| SAB1326 |  | SA1295 |
| SAB1327 |  | SA1296 |
| SAB1342 |  | MW1368 |
| SAB1345 |  | SAR1491 |
| SAB1356 |  | MW1449 |
| SAB1357 |  | SAR1572 |
| SAB1371 |  | MW1463 |
| SAB1387 |  | SA1345 |
| SAB1398 |  | SAR1603 |
| SAB1410 |  | SA1368 |
| SAB1415 |  | MW1495 |
| SAB1418 |  | SAR1623 |
| SAB1420 |  | SAR1625 |
| SAB1422 |  | MW1502 |
| SAB1431 |  | SAR1636 |
| SAB1432 |  | SAV1560 |
| SAB1435 |  | SAR1640 |
| SAB1436 |  | MW1516 |
| SAB1442 |  | SAR1647 |
| SAB1448 |  | SAS1514 |
| SAB1449 |  | MW1529 |
| SAB1459 |  | SAR1664 |
| SAB1463 |  | SAS1528 |
| SAB1464 |  | SAR1669 |
| SAB1465 |  | SA1421 |
| SAB1467 |  | SAV1595 |
| SAB1469 |  | SAS1534 |
| SAB1470 |  | SAR1675 |
| SAB1471 |  | SAR1676 |
| SAB1476 |  | MW1555 |
| SAB1479 |  | SAR1687 |
| SAB1480 |  | SAR1688 |
| SAB1485 |  | MW1564 |
| SAB1486 |  | SAR1694 |
| SAB1488 |  | SAR1696 |
| SAB1491 |  | SAV1620 |
| SAB1496 |  | SAR1706 |
| SAB1515 |  | SAR1726 |
| SAB1537 |  | SAV1677 |
| SAB1545 |  | SAR1765 |
| SAB1562 |  | SAV1704 |
| SAB1564 |  | SAR1784 |
| SAB1571 |  | SAV1712 |
| SAB1576 |  | MW1660 |
| SAB1577 |  | SAS1645 |
| SAB1579 |  | SAR1798 |
| SAB1603 |  | SAR1821 |
| SAB1607 |  | SA1568 |
| SAB1609 |  | SAR1834 |
| SAB1617 |  | SAS1683 |
| SAB1620 |  | SA1581 |
| SAB1621 |  | SA1582 |
| SAB1623 |  | SA1584 |
| SAB1629 |  | SACOL1821 |
| SAB1657 |  | SAR1883 |
| SAB1658 |  | SAR1889 |
| SAB1659 |  | SAR1886 |
| SAB1660 |  | SAR1886 |
| SAB1661 |  | SAR1890 |
| SAB1662 |  | SAR1894 |
| SAB1762 |  | SAR1922 |
| SAB1776 |  | MW1784 |
| SAB1777 |  | SAR1935 |
| SAB1778 |  | SAR1936 |
| SAB1795 |  | SAR1952 |
| SAB1800 |  | MW1807 |
| SAB1807 |  | SA1692 |
| SAB1808 |  | SAR1966 |
| SAB1809 |  | SAS1799 |
| SAB1810 |  | SAR1968 |
| SAB1812 |  | SAR1970 |
| SAB1830 |  | SAR1989 |
| SAB1831 |  | SACOL1177 |
| SAB1837 |  | *S. aureus* unknown gb|AAM26286.1| |
| SAB1847 |  | *S. aureus* gb|AAM26287.1| |
| SAB1852 |  | SA1731 |
| SAB1854 |  | SAR2010 |
| SAB1858 |  | SAR2014 |
| SAB1859 |  | SAR2015 |
| SAB1861 |  | SAR2017 |
| SAB1865 |  | SAR2021 |
| SAB1919 |  | SAR2121 |
| SAB1928 |  | SAS1949 |
| SAB1930 |  | SAR2133 |
| SAB1937 |  | SAR2139 |
| SAB1947 |  | SA1867 |
| SAB1948 |  | SAV2063 |
| SAB1953 |  | SAS1973 |
| SAB1959 |  | SA1878 |
| SAB1971 |  | SA1890 |
| SAB1973 |  | SA1892 |
| SAB1982 |  | SAR2186 |
| SAB1998 |  | MW2038 |
| SAB1999 |  | SAR2203 |
| SAB2000 |  | SAR2204 |
| SAB2001 |  | SAR2205 |
| SAB2007 |  | SAR2211 |
| SAB2013 |  | SAR2217 |
| SAB2014 |  | SAS2033 |
| SAB2024 |  | SAV2140 |
| SAB2028 |  | SAS2047 |
| SAB2032 |  | SAR2240 |
| SAB2052 |  | SA1974 |
| SAB2059 |  | SAS2079 |
| SAB2060 |  | SAR2270 |
| SAB2062 |  | SAV2181 |
| SAB2067 |  | MW2112 |
| SAB2084 |  | *L. innocua* Clip11262 lin0465 |
| SAB2131 |  | SAR2343 |
| SAB2154 |  | MW2200 |
| SAB2174 |  | SA2095 |
| SAB2181 |  | SAR2390 |
| SAB2197 |  | SA2111 |
| SAB2214 |  | SACOL2330 |
| SAB2227 |  | MW2269 |
| SAB2229 |  | SAR2435 |
| SAB2242 |  | SAV2363 |
| SAB2250 |  | SA2160 |
| SAB2251 |  | SAR2460 |
| SAB2255 |  | SA2165 |
| SAB2261 |  | SACOL2379 |
| SAB2268 |  | SAV2389 |
| SAB2272 |  | SA2181 |
| SAB2282 |  | SAR2491 |
| SAB2285 |  | SAR2495 |
| SAB2287 |  | SAS2298 |
| SAB2305 |  | SAR2514 |
| SAB2319 |  | SACOL2440 |
| SAB2324 |  | SAR2532 |
| SAB2351 |  | SAV2468 |
| SAB2353 |  | SAS2361 |
| SAB2354 |  | SAR2558 |
| SAB2355 |  | MW2396 |
| SAB2357 |  | SAR2561 |
| SAB2381 |  | SAS2394 |
| SAB2392 |  | SAR2598 |
| SAB2395 |  | SAR2601 |
| SAB2399 |  | SAR2607 |
| SAB2401 |  | MW2447 |
| SAB2410 |  | SAR2616 |
| SAB2411 |  | SAR2617 |
| SAB2447 |  | SAR2654 |
| SAB2448 |  | MW2495 |
| SAB2455 |  | SACOL2597 |
| SAB2458 |  | SAS2470 |
| SAB2460 |  | SACOL2601 |
| SAB2463 |  | SAR2668 |
| SAB2466 |  | MW2510 |
| SAB2467 |  | SACOL2609 |
| SAB2469 |  | SAR2674 |
| SAB2473 |  | SAR2678 |
| SAB2488 |  | MW2533 |
| SAB2493 |  | SAS2505 |
| SAB2522 |  | SAR2726 |
| SAB2526 |  | SAR2730 |
| SAB2527 |  | SAV2652 |
| SAB2534 |  | SAS2545 |
| SAB2562 |  | SAS2571 |
| SAB2563 |  | SAV2687 |
| SAB2565 |  | SACOL2713 |
| SAB2568 |  | SACOL2716 |
| SAB2585 |  | SA2498 |
| Hypothetical proteins | | |
| SAB0003 |  | SAR0003 |
| SAB0013 |  | SAR0013 |
| SAB0029 |  | SAS0055 |
| SAB0034 |  | *S. aureus* unknown gb|AAL26677.1| |
| SAB0035 |  | *S. aureus* unknown gb|AAL26678.1| |
| SAB0036 |  | *S. aureus* unknown gb|AAL26680.1| |
| SAB0037 |  | SAR0104 |
| SAB0040 |  | SAR0106 |
| SAB0048 |  | MW0082 |
| SAB0063 |  | SAR0127 |
| SAB0065 |  | MW0099 |
| SAB0110 |  | SA0164 |
| SAB0111 |  | SAR0172 |
| SAB0134 |  | SA0188 |
| SAB0136 |  | SA0190 |
| SAB0140 |  | SA0194 |
| SAB0141 |  | SAV0201 |
| SAB0142 |  | MW0177 |
| SAB0150 |  | MW0188 |
| SAB0155 |  | SAR2752 |
| SAB0166 |  | SAS0203 |
| SAB0168 |  | SAS0205 |
| SAB0188 |  | SAR0244 |
| SAB0206 |  | SACOL0412 |
| SAB0212 |  | SAR0270 |
| SAB0220 |  | SACOL0268 |
| SAB0221 |  | SACOL0269 |
| SAB0229 |  | SA0277 |
| SAB0230 |  | SACOL0278 |
| SAB0231 |  | SA0279 |
| SAB0232 |  | SA0280 |
| SAB0235 |  | SACOL0283 |
| SAB0237 |  | SAS0269 |
| SAB0238 |  | SAS0274 |
| SAB0239 |  | SAS0276 |
| SAB0240 |  | SA0291 |
| SAB0315 |  | SAR0361 |
| SAB0321 |  | SAV0370 |
| SAB0324 |  | SAR0391 |
| SAB0327 |  | SAV0377 |
| SAB0328 |  | SACOL0449 |
| SAB0329 |  | SACOL0450 |
| SAB0365 |  | SAS0370 |
| SAB0367 |  | SAS0372 |
| SAB0368 |  | SAS0373 |
| SAB0369 |  | SAS0377 |
| SAB0374 |  | SACOL0467 |
| SAB0391 |  | SAR0438 |
| SAB0392 |  | SAV0438 |
| SAB0393 |  | SAR0442 |
| SAB0394 |  | SACOL0482 |
| SAB0395 |  | SACOL0483 |
| SAB0396 |  | MW0399 |
| SAB0397 |  | SACOL0487 |
| SAB0398 |  | SAR0447 |
| SAB0406 |  | SAR0456 |
| SAB0416 |  | SAS0424 |
| SAB0417 |  | SACOL0510 |
| SAB0527 |  | SA0535 |
| SAB0528 |  | SAR0583 |
| SAB0529 |  | SAS016 |
| SAB0548 |  | SACOL0651 |
| SAB0549 |  | SACOL0652 |
| SAB0550 |  | SACOL0653 |
| SAB0551 |  | MW0562 |
| SAB0602 |  | SAR0663 |
| SAB0605 |  | SAR0666 |
| SAB0607 |  | SAR0668 |
| SAB0617 |  | SAR0679 |
| SAB0625 |  | SAR0729 |
| SAB0627 |  | SAR0731 |
| SAB0631 |  | SA0637 |
| SAB0680 |  | SACOL0790.1 |
| SAB0691 |  | MW0701 |
| SAB0710 |  | SAR0811 |
| SAB0718 |  | SACOL0786 |
| SAB0750 |  | SAR0849 |
| SAB0751 |  | SAR0850 |
| SAB0755 |  | SAR0855 |
| SAB0758 |  | SAR0858 |
| SAB0767 |  | SAS0777 |
| SAB0772 |  | SAR0874 |
| SAB0820 |  | SAV0953 |
| SAB0871 |  | SAR0972 |
| SAB0885 |  | MW0900 |
| SAB0892 |  | SAS0959 |
| SAB0900 |  | SAR1007 |
| SAB0921 |  | SAR1028 |
| SAB0929 |  | SACOL0412 |
| SAB0948 |  | MW0964 |
| SAB0951 |  | SA0936 |
| SAB0986 |  | SAR1095 |
| SAB1017 |  | SAS1087 |
| SAB1019 |  | SAV1156 |
| SAB1024 |  | SACOL1171 |
| SAB1025 |  | SAS1095 |
| SAB1026 |  | SA1005 |
| SAB1028 |  | MW1045 |
| SAB1029 |  | SA1008 |
| SAB1070 |  | SAR1182 |
| SAB1149 |  | SAS1221 |
| SAB1173 |  | SAV1312 |
| SAB1174 |  | SAR1306 |
| SAB1179 |  | SAR1327 |
| SAB1184 |  | SA1162 |
| SAB1190 |  | SAR1342 |
| SAB1198 |  | SAR1350 |
| SAB1209 |  | SA1186 |
| SAB1231 |  | SAV1376 |
| SAB1233 |  | MW1266 |
| SAB1257 |  | SAS1342 |
| SAB1308 |  | SAR1455 |
| SAB1315 |  | SAR1462 |
| SAB1334 |  | SAR1481 |
| SAB1350 |  | SAS1429 |
| SAB1351 |  | SAV1486 |
| SAB1362 |  | SAS1441 |
| SAB1374 |  | *L. monocytogenes* str. 4b F2365 gene LMOf2365_1117 |
| SAB1375 |  | *L. monocytogenes* str. 4b F2365 gene LMOf2365_1117 |
| SAB1377 |  | *L. monocytogenes* str. 4b F2365 gene LMOf2365_1116 |
| SAB1411 |  | SAR1616 |
| SAB1444 |  | SAR1649 |
| SAB1456 |  | SACOL1170 |
| SAB1519 |  | MW1601 |
| SAB1536 |  | SACOL1723 |
| SAB1583 |  | SAS1651 |
| SAB1598 |  | SAS1664 |
| SAB1612 |  | SAR1837 |
| SAB1634 |  | SAR1858 |
| SAB1636 |  | SA1596 |
| SAB1653 |  | SAR1879 |
| SAB1656 |  | SAR1882 |
| SAB1674 |  | SAS1737 |
| SAB1675 |  | SA1633 |
| SAB1676 |  | MW1758 |
| SAB1680 |  | MW1762 |
| SAB1681 |  | MW1763 |
| SAB1682 |  | SACOL1877 |
| SAB1683 |  | MW1765 |
| SAB1685 |  | MW1766 |
| SAB1692 |  | SAR2705 |
| SAB1693 |  | SAR1913 |
| SAB1694 |  | SAV1823 |
| SAB1740 |  | SACOL0344 |
| SAB1742 |  | SACOL0342 |
| SAB1771 |  | SAS1760a |
| SAB1786 |  | SACOL1910 |
| SAB1787 |  | SAR1944 |
| SAB1819 |  | SA1703 |
| SAB1822 |  | SAS1812 |
| SAB1846 |  | SAR2002 |
| SAB1879 |  | *Exiguobacterium sp.* 255-15 drug permease superfamily |
| SAB1885 |  | SAR2115 |
| SAB1889 |  | SAR1129 |
| SAB1909 |  | SAV2025 |
| SAB1954 |  | SAR2157 |
| SAB1969 |  | SAR2173 |
| SAB1970 |  | SA1889 |
| SAB1979 |  | SAR2183b |
| SAB1994 |  | SAR2198 |
| SAB2010 |  | SAR2214 |
| SAB2015 |  | SAR2219 |
| SAB2016 |  | SAR2220 |
| SAB2025 |  | SAR2229 |
| SAB2035 |  | MW2081 |
| SAB2049 |  | SAV2167 |
| SAB2054 |  | SAR2264 |
| SAB2079 |  | MW2125 |
| SAB2088 |  | SAV2208 |
| SAB2127 |  | SAS2146 |
| SAB2129 |  | SACOL2247 |
| SAB2136 |  | SAV2264 |
| SAB2137 |  | SA2060 |
| SAB2139 |  | SAR2351 |
| SAB2153 |  | SACOL2274 |
| SAB2168 |  | SAV2296 |
| SAB2169 |  | SAV2297 |
| SAB2177 |  | MW2223 |
| SAB2184 |  | MW2227 |
| SAB2185 |  | SAR2392 |
| SAB2191 |  | SAR2398 |
| SAB2199 |  | SAR2407 |
| SAB2202 |  | SAS2218 |
| SAB2221 |  | MW2264 |
| SAB2228 |  | SAR2434 |
| SAB2247 |  | SAV2367 |
| SAB2260 |  | SAR2468 |
| SAB2266 |  | SACOL2385 |
| SAB2288 |  | SA2196 |
| SAB2312 |  | SAV2430 |
| SAB2317 |  | SACOL2438 |
| SAB2318 |  | SA2224 |
| SAB2339 |  | SAS2348 |
| SAB2340 |  | SA2246 |
| SAB2341 |  | SACOL2466 |
| SAB2352 |  | SACOL2478 |
| SAB2362 |  | SAR2568 |
| SAB2363 |  | SAR2569 |
| SAB2365 |  | SACOL2494 |
| SAB2366 |  | SAV2483 |
| SAB2367 |  | SAV2484 |
| SAB2376 |  | SACOL2513 |
| SAB2382 |  | SACOL2519 |
| SAB2384 |  | MW2430 |
| SAB2417 |  | SAR2623 |
| SAB2421 |  | *S. pyogenes* M49 gene SpyoM01000968 |
| SAB2425 |  | SAR2631 |
| SAB2436 |  | SAR2645 |
| SAB2438 |  | MW2486 |
| SAB2446 |  | SAR2653 |
| SAB2449 |  | SAS2462 |
| SAB2450 |  | SAR2656 |
| SAB2451 |  | SAR2657 |
| SAB2452 |  | SAV2578 |
| SAB2461 |  | SAR2666 |
| SAB2462 |  | SAR2667 |
| SAB2468 |  | SAR2673 |
| SAB2483 |  | SAR2688 |
| SAB2485 |  | SAR2689 |
| SAB2520 |  | SAR2724 |
| SAB2525 |  | SAR2729 |
| SAB2532 |  | SAR2738 |
| SAB2533 |  | SACOL2681 |
| SAB2558 |  | SACOL2706 |
| SAB2567 |  | SACOL2715 |
| SAB2569 |  | SACOL2717 |
| SAB2575 |  | SA2490 |
| Mobile element-associated hypothetical proteins | | |
| SAB0343 |  | *S. aureus* Orf20 gb|AAG29617.1| |
| SAB0344 |  | *S. aureus* Orf19 gb|AAG29616.1| |
| SAB0345 |  | *S. aureus* Orf18 gb|AAG29615.1| |
| SAB0346 |  | SAR0370 |
| SAB0347 |  | SAR0371 |
| SAB0348 |  | *S. aureus* Orf17 gb|AAG29614.1| |
| SAB0349 |  | *S. aureus* Orf16 gb|AAG29613.1| |
| SAB0350 |  | *S. aureus* Orf15 gb|AAG29612.1| |
| SAB0351 |  | SA1828 |
| SAB0352 |  | *S. aureus* Orf12 gb|AAG29609.1| |
| SAB0353 |  | *S. aureus* Orf11 gb|AAG29608.1| |
| SAB0354 |  | *S. aureus* Orf10 gb|AAG29607.1| |
| SAB0355 |  | *S. aureus* Orf9 gb|AAG29606.1| |
| SAB0356 |  | *S. aureus* Orf8 gb|AAG29605.1| |
| SAB0357 |  | *S. aureus* Orf7 gb|AAG29604.1| |
| SAB0358 |  | *S. aureus* Orf6 gb|AAG29603.1| |
| SAB0359 |  | *S. aureus* Orf5 gb|AAG29602.1| |
| SAB0361 |  | SAR0384 |
| SAB0362 |  | *S. aureus* Orf3 gb|AAG29600.1| |
| SAB1294 |  | *S. aureus* pI9789::Tn552 gb|AAF63254.1| |
| SAB1295 |  | *S. aureus* pC55s gb|AAL05956.1| |
| SAB1474 |  | SACOL1658 |
| SAB1707 |  | *S. aureus* phage phi 11 gb|AAL82276.1| |
| SAB1708 |  | *S. aureus* bacteriophage phi 53 gb|AAM49606.1| |
| SAB1709 |  | *S. aureus* bacteriophage phi 53 gb|AAM49605.1| |
| SAB1710 |  | SAV0904 |
| SAB1711 |  | *S. aureus* phage phi 11 gb|AAL82272.1| |
| SAB1712 |  | *S. aureus* phage phi 11 gb|AAL82271.1| |
| SAB1713 |  | SAV0901 |
| SAB1714 |  | *S. aureus* phage phi 11 gb|AAL82269.1| |
| SAB1715 |  | *S. aureus* phage phi 11 gb|AAL82268.1| |
| SAB1716 |  | SAV0898 |
| SAB1717 |  | *S. aureus* phage phi 11 gb|AAL82266.1| |
| SAB1718 |  | *S. aureus* phage phi 11 gb|AAL82265.1| |
| SAB1719 |  | SAV0895 |
| SAB1720 |  | SAV0894 |
| SAB1721 |  | *S. aureus* phage phi 11 gb|AAL82263.1| |
| SAB1723 |  | SAV0890 |
| SAB1724 |  | SAV0888 |
| SAB1725 |  | *S. aureus* phage phi 11 gb|AAL82259.1| |
| SAB1727 |  | *S. aureus* Orf5 gb|AAG29602.1| |
| SAB1730 |  | SACOL0358 |
| SAB1731 |  | *S. aureus* phage phi 11 gb|AAL82254.1| |
| SAB1732 |  | SA1784 |
| SAB1733 |  | SAR2074 |
| SAB1734 |  | *S. aureus* prophage phiPV83 ref|NP_061617.1| |
| SAB1735 |  | SA1786 |
| SAB1736 |  | SA1787 |
| SAB1737 |  | Bacteriophage 77 ref|P_958651.1| |
| SAB1738 |  | *S. aureus* phage phi 11 gb|AAL82246.1| |
| SAB1739 |  | *S. aureus* phage phi 11 gb|AAL82245.1| |
| SAB1743 |  | *B. cereus* ZK phage-related protein ref|YP_085033.1| |
| SAB1744 |  | SAV0866 |
| SAB1746 |  | SACOL0338 |
| SAB1747 |  | SACOL0337 |
| SAB1748 |  | *S. aureus* prophage phiPV83 ref|NP_061605.1| |
| SAB1749 |  | SA1798 |
| SAB1751 |  | *S. aureus* phi PVL ref|NP_061603.1| |
| SAB1754 |  | SAV0858 |
| SAB1756 |  | *B. cereus* ATCC 14579 phage protein ref|NP_833431.1| |
| SAB1884 |  | SAR2113 |
| SAB1894 |  | SAV0800 |
| SAB1895 |  | *S. aureus* Orf4 gb|AAC28955.2| |
| SAB1896 |  | *S. aureus* Orf7 gb|AAG29604.1| |
| SAB1897 |  | *S. aureus* Orf8 gb|AAG29605.1| |
| SAB1898 |  | *S. aureus* Orf9 gb|AAG29606.1| |
| SAB1899 |  | *S. aureus* Orf10 gb|AAG29607.1| |
| SAB1902 |  | SAV2019 |
| SAB1903 |  | SAR0376 |
| SAB1904 |  | SAR0375 |
| SAB1905 |  | SAR0374 |
| SAB1906 |  | *S. aureus* Orf16 gb|AAG29613.1| |
| SAB1908 |  | MW0749 |
| SAB1911 |  | *S. aureus* Orf22 gb|AAC28967.2| |
| Hypothetical pseudogenes | | |
| SAB0087 |  | SAR0148 |
| SAB0106 |  | SAR0167 |
| SAB0233 |  | SAV0293 |
| SAB0236 |  | MW0271 |
| SAB0366 |  | MW0372 |
| SAB0399 |  | SA0408 |
| SAB0571 |  | SA0575 |
| SAB0765 |  | SAR0865 |
| SAB0766 |  | SAR0867 |
| SAB0787 |  | SAR0883 |
| SAB0845 |  | SAR0943 |
| SAB0918 |  | SAV1050 |
| SAB1055 |  | SAR1167 |
| SAB1077 |  | SAR1189 |
| SAB1142 |  | SAR1256 |
| SAB1196 |  | SAV1338 |
| SAB1355 |  | SAV1494 |
| SAB1665 |  | MW1749 |
| SAB1666 |  | MW1749 |
| SAB1878 |  | *Exiguobacterium sp.*drug/metabolite permease superfamily |
| SAB1887 |  | SAV0804 |
| SAB2209 |  | SA2123 |
| SAB2258 |  | SA2168 |
| SAB2331 |  | SAR2539 |
| SAB2368 |  | MW2407 |
| SAB2407 |  | SACOL2548 |
| SAB2408 |  | SACOL2548 |
| SAB2454 |  | SAS2466 |
| SAB2477 |  | SA2397 |
| SAB2530 |  | SAV2655 |
| SAB2553 |  | SACOL2701 |
| Unique genes | | |
| SAB0064 |  | unknown |
| SAB0079 |  | unknown |
| SAB0080 |  | unknown |
| SAB0268 |  | unknown |
| SAB0269 |  | unknown |
| SAB0270 |  | unknown |
| SAB0282 |  | unknown |
| SAB0291 |  | unknown |
| SAB0310 |  | unknown |
| SAB0334 |  | unknown |
| SAB0370 |  | unknown |
| SAB0375 |  | unknown |
| SAB0677 |  | unknown |
| SAB0684 |  | unknown |
| SAB0725 |  | unknown |
| SAB0739 |  | unknown |
| SAB0740 |  | unknown |
| SAB0771 |  | unknown |
| SAB0784 |  | unknown |
| SAB0826 |  | unknown |
| SAB0831 |  | unknown |
| SAB0847 |  | unknown |
| SAB0916 |  | unknown |
| SAB0946 |  | unknown |
| SAB1038 |  | unknown |
| SAB1175 |  | unknown |
| SAB1238 |  | unknown |
| SAB1339 |  | unknown |
| SAB1370 |  | unknown |
| SAB1378 |  | unknown |
| SAB1381 |  | unknown |
| SAB1382 |  | unknown |
| SAB1383 |  | unknown |
| SAB1495 |  | unknown |
| SAB1565 |  | unknown |
| SAB1684 |  | unknown |
| SAB1688 |  | unknown |
| SAB1689 |  | unknown |
| SAB1695 |  | unknown |
| SAB1702 |  | unknown |
| SAB1750 |  | unknown |
| SAB1752 |  | unknown |
| SAB1758 |  | unknown |
| SAB1759 |  | unknown |
| SAB1761 |  | unknown |
| SAB1772 |  | unknown |
| SAB1844 |  | unknown |
| SAB1881 |  | unknown |
| SAB1886 |  | unknown |
| SAB1888 |  | unknown |
| SAB1890 |  | unknown |
| SAB1891 |  | unknown |
| SAB1893 |  | unknown |
| SAB1900 |  | unknown |
| SAB1901 |  | unknown |
| SAB1907 |  | unknown |
| SAB1918 |  | unknown |
| SAB1933 |  | unknown |
| SAB2089 |  | unknown |
| SAB2158 |  | unknown |
| SAB2171 |  | unknown |
| SAB2182 |  | unknown |
| SAB2217 |  | unknown |
| SAB2246 |  | unknown |
| SAB2259 |  | unknown |
| SAB2277 |  | unknown |
| SAB2311 |  | unknown |
| SAB2337 |  | unknown |
| SAB2364 |  | unknown |
| SAB2370 |  | unknown |
| SAB2387 |  | unknown |
| SAB2396 |  | unknown |
| SAB2440 |  | unknown |
| SAB2459 |  | unknown |
| SAB2484 |  | unknown |
| SAB2496 |  | unknown |
| SAB2545 |  | unknown |
| SAB2547 |  | unknown |
| SAB2570 |  | unknown |
| SAB2576 |  | unknown |
